# A glimpse at the intricate mosaic of ethnicities from Mesopotamia: Paternal lineages of the Northern Iraqi Arabs, Kurds, Syriacs, Turkmens and Yazidis

**DOI:** 10.1371/journal.pone.0187408

**Published:** 2017-11-03

**Authors:** Serkan Dogan, Cemal Gurkan, Mustafa Dogan, Hasan Emin Balkaya, Ramazan Tunc, Damla Kanliada Demirdov, Nihad Ahmed Ameen, Damir Marjanovic

**Affiliations:** 1 Department of Genetics and Bioengineering, International Burch University, Sarajevo, Bosnia and Herzegovina; 2 Turkish Cypriot DNA Laboratory, Committee on Missing Persons in Cyprus Turkish Cypriot Member Office, Nicosia (North Cyprus), Turkey; 3 Dr. Fazıl Küçük Faculty of Medicine, Eastern Mediterranean University, Famagusta (North Cyprus), Turkey; 4 Department of Biology, College of Education, University of Salahaddin, Erbil, Iraq; 5 Institute for Anthropological Research, Zagreb, Croatia; Harvard Medical School, UNITED STATES

## Abstract

Widely considered as one of the cradles of human civilization, *Mesopotamia* is largely situated in the Republic of Iraq, which is also the birthplace of the Sumerian, Akkadian, Assyrian and Babylonian civilizations. These lands were subsequently ruled by the Persians, Greeks, Romans, Arabs, Mongolians, Ottomans and finally British prior to the independence. As a direct consequence of this rich history, the contemporary Iraqi population comprises a true mosaic of different ethnicities, which includes Arabs, Kurds, Turkmens, Assyrians, and Yazidis among others. As such, the genetics of the contemporary Iraqi populations are of anthropological and forensic interest. In an effort to contribute to a better understanding of the genetic basis of this ethnic diversity, a total of 500 samples were collected from Northern Iraqi volunteers belonging to five major ethnic groups, namely: Arabs (*n* = 102), Kurds (*n* = 104), Turkmens (*n* = 102), Yazidis (*n* = 106) and Syriacs (*n* = 86). 17-loci Y-STR analyses were carried out using the AmpF*l*STR Yfiler system, and subsequently *in silico* haplogroup assignments were made to gain insights from a molecular anthropology perspective. Systematic comparisons of the paternal lineages of these five Northern Iraqi ethnic groups, not only among themselves but also in the context of the larger genetic landscape of the Near East and beyond, were then made through the use of two different genetic distance metric measures and the associated data visualization methods. Taken together, results from the current study suggested the presence of intricate Y-chromosomal lineage patterns among the five ethic groups analyzed, wherein both interconnectivity and independent microvariation were observed in parallel, albeit in a differential manner. Notably, the novel Y-STR data on Turkmens, Syriacs and Yazidis from Northern Iraq constitute the first of its kind in the literature. Data presented herein is expected to contribute to further population and forensic investigations in Northern Iraq in particular and the Near East in general.

## Introduction

**Fig 1:**
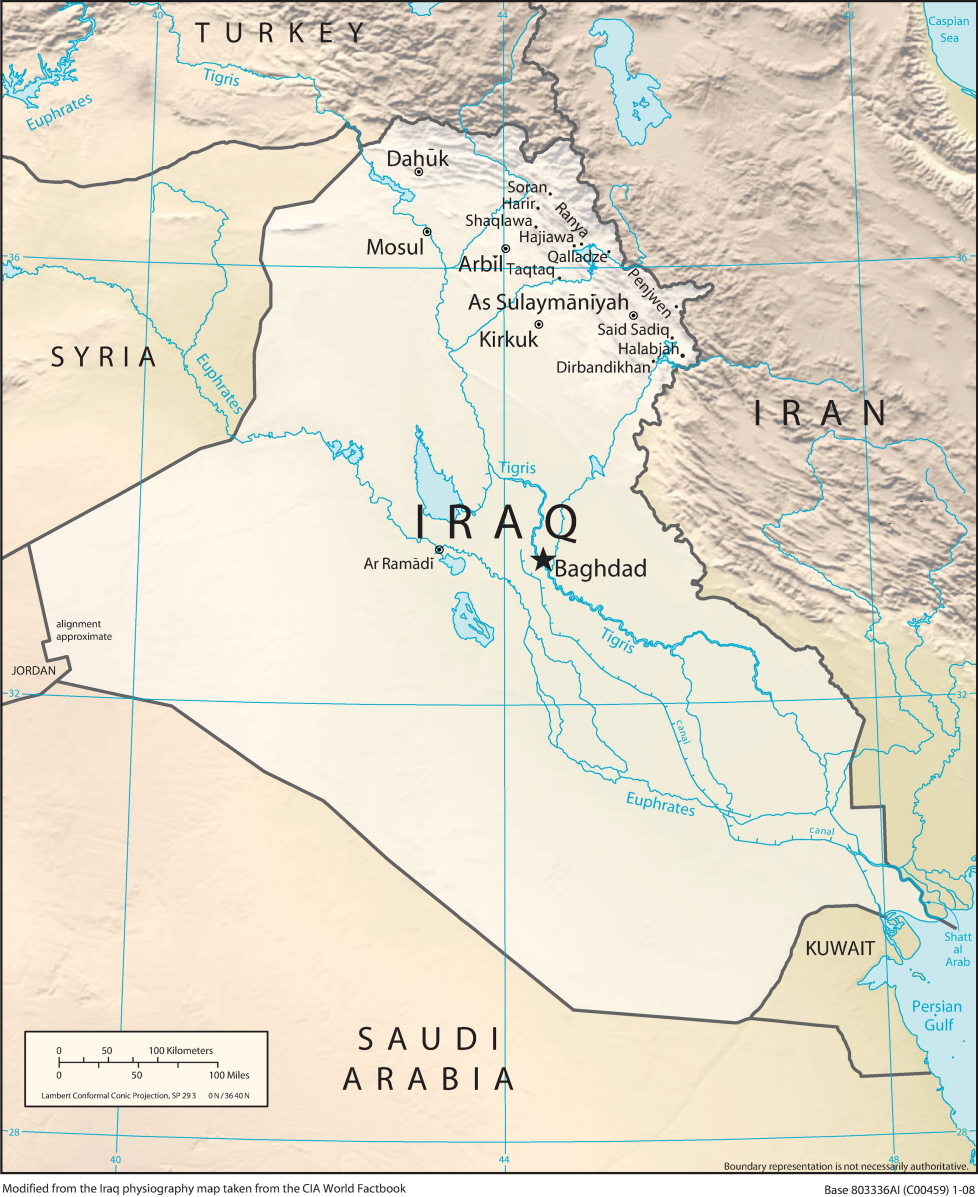
Immediate geographic location of the Republic of Iraq (A modified version of the Iraq physiography map taken from the CIA’s World Factbook). Only the self-reported birthplaces of the volunteers (e.g. cities, towns, etc.) are shown on the map.

Often considered as one of the cradles of human civilization, *Mesopotamia* encompasses the ancient fertile lands defined by the Tigris and Euphrates river systems. Today, these lands are largely situated in Iraq, which shares borders with Jordan to the west, Syria to the north-west, Turkey to the north, Kuwait and Saudi Arabia to the south and Iran to the east (Fig 1). Iraq has a population of ~40 million, comprising mainly of Arabs and Kurds, but also the Assyrians, Turkmens, Shabakis, Yazidis, Armenians, Mandeans, Circassians, and Kawliya minorities. Accordingly, population genetics of Iraqis is of interest not only because of this ethnic diversity, but also due to the fact that the country was home to the Sumerian, Akkadian, Assyrian and Babylonian civilizations, and ruled by the Persians, Greeks, Arabs, Mongolians, Ottomans and British [1, 2].

Among the Northern Iraqi populations, Arabs are regarded as a panethnicity that largely adhere to different sects of Islam and actually native to an immense geography spanning from the Atlantic coast of North Africa to the Horn of Africa in the East, as well as the entire Arabian Peninsula and a large portion of the Near East. Iraqi Arabs have been the majority in the region since the 3^rd^ century AC, when the first Arab Kingdom was formed outside of Arabian Peninsula [3]. Arabs are estimated to comprise 75–80% of the entire Iraqi population, while Kurds, the largest ethnic minority in Iraq, comprise 15–20%, and furthermore the latter also constitutes the majority in Northern Iraq [1]. Kurds are of Indo-European origin, and speak the Kurdish language, a subgroup of Northwestern Iranian languages [4]. Kurdish people are considered to be one of the native inhabitants of Iraq, although there is no strict description on their precise origin [4]. Turkmens, also known as Turcomans, largely exists as a prominent minority beyond the immediate Southeastern borders of Modern Turkey, across Northern Syria, Northern Iraq and Northeastern Iran. Iraqi Turkmens are the third largest ethnic group in the country and mostly live in an area extending from northwest to southeast of Iraq, including the provinces of Mosul, Erbil and Kirkuk [5]. As in the case of other ethnic minorities in Iraq, precise population data are not available, but Iraqi Turkmens are estimated to constitute between 3% to 13% of the entire Iraqi population [6]. Yazidis, also known as Yezidis, are an ethnoreligous group largely inhabiting Northern Syria and Northern Iraq. A distinguishing feature of Yazidis among the other Mesopotamian populations is their religion, Yazidism or Yazdanism, which is linked with the ancient Mesopotamian religions and combines aspects of Zoroastrianism, Islam, Christianity and Judaism [7]. Finally, Syriacs, also known as Assyrians, Chaldeans and Arameans are also an ethnoreligious group native to Middle East, largely inhabiting a region from across modern Syria, Iraq and Iran. Syriacs are Semitic people that speak modern Arameic and adhere to different sects of Christianity. Syriacs are also an indigenous ethnic group of Modern Iraq, and are known to inhabit major cities, as well as in the mountainous regions to the east of Mosul, near Dohuk and Akra [8]. Recent estimates suggest that there are 133,000 Assyrians in Iraq, or less than 1% of total population [9].

At least from a population genetics perspective, the contemporary Iraqi populations remain almost unexplored. In such cases, investigations on the paternal and maternal lineages, which are based on the Y-chromosome and mitochondrial DNA, respectively, can provide very useful primers [10]. On the one hand, variations among different paternal lineages are best described in terms of Y-chromosomal haplogoups, which are in turn defined by unique combinations of Y-chromosomal single nucleotide polymorphisms (Y-SNPs). On the other hand, Y-chromosomal short tandem repeat markers (Y-STRs) are another highly useful set of markers and offer further advantages through their higher mutation rates compared to Y-SNPs, hence allowing more detailed investigations within each haplogroup. Over the last decade, *in silico* Y-chromosomal haplogroup assignment tools have also become available, which allow haplogroup assignment for a given paternal lineage based on Y-STR data alone and with accuracies over 95% [11].

The aim of the current study was to contribute to a better understanding of the genetic basis of the Northern Iraqi ethnic diversity through a comparative analysis of the paternal lineages belonging to five of the most populous ethnicities from the region. To achieve this, a total of 500 samples were collected from the Arab, Kurd, Turkmen, Yazidi and Syriac communities, and each was analyzed by 17-loci Y-STR haplotyping and then *in silico* haplogroup assignment. Systematic comparisons of the paternal lineages, not only among themselves but also in the context of the larger genetic landscape of the Near East and beyond, revealed the presence of intricate Y-chromosomal lineage patterns among the five ethic groups analyzed, wherein both interconnectivity and independent microvariation were observed in parallel, albeit in a differential manner.

## Materials and methods

A total of 500 buccal swab samples were collected from healthy and unrelated individuals, each of whom was aged 18 and above and belonged to one of the five major ethnic groups in Northern Iraq as follows: Arabs (*n* = 102), Kurds (*n* = 104), Syriacs (*n* = 86), Turkmens (*n* = 102) and Yazidis (*n* = 106). Determination of ethnicity was based on that of both parents. While the Arab, Kurdish and Turkmen samples were largely collected from among the students of the Salahaddin University in Erbil, the Syriac and Yazidi samples were mostly collected at various refugee camps in Erbil. Yet, the actual birthplaces of the volunteers encompassed a wider geography from Northern Iraq as depicted in Fig 1. All samples were collected with written informed consent and according to the principles of the Helsinki Declaration of the World Medical Association. Local translators were also available to ensure informed consent. Approvals for the study were provided by the Ethics Committee of the Department of Genetics and Bioengineering, as well as that of the Faculty of Engineering and Information Systems, both at the International Burch University. All sample collections in Northern Iraq were carried out through the College of Education-Scientific Department at the University of Salahaddin, which also approved the project, procured the requisite permissions from the local authorities and actively participated in the realization of the project.

Genomic DNA extractions and 17-loci Y-STR haplotyping (DYS19, DYS385a/b, DYS389I/II, DYS390, DYS391, DYS392, DYS393, DYS437, DYS438, DYS439, DYS448, DYS456, DYS458, DYS635 and Y-GATA-H4) were carried out with the Life Technologies PureLink^TM^ Genomic DNA Mini Kit and AmpF*l*STR^®^ Y-filer^TM^ Kit, respectively. Capillary gel electrophoreses were conducted on a Life Technologies ABI 3130 Genetic Analyzer. Alleles were assigned according to the current International Society for Forensic Genetics (ISFG) guidelines for forensic Y-STR analysis [12]. Samples with Y-STR haplotypes bearing bi-allelic patterns at loci other than DYS385a/b were further typed with autosomal STRs (Life Technologies AmpF*l*STR^®^ Identifiler^TM^ Kit) to ascertain their single-source status. All DNA extractions and typing were conducted at the Turkish Cypriot DNA Laboratory as previously described [13, 14]. Y-STR haplotyping and autosomal STR genotyping proficiencies were certified though participation in the YHRD Quality Control Exercise (2013) and ISFG English-Speaking Working Group Relationship Testing Workshop (2015). The following YHRD Accession Numbers were assigned for the five novel Y-STR datasets from the current study: Northern Iraq [Arab]: YA004212; Northern Iraq [Kurdish]: YA004213; Northern Iraq [Syriac]: YA004214; Northern Iraq [Turkmen]: YA004215; and Northern Iraq [Yazidi]: YA004216. All of the five Y-STR datasets are also available at the Figshare online digital repository (https://doi.org/10.6084/m9.figshare.5530510.v1).

Haplotype and allele frequencies were calculated using the direct counting method. Statistical parameters of forensic interest, such as gene diversity (GD) and haplotype diversity (HD) were both calculated according to the Nei’s formula [15]. Analysis of molecular variance (AMOVA) and the subsequent visualization by multi-dimensional scaling (MDS) were carried out using the YHRD online tool [16]. The AMOVA/MDS genetic distance measures were based on Slatkin’s *R*_*st*_ values, significance of which were ascertained with probability (*P*) values (10,000 permutations), which were revised following a Bonferroni correction to account for potential Type I errors [17]. In addition to the five novel Y-STR datasets from the current study, the following datasets from nearby and distant populations and with at least 17-loci Y-STR coverage were also included during AMOVA/MDS analysis (population sample size, YHRD Accession No.): Kuwait City, Kuwait [Arab] (*n* = 285, YA003763), Iraq [Iraqi] (*n* = 124, YA003858), Beirut, Lebanon [Lebanese] (*n* = 555, YA003785 & YA003859), Iran [Iranian] (*n* = 104, YA004237), Cyprus [Turkish Cypriot] (*n* = 380, YA003850), Cyprus [Greek Cypriot] (*n* = 344, YA004186), Cukurova, Turkey [Turk] (*n* = 249, YA003668), Southeastern Anatolia, Turkey [Turkish] (*n* = 150, YA003727 and YA004118), Marmara Region, Turkey [Turkish] (*n* = 385, YA004119), Afghanistan [Pathan] (*n* = 125, YA003701), Russian Federation [Russian] (*n* = 204, YA004184), Ulaanbaatar, Mongolia [Mongolian] (*n* = 261, YA004127), Dhaka, Bangladesh [Bangladeshi] (*n* = 348, YA003445), Beijing, China [Han] (*n* = 847, YA003197, YA003470, YA003861 and YA004160), Albania [Albanian] (*n* = 100, YA003096), Bosnia and Herzegovina [Bosnian] (*n* = 100, YA003787), Marche, Italy [Italian] (*n* = 165, YA003069), Upper Bavaria, Germany [German] (*n* = 200, YA003790), and Tanzania [Tanzanian] (*n* = 101, YA004196). Prior to the AMOVA/MDS analysis, the online YHRD tool removes all haplotypes with (a) null, (b) partial/intermediate alleles (e.g. DYS458*.2), (c) duplicated alleles (except for DYS385), etc. Yet, considering that (a) there are 86 haplotypes with DYS458*.2 in the combined dataset from Northern Iraq (Table 1), and that (b) DYS458*.2-bearing haplotypes are almost exclusively associated with the J1 haplogroup, to ensure the inclusion of the maximum number of haplotypes during AMOVA/MDS, all allelic data at the DYS458 locus was excluded instead (i.e. AMOVA/MDS analysis was carried out with 16-loci Y-STR datasets).

**Table 1:** The number of times each allelic microvariant, bi-allelic pattern and null allele were observed in each of the five ethnic groups from Northern Iraq and their overall frequency in the combined population.

Locus	Variation	Arab (*n* = 102)	Kurdish (*n* = 104)	Syriac (*n* = 86)	Turkmen (*n* = 102)	Yazidi (*n* = 106)	Overall Frequency
**DYS19**	**14,16**	-	-	-	-	1	0.0020
**DYS389I**	**10,13**	1	-	-	-	-	0.0020
	**11,13**	-	-	1	-	-	0.0020
**DYS389II**	**30.3**	-	-	-	-	3	0.0060
	**25,29**	-	-	1	-	-	0.0020
**DYS392**	**Null Allele**	-	-	1	-	1	0.0040
**DYS439**	**8,11**	-	-	1	-	-	0.0020
	**11,12**	1					0.0020
**DYS448**	**Null Allele**	-	-	1	-	1	0.0040
	**16.4**	-	1	-	1	-	0.0040
	**19,20**	-	-	-	-	5	0.0100
	**21,22**	-	-	-	-	3	0.0060
**DYS456**	**Null Allele**	-	-	-	1	-	0.0020
	**12**	1	1	1	-	-	0.0060
	**19**	1	-	-	-	-	0.0020
**DYS458**	**12**	1	-	2	-	-	0.0060
	**17.2**	4	2	3	1	-	0.0200
	**18.2**	24	9	3	6	7	0.0980
	**19.2**	8	5	4	2	-	0.0380
	**20.2**	2	1	-	4	-	0.0140
	**21**	-	1	-	1	-	0.0040
	**21.2**	1	-	-	-	-	0.0020
**Y_GATA_H4**	**14**	-	2	-	1	-	0.0060
**DYS385a/b**	**15.2, 17**	-	2	-	-	-	0.0040

A neighbor-joining (N-J) phylogenetic tree based on the Nei’s discriminant analysis (*D*_*A*_) genetic distance metric and the allele frequencies of each dataset was constructed using the POPTREE2 software [18]. Bootstrap values were calculated based on 10,000 replications. Along with the five novel Y-STR datasets from the current study, the following population datasets with equivalent loci coverages were included during analysis: Cyprus [Greek Cypriot] (*n* = 344) [19]; Cyprus [Greek Cypriot II] (*n* = 574) [20]; Iran [East Iranian] (*n* = 200) and Iran [West Iranian] (*n* = 124) [21]; West Asia [Armenian, Erzurum origin] (*n* = 99), West Asia [Armenian, Hemsheni] (*n* = 89), West Asia [Armenian, Krasnodar] (*n* = 117), West Asia [Armenian, Adygei] (*n* = 49), West Asia [Armenian, Don] (*n* = 92) [22]; Greece [Greek] (*n* = 214), Iraq [Iraqi] (*n* = 124), Barcelona, Spain [Spanish] (*n* = 78), Bohemia, Czechia [Czech] (*n* = 72), Hungary [Hungarian] (*n* = 143), Upper Bavaria, Germany (German) (*n* = 200), Bosnia and Herzegovina [Bosnian] (*n* = 100), Marche, Italy [Italian] (*n* = 170), Sicily, Italy [Italian] (*n* = 157), Central Poland [Polish] (*n* = 102), Central England [English] (*n* = 81), Lebanon [Lebanese] (*n* = 505), Beijing, China [Han] (*n* = 246), Ibadan, Nigeria [Yoruba] (*n* = 81), Kinyawa, Kenya [Maasai] (*n* = 100), Philippines [Filipino] (*n* = 169), Southern India, India [Tamil] (*n* = 126) and Tokyo, Japan [Japanese] (*n* = 59) [23]; Iraq [Iraqi II] (*n* = 400) [24]; Lebanon [Maronite] (*n* = 196) [21]; Cyprus [Turkish Cypriot] (*n* = 380) [13]; Afghanistan [Turkmen] (*n* = 73) [25]; Uzbekistan [Turkmen] (*n* = 83) [26]; Marmara Region, Turkey [Turkish] (*n* = 385) [YHRD Accession No.: YA004119]; Cukurova, Turkey [Turk] (*n* = 249) [27]; and Southeastern Anatolia, Turkey [Turkish] (*n* = 86+64) [28] and [YHRD Accession No.: YA004118].

17-loci Y-STR-based *in silico* haplogroup assignments were made using the 21-haplogroup batch processing version of the Whit Athey algorithm [29]. Validation of the *in silico* haplogroup assignments were carried out using a second algorithm called NevGen Y-DNA Haplogroup Predictor (www.nevgen.org). A stand-alone Python program was implemented, which called the NevGen haplogroup prediction AJAX API directly for each haplotype to allow automated processing of all Y-STR haplotypes. Prior to the NevGen analysis, null alleles, intermediate/partial alleles and multi-allelic patterns (except for DYS385) were each assigned a value of ‘0’.

Median-joining network (M-JN) analyses were carried out using the Network v.5.0.0.1 software (www.fluxus-engineering.com) as previously described [13]. Briefly, (a) all haplotypes with intermediate/partial alleles and/or multi-allelic patterns were removed prior to analysis, (b) a default epsilon parameter value of zero was used, and (c) maximum parsimony post-processing was applied again with the default parameters. Time to the most recent common ancestor (TMRCA) estimates were done on the resultant M-JN trees by selecting a proposed central ancestral node and then all the other nodes in the remaining network as the descendant nodes. Each TMRCA estimate was done in duplicate based on a generation time of 25 years, and the genealogical and evolutionary Y-STR mutation rates of 0.00267 and 0.00069, respectively, both per locus per generation [30–33].

## Results

A combined Y-STR dataset with 500 haplotype from the Northern Iraq populations was generated (S1 Table), wherein there were 360 different and 280 unique haplotypes, hence yielding unique haplotypes (UH) of 56.0% and a discrimination capacity (DC) of 72.0% for the entire dataset. An overall haplotype diversity of 0.9979 was calculated. A number of haplotypes were observed as replicates, often exclusively among a single ethnic group, but a few of these haplotypes were also found to be shared by two different ethnic groups. Tables A-F in S1 File provide allele frequencies and the associated gene diversity (GD) values for the new combined dataset, as well as those for each of the five ethnic groups analyzed.

Table 1 lists the different allelic variants, null alleles and bi-allelic patterns observed among the 500 samples from Northern Iraq: 13 allelic variants at six different loci, eight bi-allelic patterns at five different loci (excluding those at DYS385a/b) and null alleles at three different loci.

Based on the calculated GD values, apart from DYS385a/b, the two most informative loci for the combined dataset are DYS458 (0.8270) and DYS635 (0.7644), while the least informative locus is DYS391 (0.4934) (Table 2). DYS458 also turned out to be the most informative locus for each of the five ethnic groups analyzed.

**Table 2:** Statistical parameters of forensic interest for the combined Northern Iraqi (*n* = 500), as well as the Arab (*n* = 102), Kurdish (*n* = 104), Syriac (*n* = 86), Turkmen (*n* = 102) and Yazidi (*n* = 106) populations.

	Forensic Parameters		Gene Diversities					
Northern Iraqi	Haplotype Diversity (HD)	0.99787	DYS19	0.6345	DYS393	0.5850	DYS458	0.8270
	Average Gene Diversity (GD)	0.6676	DYS389I	0.5964	DYS437	0.5465	DYS635	0.7644
	Number of Samples	500	DYS389II	0.7284	DYS438	0.6979	Y_GATA_H4	0.6582
	Number of Unique Haplotypes	280						
	Unique Haplotypes (UH)	56.00%	DYS390	0.7079	DYS439	0.6746	DYS385a/b	0.9578
	Number of Different Haplotypes	360	DYS391	0.4934	DYS448	0.6588	Av. w/ DYS385a/b	0,6676
	Discrimination Capacity (DC)	72.00%	DYS392	0.5140	DYS456	0.6368	Av. w/o DYS385a/b	0,6482
Arab	Haplotype Diversity (HD)	0.99728	DYS19	0.5590	DYS393	0.5715	DYS458	0.8435
	Average Gene Diversity (GD)	0.6264	DYS389I	0.5609	DYS437	0.3781	DYS635	0.7357
	Number of Samples	102	DYS389II	0.6815	DYS438	0.5863	Y_GATA_H4	0.6261
	Number of Unique Haplotypes	80						
	Unique Haplotypes (UH)	78.43%	DYS390	0.6128	DYS439	0.6592	DYS385a/b	0.9231
	Number of Different Haplotypes	90	DYS391	0.5950	DYS448	0.5598	Av. w/ DYS385a/b	0,6264
	Discrimination Capacity (DC)	88.24%	DYS392	0.4114	DYS456	0.7191	Av. w/o DYS385a/b	0,6067
Kurdish	Haplotype Diversity (HD)	0.99739	DYS19	0.7206	DYS393	0.6344	DYS458	0.8158
	Average Gene Diversity (GD)	0.6499	DYS389I	0.6159	DYS437	0.5264	DYS635	0.7702
	Number of Samples	104	DYS389II	0.7263	DYS438	0.6598	Y_GATA_H4	0.6673
	Number of Unique Haplotypes	84						
	Unique Haplotypes (UH)	80.77%	DYS390	0.7319	DYS439	0.7382	DYS385a/b	0.9523
	Number of Different Haplotypes	93	DYS391	0.4643	DYS448	0.5250	Av. w/ DYS385a/b	0,6499
	Discrimination Capacity (DC)	89.42%	DYS392	0.3251	DYS456	0.5250	Av. w/o DYS385a/b	0,6297
Syriac	Haplotype Diversity (HD)	0.97456	DYS19	0.3794	DYS393	0.3864	DYS458	0.7453
	Average Gene Diversity (GD)	0.5978	DYS389I	0.5725	DYS437	0.5222	DYS635	0.7380
	Number of Samples	86	DYS389II	0.7312	DYS438	0.6920	Y_GATA_H4	0.5687
	Number of Unique Haplotypes	31						
	Unique Haplotypes (UH)	36.05%	DYS390	0.6679	DYS439	0.5552	DYS385a/b	0.8918
	Number of Different Haplotypes	48	DYS391	0.3310	DYS448	0.5592	Av. w/ DYS385a/b	0,5978
	Discrimination Capacity (DC)	55.81%	DYS392	0.6660	DYS456	0.5587	Av. w/o DYS385a/b	0,5782
Turkmen	Haplotype Diversity (HD)	0.99592	DYS19	0.7070	DYS393	0.6034	DYS458	0.7847
	Average Gene Diversity (GD)	0.6691	DYS389I	0.6567	DYS437	0.5073	DYS635	0.7495
	Number of Samples	102	DYS389II	0.7620	DYS438	0.6926	Y_GATA_H4	0.7092
	Number of Unique Haplotypes	74						
	Unique Haplotypes (UH)	72.55%	DYS390	0.7230	DYS439	0.6498	DYS385a/b	0.9516
	Number of Different Haplotypes	86	DYS391	0.5100	DYS448	0.6917	Av. w/ DYS385a/b	0,6692
	Discrimination Capacity (DC)	84.31%	DYS392	0.4060	DYS456	0.6013	Av. w/o DYS385a/b	0,6503
Yazidi	Haplotype Diversity (HD)	0.97952	DYS19	0.5349	DYS393	0.6130	DYS458	0.7916
	Average Gene Diversity (GD)	0.6542	DYS389I	0.4751	DYS437	0.6536	DYS635	0.7581
	Number of Samples	106	DYS389II	0.6849	DYS438	0.6962	Y_GATA_H4	0.5796
	Number of Unique Haplotypes	24						
	Unique Haplotypes (UH)	22.64%	DYS390	0.7330	DYS439	0.6606	DYS385a/b	0.9290
	Number of Different Haplotypes	50	DYS391	0.4466	DYS448	0.6650	Av. w/ DYS385a/b	0,6542
	Discrimination Capacity (DC)	47.17%	DYS392	0.5856	DYS456	0.6598	Av. w/o DYS385a/b	0,6358

Table 3 lists the *R*_st_-based genetic distances and the corresponding *P* values observed among the novel datasets, along with 19 other nearby and distant populations. The closest and farthest genetic distances observed for each novel dataset were as follows: (a) Iraq [Arab] with Kuwait City, Kuwait [Arab] (0.0025) and Ulaanbaatar, Mongolia [Mongolian] (0.2592), (b) Northern Iraq [Kurdish] with Iraq [Iraqi] (0.0046) and Ulaanbaatar, Mongolia [Mongolian] (0.2222), (c) Northern Iraq [Syriac] with Cukurova, Turkey [Turk] (0.0194) and Tanzania [Tanzanian] (0.2984), (d) Northern Iraq [Turkmen] with Iraq [Iraqi] (0.0011) and Ulaanbaatar, Mongolia [Mongolian] (0.2010), and (e) Northern Iraq [Yazidi] with Iran [Iranian] (0.0055) and Afghanistan [Pathan] (0.2054). The closest genetic distance observed among the 24 populations was that in between Iraq [Iraqi] and Iran [Iranian] / Southeastern Anatolia, Turkey [Turkish] (-0.0003 / -0.0005). The corresponding *P* values suggested that the following genetic distances were non-significant: Northern Iraq [Arab] and Kuwait City, Kuwait [Arab]; Northern Iraq [Kurdish] and Northern Iraq [Turkmen]; Northern Iraq [Kurdish] and Iraq [Iraqi]; Northern Iraq [Turkmen] and Cyprus [Turkish Cypriot]; Northern Iraq [Turkmen] and Iraq [Iraqi]; Northern Iraq [Turkmen] and Iran [Iranian]; Northern Iraq [Turkmen] and Beirut, Lebanon [Lebanese]; Northern Iraq [Turkmen] and Southeastern Anatolia, Turkey [Turkish]; Northern Iraq [Yazidi] and Iran [Iranian]; Cyprus [Greek Cypriot] and Cyprus [Turkish Cypriot]; Iran [Iranian] and Iraq [Iraqi]; Iran [Iranian] and Marmara Region, Turkey [Turkish]; Iran [Iranian] and Southeastern Anatolia, Turkey [Turkish]; Southeastern Anatolia, Turkey [Turkish] and Iraq [Iraqi]; Marmara Region, Turkey [Turkish] and Iraq [Iraqi]; Marmara Region, Turkey [Turkish] and Cukurova, Turkey [Turk]; Marmara Region, Turkey [Turkish] and Southeastern Anatolia, Turkey [Turkish]; and Southeastern Anatolia, Turkey [Turkish] and Cukurova, Turkey [Turk]. Upon the Bonferroni correction, the following population pairs were also found to have non-significant differences (a) Northern Iraq [Yazidi] with each of the other four populations from the current study, (b) Northern Iraq [Arab] and Northern Iraq [Kurdish], (c) Northern Iraq [Arab] and Northern Iraq [Turkmen], and (d) numerous others that are also geographically and/or historically connected.

**Table 3:** Pairwise genetic distance matrix based on the *R*_*st*_ and *P* values between the five major ethnic groups from Northern Iraq and representative nearby and distant populations†.

	Population	1	2	3	4	5	6	7	8	9	10	11	12	13	14	15	16	17	18	19	20	21	22	23	24
**1**	Northern Iraq [Arab]*	-	*0*.*0472*	0.0000	*0*.*0019*	*0*.*0019*	0.0000	0.0000	*0*.*0002*	0.0000	0.0000	0.0000	0.0000	0.0000	*0*.*0063*	*0*.*0091*	0.0000	**0.2979**	*0*.*0043*	0.0000	0.0000	0.0000	0.0000	0.0000	*0*.*0009*
**2**	Northern Iraq [Kurdish]*	0.0137	-	0.0000	**0.0898**	*0*.*0015*	0.0000	*0*.*0006*	*0*.*0117*	0.0000	0.0000	*0*.*0016*	*0*.*0041*	0.0000	*0*.*0394*	**0.1695**	0.0000	*0*.*0100*	*0*.*0206*	0.0000	0.0000	0.0000	0.0001	*0*.*0004*	*0*.*0052*
**3**	Northern Iraq [Syriac]*	0.1005	0.1025	-	0.0001	*0*.*0013*	0.0000	0.0000	0.0000	0.0000	0.0000	0.0000	0.0000	0.0000	*0*.*0015*	*0*.*0004*	*0*.*0011*	0.0000	0.0000	0.0000	0.0000	0.0000	*0*.*0082*	*0*.*0002*	*0*.*0012*
**4**	Northern Iraq [Turkmen]*	0.0344	0.0091	0.0762	-	*0*.*0215*	0.0000	*0*.*0105*	*0*.*0004*	*0*.*0003*	0.0000	*0*.*0401*	**0.1629**	0.0000	**0.1004**	**0.2998**	*0*.*0003*	0.0000	**0.0607**	0.0000	0.0000	0.0000	*0*.*0006*	*0*.*0279*	**0.0736**
**5**	Northern Iraq [Yazidi]*	0.0369	0.0394	0.0545	0.0205	-	0.0000	*0*.*0136*	0.0000	0.0000	0.0000	*0*.*0026*	*0*.*0040*	0.0000	**0.1619**	*0*.*0114*	*0*.*0011*	0.0000	*0*.*0146*	0.0000	0.0000	0.0000	0.0001	*0*.*0011*	*0*.*0041*
**6**	**Afghanistan [Pathan]**	0.2257	0.1966	0.1162	0.1999	0.2054	-	0.0000	0.0000	0.0000	0.0000	0.0000	0.0000	0.0000	0.0000	0.0000	0.0000	0.0000	0.0000	0.0000	0.0000	0.0000	0.0000	0.0000	0.0000
**7**	**Albania [Albanian]**	0.0892	0.0560	0.1004	0.0314	0.0289	0.2313	-	0.0000	*0*.*0079*	0.0000	*0*.*0007*	*0*.*0005*	0.0000	*0*.*0089*	*0*.*0051*	*0*.*0022*	0.0000	0.0000	0.0000	0.0000	0.0000	0.0000	0.0000	*0*.*0003*
**8**	**Dhaka, Bangladesh [Bangladeshi]**	0.0416	0.0157	0.0971	0.0342	0.0605	0.1460	0.0765	-	0.0000	0.0000	0.0000	0.0000	0.0000	*0*.*0008*	*0*.*0032*	0.0000	0.0000	0.0000	0.0000	0.0000	0.0000	0.0000	0.0000	0.0000
**9**	**Bosnia and Herzegovina [Bosnian]**	0.1604	0.0878	0.1925	0.0622	0.0955	0.2636	0.0355	0.0800	-	0.0000	0.0000	0.0000	0.0000	0.0000	0.0000	0.0000	0.0000	0.0000	0.0000	0.0000	0.0000	0.0000	0.0000	0.0000
**10**	**Beijing, China [Han]**	0.0898	0.1023	0.0836	0.1220	0.0882	0.1304	0.1410	0.1012	0.1880	-	0.0000	0.0000	0.0000	0.0000	0.0000	0.0000	0.0000	0.0000	0.0000	0.0000	0.0000	0.0000	0.0000	0.0000
**11**	**Cyprus [Greek Cypriot]**	0.0493	0.0259	0.1188	0.0097	0.0238	0.2778	0.0321	0.0686	0.0721	0.1471	-	**0.2884**	0.0000	*0*.*0003*	*0*.*0006*	0.0000	0.0000	*0*.*0018*	0.0000	0.0000	0.0000	0.0000	0.0000	0.0001
**12**	**Cyprus [Turkish Cypriot]**	0.0428	0.0181	0.0960	0.0031	0.0185	0.2384	0.0325	0.0521	0.0651	0.1307	0.0004	-	0.0000	*0*.*0028*	*0*.*0053*	0.0000	0.0000	*0*.*0040*	0.0000	0.0000	0.0000	0.0000	0.0000	0.0001
**13**	**Upper Bavaria, Germany [German]**	0.1965	0.1575	0.0798	0.1247	0.1212	0.0934	0.1056	0.1141	0.1358	0.1455	0.1793	0.1513	-	0.0000	0.0000	0.0001	0.0000	0.0000	0.0000	0.0001	0.0000	0.0000	0.0000	0.0000
**14**	**Iran [Iranian]**	0.0266	0.0142	0.0385	0.0083	0.0055	0.1519	0.0321	0.0292	0.0865	0.0668	0.0305	0.0192	0.0944	-	**0.4129**	*0*.*0009*	0.0000	*0*.*0485*	0.0000	0.0000	0.0000	*0*.*0306*	**0.1365**	**0.1714**
**15**	**Iraq [Iraqi]**	0.0259	0.0046	0.0506	0.0011	0.0233	0.1509	0.0337	0.0183	0.0653	0.0899	0.0252	0.0148	0.0967	-0.0005	-	0.0001	0.0000	*0*.*0275*	0.0000	0.0000	0.0000	*0*.*0226*	**0.1719**	**0.4068**
**16**	**Marche, Italy [Italian]**	0.1145	0.0855	0.0424	0.0431	0.0392	0.1614	0.0378	0.0872	0.0948	0.1250	0.0721	0.0559	0.0430	0.0325	0.0391	-	0.0000	0.0000	0.0000	0.0000	0.0000	*0*.*0005*	*0*.*0002*	0.0001
**17**	**Kuwait City, Kuwait [Arab]**	0.0011	0.0148	0.1285	0.0352	0.0577	0.2616	0.1055	0.0545	0.1586	0.1193	0.0446	0.0415	0.2249	0.0443	0.0365	0.1298	-	0.0000	0.0000	0.0000	0.0000	0.0000	0.0000	0.0000
**18**	**Beirut, Lebanon [Lebanese]**	0.0182	0.0114	0.0718	0.0065	0.0124	0.2222	0.0467	0.0457	0.0940	0.1082	0.0099	0.0070	0.1547	0.0075	0.0087	0.0636	0.0237	-	0.0000	0.0000	0.0000	0.0000	0.0000	*0*.*0014*
**19**	**Ulaanbaatar, Mongolia [Mongolian]**	0.2592	0.2222	0.1434	0.2010	0.2042	0.0986	0.1973	0.1633	0.2045	0.2004	0.2652	0.2318	0.0618	0.1724	0.1610	0.1454	0.3023	0.2349	-	*0*.*0002*	0.0000	0.0000	0.0000	0.0000
**20**	**Russian Federation [Russian]**	0.2563	0.2089	0.1399	0.1801	0.1938	0.0827	0.1736	0.1414	0.1775	0.1872	0.2451	0.2104	0.0257	0.1540	0.1423	0.1146	0.2850	0.2126	0.0221	-	0.0000	0.0000	0.0000	0.0000
**21**	**Tanzania [Tanzanian]**	0.1707	0.1842	0.2984	0.1833	0.1768	0.4160	0.2158	0.2467	0.2774	0.2969	0.2070	0.2026	0.3675	0.2108	0.2169	0.2677	0.1903	0.2302	0.4383	0.4327	-	0.0000	0.0000	0.0000
**22**	**Çukurova, Turkey [Turk]**	0.0662	0.0462	0.0194	0.0266	0.0385	0.1087	0.0658	0.0441	0.1022	0.0915	0.0671	0.0480	0.0615	0.0102	0.0105	0.0231	0.0826	0.0378	0.1316	0.1052	0.2928	-	**0.1206**	**0.0768**
**23**	**Marmara Region, Turkey [Turkish]**	0.0578	0.0310	0.0364	0.0107	0.0259	0.1382	0.0417	0.0347	0.0658	0.0983	0.0407	0.0270	0.0696	0.0039	0.0024	0.0220	0.0698	0.0233	0.1429	0.1137	0.2785	0.0023	-	**0.1085**
**24**	**Southeastern Anatolia, Turkey [Turkish]**	0.0354	0.0237	0.0397	0.0086	0.0261	0.1556	0.0551	0.0384	0.1002	0.0952	0.0390	0.0266	0.0983	0.0040	-0.0003	0.0357	0.0485	0.0160	0.1677	0.1473	0.2402	0.0050	0.0036	-

† *P* values are shown above and the *R*_*st*_ values below the diagonal; the level of significance is *p*<0.05 and *p*<0.0002 (0.05/276; 276 is the number of pairwise comparisons) before and after the Bonferroni correction, respectively.

* Denotes datasets from the current study. *P* values in bold and italics represent statistically non-significant differences before and after the Bonferroni correction, respectively.

**Fig 2:**
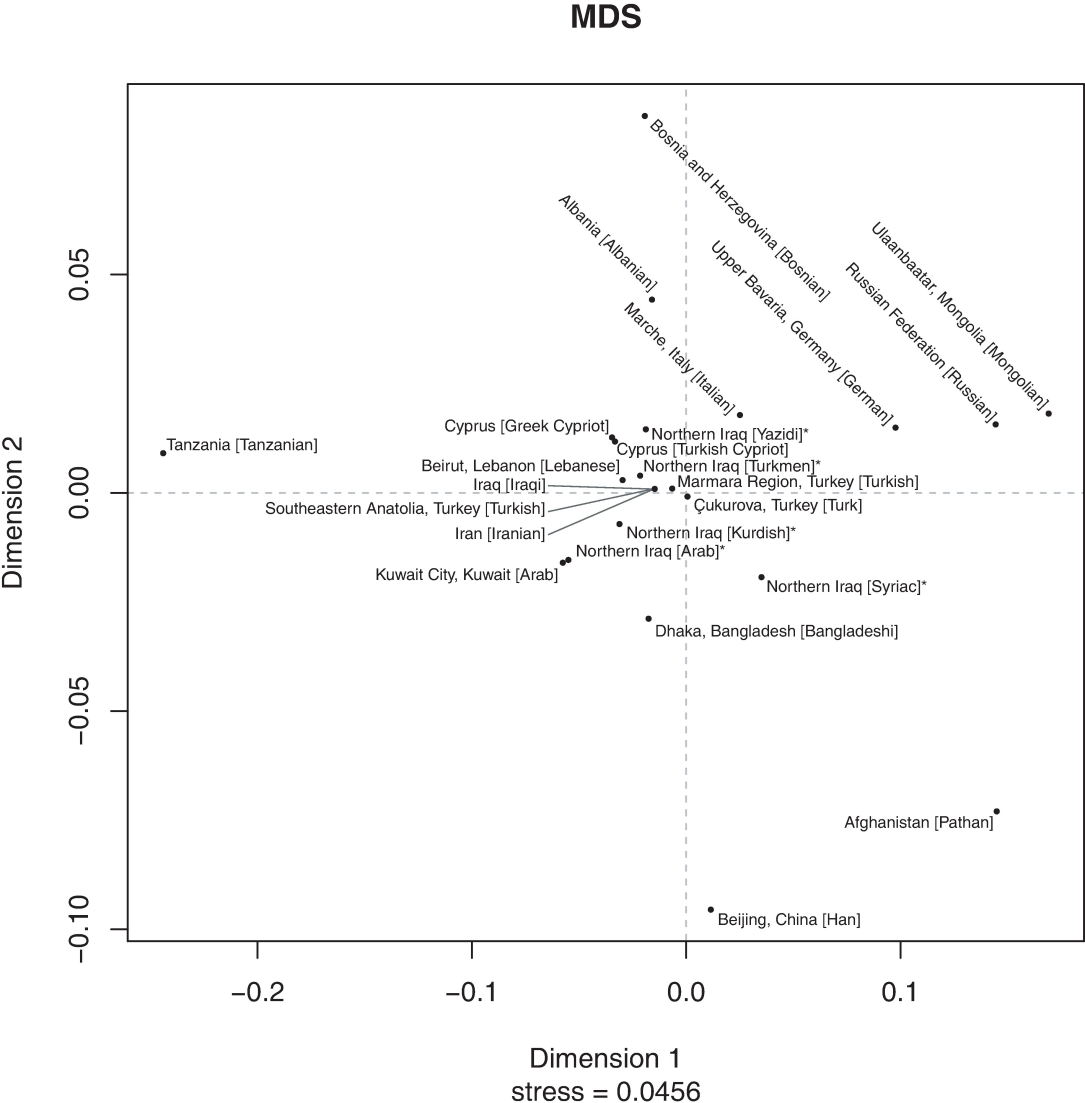
A two-dimensional MDS plot showing genetic relationships among the five Northern Iraqi ethnic groups and representative nearby and distant populations. Asterisks (*) mark populations from the present study.

A two-dimensional MDS plot based on the *R*_st_-values suggested (a) a core cluster compising the Iraq [Iraqi]; Iran [Iranian]; Southeastern Anatolia, Turkey [Turkish]; Marmara Region, Turkey [Turkish]; Cukurova, Turkey [Turk]; Beirut, Lebanon [Lebanese] and Northern Iraq [Turkmen] population datasets, immediately surrounded by the Northern Iraq [Kurdish]; Northern Iraq [Yazidi], Cyprus [Turkish Cypriot] and Cyprus [Greek Cypriot] population datasets (b) the five novel population datasets from Northern Iraq differentiated from each other at least in one dimension (Northern Iraq [Kurdish], Northern Iraq [Turkmen] and Northern Iraq [Yazidi]) or in both dimensions (Northern Iraq [Arab] and Northern Iraq [Syriac]) (c) Northern Iraq [Arab] and Kuwait City, Kuwait [Arab] clustered closely together, but less so with the core cluster, (d) Iraq [Iraqi], Iran [Iranian] and Southeastern Anatolia, Turkey [Turkish] clustered very closely, and in fact on top of each other in two dimensions, and (e) Asian, African and European population datasets differentiated in both dimensions from the core cluster, but respective population datasets clustered among themselves as expected (Fig 2).

**Fig 3:**
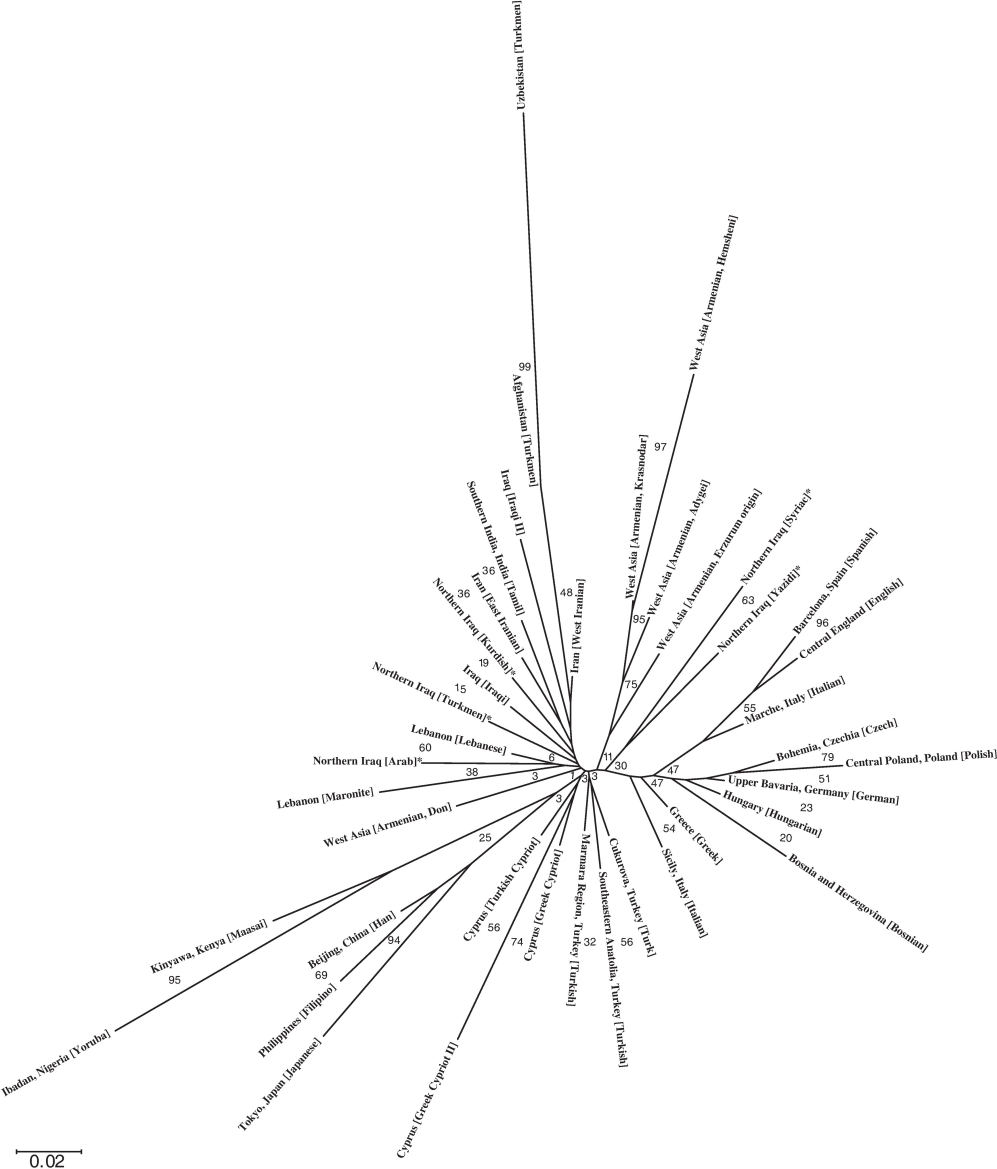
An N-J phylogenetic tree showing genetic distances among the 40 nearby and distant populations. Asterisks (*) mark populations from the present study. Numerical assignments at each node denote the calculated bootstrap value at that node. A scale bar corresponding to the phylogenetic tree branch lengths is also provided.

To provide an alternative view on the genetic affinities among the five different ethnic datasets from the current study, a phylogenetic tree was also constructed based on Nei’s *D*_*A*_ genetic distance metric and in the context of a even wider genetic landscape (S2 Table and Fig 3). Results from this second approach suggested that (a) Northern Iraq [Arab] clustered most closely with Lebanon [Lebanese] and Lebanon [Maronite]; Northern Iraq [Kurdish] clustered most closely with Iraq [Iraqi] and Iran [East Iranian]; and at the next level, Northern Iraq [Turkmen] grouped in between Northern Iraq [Arab] and Northern Iraq [Kurdish] clusters, and (b) Northern Iraq [Syriac] and Northern Iraq [Yazidi] clustered together, but away from the other Northern Iraqi populations analyzed in the current study, and largely in between the West Asian and Southeastern European populations. As a testament to overall validity of the phylogenetic tree constructed, (a) Turkish populations from Marmara, Southeastern Anatolia and Cukurova, (b) Cypriot populations (Turkish Cypriot, Greek Cypriot and Greek Cypriot II), (c) four out of the five Armenian populations analyzed (Krasnodar, Hemsheni, Adygei and Erzurum Origin), (d) Turkmen populations from Central/South Asia (Afghanistan and Uzbekistan), and (e) African, Southeast Asian and European populations were all found to cluster most closely among their respective populations.

S3 Table lists the individual ‘fitness scores’ and ‘Bayesian probabilities’ for the *in silico* haplogroup assignment for each sample by two different algorithms used in the current study. Notably, 96.8% of the *in silico* haplogroup assignments by the Whit Athey algorithm had ‘fitness scores’ and ‘Bayesian probabilities’ above the set thresholds, which were 25 and 50%, respectively. There were no particular trends for the ambiguous haplogroup assignments, i.e. those with the associated fitness score and/or Bayesian probability below the set threshold for this algorithm. A comparison of the *in silico* haplogroup assignments made by the two different algorithms suggested a ‘gross discrepancy rate’ of 10.2% (51 discrepancies out of a total of 500 assignments) and a ‘corrected discrepancy rate’ of only 5.8% (28 discrepancies out of 484 assignments). The ‘corrected discrepancy rate’ reflects a more accurate picture, because (a) out of a total of 500 haplogroup assignments made by the Whit Athey algorithm, only 484 were assumed to be unambiguous, and hence processed any further (S3 Table), and (b) out of the 51 discrepancies observed between the 500 haplogroups assignments made by the two algorithms tested, only 28 of them corresponded to full discrepancies with the 484 unambigious haplogroup assignments by the White Athey method, while the rest corresponded to discrepancies at only the sub-clade level (e.g. J2a1 versus J2a2, etc.).

**Fig 4:**
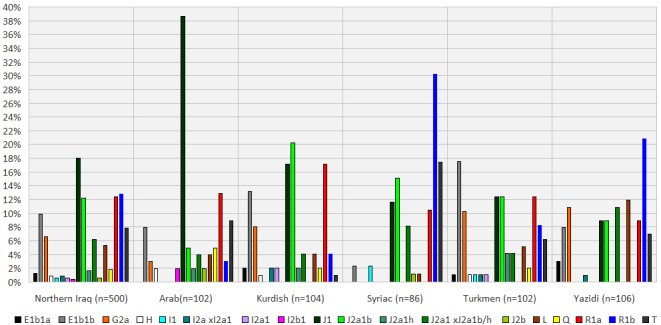
Distribution of the *in silico* assigned (by the Whit Athey algorithm) Y-chromosomal haplogroups for the five different ethnic groups from Northern Iraq, as well as for the combined dataset (*n* = 500).

Table 4 and Fig 4 show distributions of the haplogroup assignments for the combined dataset from Northern Iraq, as well as for each of the five different ethnic groups therein. 18 out of the 21 possible haplogroup assignments that could be made were observed in the combined dataset, hence pointing out to the high heterogeneity of the Northern Iraqi populations. However, it must be noted that without proper haplogroup assignments by Y-SNP typing, such *in silico* haplogroup assignments should be treated solely as preliminary findings since being based on Y-STR data alone, they may not always be accurate [34]. In other words, caution should always be exercised when making relevant conclusions based on such *in silico* produced data alone.

**Table 4:** Distribution of the *in silico* assigned Y-chromosomal haplogroups by the Whit Athey algorithm for the 17-loci Y-STR datasets from Northern Iraq (*n* = 500).

*Haplogroup*	E1b1a	E1b1b	G2a	H	I1	I2a xI2a1	I2a1	I2b1	J1	J2a1b	J2a1h	J2a1 xJ2a1b/h	J2b	L	Q	R1a	R1b	T	*Total* *Unambigous* *Assignments*
Combined (*n* = 500)																			
Total observed	6	48	32	4	3	4	3	2	87	59	8	30	3	26	9	60	62	38	484
% observed	**1.24%**	**9.92%**	**6.61%**	**0.83%**	**0.62%**	**0.83%**	**0.62%**	**0.41%**	**17.98%**	**12.19%**	**1.65%**	**6.20%**	**0.62%**	**5.37%**	**1.86%**	**12.40%**	**12.81%**	**7.85%**	
Arab (*n* = 102)																			
Total observed	0	8	3	2	0	0	0	2	39	5	2	4	2	4	5	13	3	9	101
% observed	**0.00%**	**7.92%**	**2.97%**	**1.98%**	**0.00%**	**0.00%**	**0.00%**	**1.98%**	**38.61%**	**4.95%**	**1.98%**	**3.96%**	**1.98%**	**3.96%**	**4.95%**	**12.87%**	**2.97%**	**8.91%**	
Kurdish (*n* = 104)																			
Total observed	2	13	8	1	0	2	2	0	17	20	2	4	0	4	2	17	4	1	99
% observed	**2.02%**	**13.13%**	**8.08%**	**1.01%**	**0.00%**	**2.02%**	**2.02%**	**0.00%**	**17.17%**	**20.20%**	**2.02%**	**4.04%**	**0.00%**	**4.04%**	**2.02%**	**17.17%**	**4.04%**	**1.01%**	
Syriac (*n* = 86)																			
Total observed	0	2	0	0	2	0	0	0	10	13	0	7	1	1	0	9	26	15	86
% observed	**0.00%**	**2.33%**	**0.00%**	**0.00%**	**2.33%**	**0.00%**	**0.00%**	**0.00%**	**11.63%**	**15.12%**	**0.00%**	**8.14%**	**1.16%**	**1.16%**	**0.00%**	**10.47%**	**30.23%**	**17.44%**	
Turkmen (*n* = 102)																			
Total observed	1	17	10	1	1	1	1	0	12	12	4	4	0	5	2	12	8	6	97
% observed	**1.03%**	**17.53%**	**10.31%**	**1.03%**	**1.03%**	**1.03%**	**1.03%**	**0.00%**	**12.37%**	**12.37%**	**4.12%**	**4.12%**	**0.00%**	**5.15%**	**2.06%**	**12.37%**	**8.25%**	**6.19%**	
Yazidi (*n* = 106)																			
Total observed	3	8	11	0	0	1	0	0	9	9	0	11	0	12	0	9	21	7	101
% observed	**2.97%**	**7.92%**	**10.89%**	**0.00%**	**0.00%**	**0.99%**	**0.00%**	**0.00%**	**8.91%**	**8.91%**	**0.00%**	**10.89%**	**0.00%**	**11.88%**	**0.00%**	**8.91%**	**20.79%**	**6.93%**	

While the most prevalent four lineages observed in the combined dataset were J1 (17.98%), R1b (12.81%), R1a (12.40%) and J2a1b (12.19%), the distributions among the five ethnic groups were found to vary significantly: (a) 14 different haplogroups were observed in Arabs, with the three most common being J1 (38.61%), R1a (12.87%) and T (8.91%), (b) 15 different haplogroups were observed in Kurds, with the three most common being J2a1b (20.20%), J1 / R1a (17.17%) and E1b1b (13.13%), (c) 10 different haplogroups were observed in Syriacs, with the three most common being R1b (30.23%), T (17.44%) and J2a1b (15.12%), (d) 16 different haplogroups were observed in Turkmens, with the three most common being E1b1b (17.53%), J1 / J2a1b / R1a (12.37%) and G2a (10.31%) and (e) 11 different haplogroups were observed in Yazidis, with the three most common being R1b (20.79%), L (11.88%) and G2a / J2a1x J2a1b/h (10.89%).

**Fig 5:**
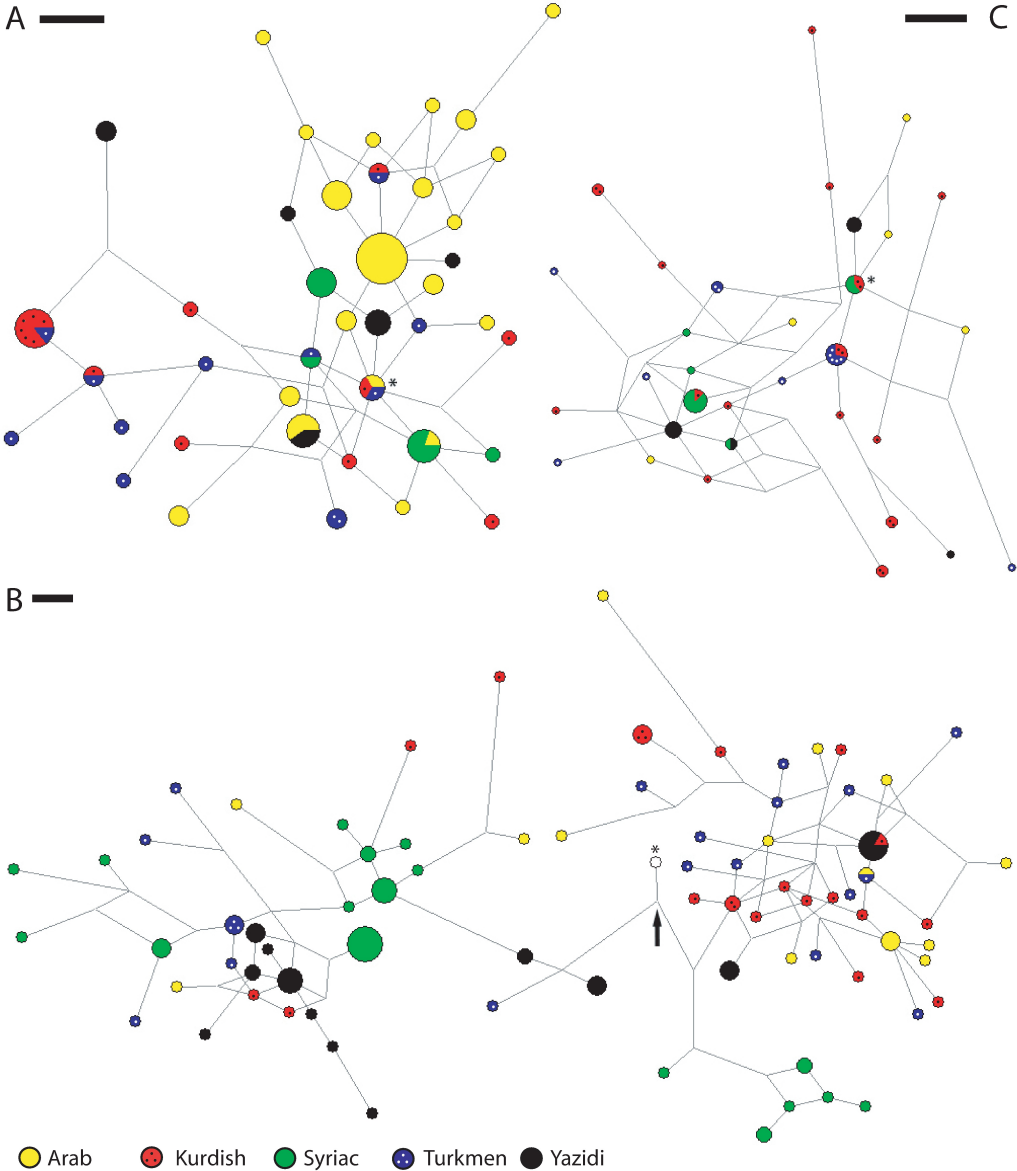
**Counter-clockwise: Panel A, J1 M-JN based on eight Y-STR loci** (excluded loci are DYS385a/b, DYS389I/II, DYS392, DYS437, DYS438, DYS448 and DYS58); **Panel B, the combined R1a and R1b M-JN based on 13 Y-STR loci** (excluded loci are DYS385a/b and DYS389I/II), the R1a and R1b networks are in fact split along the right and left of the black arrow, respectively, and just below the proposed ancestral modal haplotype for both haplogroups, which was not sampled; **Panel C, J2a1b M-JN based on eight Y-STR loci** (excluded loci are DYS385a/b, DYS389I/II, DYS392, DYS437, DYS438, DYS448 and DYS58). Asterisks (*) mark the proposed ancestral modal haplotypes. A scale bar whose length denotes a single mutation event between two neighbouring haplotypes is also provided for each network.

Fig 5 depicts M-JN analyses for the four most prevalent Y-chromosomal haplogroups observed in the combined dataset, namely J1, R1b, R1a and J2a1b. The proposed ancestral modal haplotypes for these three networks comprised samples from the following ethnic groups: (a) Arab / Kurdish / Turkmen for J1, (b) an unknown ancestor for R1a/R1b, which was closest to two Yazidi haplotypes from R1b and a Kurdish haplotype for R1a, and (c) Syriac / Kurdish for J2a1b. The following TMRCA estimates were made using both the genealogical and evolutionary Y-STR mutation rates (estimates in brackets are given in the same order): J1 (3782±825 and 14640±3193 years), R1a (6309±1610 and 24422±6230 years), R1b (9314±2214 and 36051±8571 years) and J2a1b (4006±907 and 15506±3513 years). TMRCA estimates were also made for the microvariations among the DYS448*19,20-bearing haplotypes exclusively observed in Yazidis. Briefly, this bi-allelic pattern was observed in four different 17-loci Y-STR haplotypes with the following allelic variations: Yz-M-058 to Yz-M-056/Yz-M-57 by a single-step mutation at DYS439 (11 to 12): Yz-M-056/Yz-M-57 to Yz-M-037 by a single-step mutation at DYS19 (15 to 14); Yz-M-037 to Yz-M-040 by a single-step mutation at DYSS89II (29 to 30) or *vice versa*. Since the ancestral haplotype could not reliably be determined with the available data, four different sets of TMRCA estimates were made with each of the genealogical and evolutionary Y-STR mutation rates, where the DYS448 locus was invariably excluded due to the bi-allelic pattern, and suggested a time-scale of 468±287 to 936±597 years and 1811±1109 to 3622±2309 years, respectively.

## Discussion

HD values ranging between 0.97456 and 0.99739 were observed for the Syriac and Kurdish population datasets, respectively, and intermediate values for the remaining three ethnic groups analyzed (Table 2). An immediate difference between the 17-loci Y-STR datasets obtained was that in the number of haplotype replicates observed, both at intra and inter population levels, and as reflected by the UH values observed: Arabs (78.43%), Kurds (80.77%), Syriacs (36.05%), Turkmens (72.55%) and Yazidis (22.64%). Such low UH values observed for the Syriac and Yazidi ethnic groups are perhaps reflective of the well-documented isolation and/or strict, religious endogamy in these communities [7, 35]. The observed DC values for each population dataset also exhibited significant variations, ranging from 47.17% for Yazidis to 89.42% for Kurds and intermediate values for the other three ethnicities (Table 2). A somewhat counteracting effect was the observation of numerous rare genetic variations that could potentially help during forensic investigations and may also provide novel insights from an anthropological perspective (Table 1).

Although based on two different genetic distance metrics, namely *R*_st_ and Nei’s *D*_*A*_, and also analyses comprising largely different population datasets, AMOVA/MDS (Table 3 and Fig 2) and N-J phylogenetic tree (S2 Table and Fig 3) analyses seemingly revealed concordant results whereby each of the new population datasets from the current study were found to be distinct in the sense that they all exhibited differential clustering with each other and those from other nearby/distant populations.

To provide further insights from an anthropological perspective, haplogroup assignments were made with the popular Whit Athey haplogroup assignment algorithm, the results of which were then further validated through the use of a second algorithm, namely the NevGen Y-DNA Haplogroup Predictor (S3 Table). Observation of a ‘gross discrepancy rate’ of 10.2% and a ‘corrected discrepancy rate’ of only 5.8% suggested that such *in silico* haplogroup assignment tools could perhaps provide some insights when proper Y-SNP data is not available. So, with great caution, the following relevant conclusions were made based on such *in silico* produced data alone. The R (25%) and J (39%) macrohaplogroups were found to account for over 60% in total for the combined dataset from Northern Iraq, which is consistent with the fact that both macrohaplogroups are thought to originate from the Near East as pre-Last Glacial Maximum events that subsequently spread to Europe during late Mesolithic and early Neolithic time, respectively (Table 4 and Fig 4) [36, 37]. In contrast, significant variations were observed in the actual distribution of specific sub-clades of these and other macrohaplogroups among the five different ethnic groups from Northern Iraq, perhaps akin to other highly admixed and/or divergent populations from the Near East [13, 37–39]. While there are a number of earlier studies on the paternal lineages of various Kurdish populations, these correspond to smaller population samples and/or loci coverages than that in the current study [39–43]. One of these earlier studies included Y-SNP-based haplogroups distribution for four Kurdish populations in total from Turkey, Georgia and Turkmenistan, where J2 and R were observed up to 32% and 37%, respectively [42]. In a more recent study focusing on different ethnic groups from Iran, haplogroups J2 and R were both observed at 24% in Kurds, wherein R1a alone accounted for 20% [39]. Consequently, results from these earlier studies are in good agreement with those for Northern Iraqi Kurds from the current study, wherein J2 subclades were found to account for 22%, while lineages R1a and R1b together accounted for 21%, and with R1a at 17%. Y-chromosomal data on various Arabic-speaking populations across a wide geography ranging from North Africa to West Asia are also available in the literature, often pointing out to the heterogeneous nature of these populations and reflective of their panethnic composition. Y-chromosomal haplogroup distributions in Marsh Arabs from the eastern part of Iraq were also investigated, wherein J1 was found to be the most prevalent lineage with its three markers accounting for 81% in total [44]. Hence, results from the current study on the Northern Iraqi Arabs are in good agreement with those for Marsh Arabs because J1 lineages accounted for around 39% in the former, constituting the highest not only in this ethnic group, but also among all five analyzed. Considering that J1 is thought to originate from a geographical zone that includes northeastern Syria, northern Iraq and eastern Turkey, from where it expanded to the rest of the Near East and North Africa, such high prevalence of J1 among Iraqi Arabs is indicative of their indigenous nature [45]. There are also a number of earlier investigations on the paternal lineages of various Turkmen populations [25, 26, 39, 46]. However, a distinction should perhaps be made between the Turkic populations from Turkmenistan in Central Asia and elsewhere, such as in Northern Iraq and Northern Syria. At least the Northern Iraqi Turkmen, although still Turkic and thus with historical links with Central Asia, have even closer links with the Turkic populations from Anatolia and/or Azerbaijan/Northwestern Iran. Earlier investigations on the Turkmen population in Afghanistan, Uzbekistan and Iran, suggested that haplogroup Q was the most prevalent accounting for 34%, 73% and 43%, in that order [25, 26, 39]. An earlier study from the Turkmenistan population *per se* also exists, albeit of relatively poor Y-SNP typing resolution, whereby the most prevalent haplogroups observed were P(xR1a), J and N(x3) with the frequencies of 52%, 24% and 10%, in that order [46]. Results from the current study suggest that haplogroup distribution for the Northern Iraqi Turkmen population is more similar to that of other Northern Iraqi populations, such as Kurds, as well as Turkish populations in Southeastern Anatolia and Cyprus [13, 37]. Results from the current study also suggested that, the paternal lineages of the Northern Iraqi Syriacs are rather homogenous, and exhibit signs of a strong population bottleneck, a situation perhaps even further emphasized due to strict endogamy known to be practiced in this ethnic group (Table 2). This also seems to be the case for the Northern Iraqi Yazidis, where strict endogamy is also practiced in a relatively small and isolated population of around half a million people [7, 47]. In the case of Northern Iraqi Syriacs, significant *R*_*st*_ genetic distances were observed with all other nearby populations, except for the Yazidis from the current study, and Iraqis, Iranians, Italian (Marche) and Turkish populations from Cukurova, the Marmara Region and Southeastern Anatolia in general (Table 3, Fig 2). In contrast, the Northern Iraqi Yazidis were found to have non-significant *R*_*st*_ genetic distances with all other four ethnic groups from the current study, as well as those from Albania, Cyprus, Iraq, Iran Lebanon and Italy (Marche), as well as the Turkish populations from the Marmara Region and Southeastern Anatolia (Table 3, Fig 2). Consequently, despite corresponding to isolated and homogenous populations, contemporary Syriacs and Yazidis from Northern Iraq may in fact have a stronger continuity with the original genetic stock of the Mesopotamian people, which possibly provided the basis for the ethnogenesis of various subsequent Near Eastern populations. Such an observation seems to be in line with genetic distance calculations based on a different method, namely Nei’s *D*_*A*_ genetic distance, whereby the Northern Iraqi Syriac and Yazidi populations from the current study were found to position in the middle of a genetic continuum between the Near East and Southeastern Europe. Earlier Y-chromosomal haplogroup distribution data on Syriacs from Northern Iraq (*n* = 7) and Iran (*n* = 48 and 55) suggested an overall dominance by the R and J haplogroups [35, 39, 45]. In particular, in the most recent study with the highest haplogroup resolution (*n* = 48), R1a, R1b, J1 and J2 sub-clades were found to account for 8%, 29%, 15% and 15% in that order among Assyrians from Iran [39]. In this respect, the results from the current study, albeit on Northern Iraqi Syriacs (*n* = 86) are in good agreement because J and R subclades were observed at 36% and 41%, respectively, where R1a, R1b, J1 and J2 sub-clades accounted for 11%, 30%, 12% and 24%. Unfortunately no previously published data exists on the Y-chromosomal haplogroup distributions in Yazidis from Northern Iraq or elsewhere, hence precluding comparisons with those from the current study. Results from the current study suggest dominance by R haplogroup subclades among Yazidis, where R1a and R1b account for 9% and 21%, respectively. M-JN and associated TMRCA analyses on haplotypes with J1, J2a1b, R1a and R1b haplogroup assignments among Northern Iraqis all suggested *in situ* radiation as a plausible model to explain the diversity of the corresponding paternal lineages. This is because there were seemingly: (a) a number of star-like descent clusters in the J1 network, exclusively or partially comprised of Arab haplotypes, which dominated the overall network, (b) two star-like descent clusters in the R1b network, one comprising Syriac and the other Yazidi haplotypes, which also both dominated the overall network, and (c) two star-like descent clusters in the J2a1b network, one comprising Syriac / Kurdish and the other Yazidi haplotypes, although the overall network was dominated by Kurdish haplotypes.

In conclusion, data presented herein constitutes a significant primer for further population studies and forensic investigations in Northern Iraq, such as the missing person identification efforts due to past and present conflicts. Novel insights into the molecular anthropology of Near Eastern populations are also expected due to hitherto scantity of genetic data from this corner of the world of immense historical importance. However, it should be noted that the major limitation to this study is the lack of Y-SNP genotyping.

## Supporting information

S1 Table 17-loci Y-STR haplotypes observed in the Northern Iraqi populations (*n* = 500). (DOCX)

S2 Table Pairwise genetic distance matrix based on Nei's *D*_*A*_ values between the five major ethnic groups from Northern Iraq and representative nearby and distant populations. (XLS)

S3 Table *In silico* Y-chromosomal haplogroup assignments for the Northern Iraqi samples by the Whit Athey 21-haplogroup prediction and the NevGen Y-DNA haplogroup predictor algorithms (*n* = 500). (DOC)

S1 File Table A: Allele frequencies of the 17 Y-STR loci for the combined Northern Iraqi population (*n* = 500). Table B: Allele frequencies of the 17 Y-STR loci for the Northern Iraq Arab population (*n* = 102). Table C: Allele frequencies of the 17 Y-STR loci for the Northern Iraq Kurdish population (*n* = 104). Table D: Allele frequencies of the 17 Y-STR loci for the Northern Iraq Syriac population (*n* = 86). Table E Allele frequencies of the 17 Y-STR loci for the Northern Iraq Turkmen population (*n* = 102). Table F: Allele frequencies of the 17 Y-STR loci for the Northern Iraq Yazidi population (*n* = 106).
(DOCX)
