# Supplementary material for: A glimpse at the intricate mosaic of ethnicities from Mesopotamia: Paternal lineages of the Northern Iraqi Arabs, Kurds, Syriacs, Turkmens and Yazidis
Source: PLoS One. 2017 Nov 3;12(11):e0187408. doi: 10.1371/journal.pone.0187408 (PMC5669434; doi:10.1371/journal.pone.0187408)
Supplement: S1 Table — (DOCX) [file pone.0187408.s001.docx]

**S1 Table**. 17-loci Y-STR haplotypes observed in the Northern Iraqi populations (*n*=500).

| **Haplotype ID** | **DYS456** | **DYS389I** | **DYS390** | **DYS389II** | **DYS458** | **DYS19** | **DYS385a/b** | **DYS393** | **DYS391** | **DYS439** | **DYS635** | **DYS392** | **Y_GATA_H4** | **DYS437** | **DYS438** | **DYS448** |
| --- | --- | --- | --- | --- | --- | --- | --- | --- | --- | --- | --- | --- | --- | --- | --- | --- |
| Ar-M-001 | 17 | 13 | 24 | 31 | 16 | 14 | 17,18 | 13 | 10 | 12 | 21 | 11 | 11 | 14 | 10 | 20 |
| Ar-M-002 | 15 | 13 | 23 | 29 | 16 | 14 | 12,16 | 12 | 10 | 11 | 25 | 11 | 11 | 15 | 9 | 21 |
| Ar-M-003 | 15 | 13 | 23 | 29 | 19.2 | 14 | 14,19 | 12 | 10 | 11 | 20 | 11 | 11 | 14 | 10 | 21 |
| Ar-M-004 | 16 | 12 | 21 | 28 | 16 | 14 | 14,16 | 14 | 10 | 12 | 22 | 11 | 12 | 16 | 10 | 21 |
| Ar-M-005 | 14 | 13 | 23 | 29 | 18.2 | 14 | 13,19 | 12 | 11 | 13 | 23 | 11 | 11 | 14 | 10 | 20 |
| Ar-M-006 | 15 | 13 | 23 | 30 | 17.2 | 14 | 13,19 | 12 | 10 | 11 | 21 | 11 | 11 | 14 | 10 | 20 |
| Ar-M-007 | 16 | 15 | 23 | 31 | 18 | 14 | 14,16 | 13 | 11 | 11 | 21 | 13 | 11 | 14 | 9 | 19 |
| Ar-M-008 | 17 | 13 | 23 | 31 | 15 | 16 | 11,14 | 14 | 10 | 10 | 24 | 11 | 12 | 14 | 11 | 20 |
| Ar-M-009 | 16 | 14 | 22 | 30 | 17 | 13 | 12,15 | 13 | 9 | 12 | 24 | 15 | 10 | 15 | 11 | 19 |
| Ar-M-010 | 14 | 12 | 24 | 27 | 18 | 15 | 16,18 | 12 | 11 | 12 | 21 | 11 | 11 | 14 | 9 | 19 |
| Ar-M-011 | 15 | 13 | 23 | 29 | 17.2 | 14 | 13,18 | 12 | 10 | 13 | 20 | 11 | 11 | 14 | 10 | 19 |
| Ar-M-012 | 15 | 14 | 23 | 30 | 17 | 15 | 16,20 | 12 | 11 | 12 | 20 | 11 | 13 | 14 | 10 | 20 |
| Ar-M-013 | 14 | 14 | 25 | 31 | 17 | 14 | 13,16 | 12 | 11 | 11 | 23 | 12 | 12 | 16 | 9 | 19 |
| Ar-M-014 | 13 | 13 | 23 | 29 | 15 | 14 | 15,16 | 14 | 11 | 11 | 20 | 12 | 11 | 15 | 10 | 20 |
| Ar-M-015 | 15 | 15 | 23 | 32 | 16 | 14 | 14,17 | 13 | 10 | 11 | 22 | 13 | 11 | 14 | 9 | 19 |
| Ar-M-016 | 14 | 13 | 23 | 29 | 18.2 | 14 | 13,19 | 12 | 11 | 11 | 21 | 11 | 11 | 14 | 10 | 19 |
| Ar-M-017 | 13 | 13 | 22 | 29 | 18.2 | 14 | 13,18 | 12 | 11 | 12 | 21 | 11 | 11 | 14 | 10 | 20 |
| Ar-M-018 | 13 | 13 | 23 | 29 | 15 | 14 | 15,16 | 14 | 11 | 11 | 20 | 12 | 11 | 15 | 10 | 20 |
| Ar-M-019 | 13 | 13 | 24 | 30 | 12 | 16 | 14,18 | 12 | 10 | 12 | 22 | 11 | 12 | 15 | 9 | 19 |
| Ar-M-020 | 16 | 14 | 22 | 30 | 17 | 13 | 12,15 | 13 | 9 | 12 | 24 | 15 | 10 | 15 | 11 | 19 |
| Ar-M-021 | 15 | 13 | 23 | 29 | 17.2 | 14 | 13,18 | 12 | 10 | 13 | 20 | 11 | 11 | 14 | 10 | 19 |
| Ar-M-022 | 16 | 15 | 23 | 31 | 18 | 14 | 15,16 | 13 | 11 | 11 | 21 | 13 | 11 | 14 | 9 | 19 |
| Ar-M-023 | 14 | 13 | 23 | 29 | 18.2 | 14 | 13,19 | 12 | 11 | 11 | 21 | 11 | 11 | 14 | 10 | 20 |
| Ar-M-024 | 15 | 12 | 22 | 29 | 17 | 16 | 12,14 | 14 | 10 | 11 | 22 | 11 | 11 | 16 | 10 | 21 |
| Ar-M-025 | 16 | 11 | 22 | 26 | 15 | 14 | 12,17 | 11 | 10 | 12 | 22 | 14 | 13 | 15 | 10 | 19 |
| Ar-M-026 | 15 | 14 | 23 | 30 | 17 | 14 | 14,16 | 13 | 10 | 11 | 21 | 13 | 11 | 14 | 9 | 19 |
| Ar-M-028 | 15 | 13 | 23 | 29 | 16 | 14 | 11,15 | 12 | 10 | 12 | 21 | 11 | 10 | 15 | 9 | 20 |
| Ar-M-029 | 17 | 13 | 26 | 30 | 15 | 16 | 11,14 | 13 | 11 | 11 | 23 | 11 | 13 | 14 | 11 | 20 |
| Ar-M-030 | 15 | 13 | 25 | 30 | 16 | 16 | 11,13 | 13 | 11 | 10 | 23 | 11 | 13 | 14 | 11 | 20 |
| Ar-M-031 | 14 | 14 | 23 | 30 | 18.2 | 14 | 13,19 | 12 | 11 | 11 | 22 | 11 | 11 | 14 | 10 | 20 |
| Ar-M-032 | 15 | 14 | 23 | 30 | 16 | 14 | 13,15 | 12 | 10 | 10 | 23 | 11 | 11 | 14 | 9 | 20 |
| Ar-M-033 | 14 | 13 | 23 | 32 | 17.2 | 14 | 13,19 | 12 | 10 | 11,12 | 21 | 11 | 11 | 14 | 10 | 20 |
| Ar-M-034 | 17 | 13 | 24 | 30 | 16 | 14 | 18,20 | 13 | 10 | 12 | 20 | 11 | 12 | 14 | 10 | 20 |
| Ar-M-035 | 13 | 13 | 23 | 30 | 19.2 | 14 | 13,19 | 12 | 12 | 11 | 21 | 11 | 11 | 14 | 10 | 20 |
| Ar-M-036 | 14 | 12 | 24 | 29 | 16 | 14 | 14,14 | 12 | 11 | 11 | 21 | 11 | 12 | 14 | 9 | 19 |
| Ar-M-037 | 15 | 13 | 23 | 30 | 18 | 14 | 14,16 | 13 | 10 | 11 | 21 | 13 | 11 | 14 | 9 | 19 |
| Ar-M-038 | 16 | 13 | 25 | 30 | 17 | 17 | 9,11 | 13 | 11 | 10 | 23 | 11 | 12 | 14 | 11 | 20 |
| Ar-M-039 | 15 | 13 | 23 | 29 | 14 | 15 | 13,17 | 12 | 10 | 12 | 21 | 11 | 10 | 14 | 9 | 21 |
| Ar-M-040 | 15 | 13 | 23 | 30 | 15 | 15 | 13,17 | 11 | 10 | 12 | 22 | 13 | 10 | 16 | 10 | 19 |
| Ar-M-041 | 14 | 13 | 23 | 30 | 19.2 | 14 | 13,19 | 12 | 11 | 11 | 21 | 11 | 11 | 14 | 10 | 20 |
| Ar-M-043 | 14 | 13 | 23 | 29 | 18.2 | 14 | 13,19 | 12 | 12 | 12 | 21 | 11 | 11 | 14 | 10 | 20 |
| Ar-M-044 | 14 | 13 | 23 | 29 | 18.2 | 14 | 13,19 | 12 | 11 | 11 | 22 | 11 | 11 | 14 | 10 | 20 |
| Ar-M-045 | 15 | 14 | 23 | 30 | 16 | 14 | 14,16 | 13 | 10 | 12 | 21 | 13 | 11 | 14 | 9 | 19 |
| Ar-M-046 | 16 | 13 | 23 | 29 | 15 | 15 | 13,16 | 12 | 9 | 12 | 22 | 11 | 12 | 14 | 9 | 21 |
| Ar-M-047 | 14 | 13 | 23 | 29 | 18.2 | 14 | 13,18 | 12 | 11 | 11 | 21 | 11 | 11 | 14 | 10 | 20 |
| Ar-M-048 | 15 | 13 | 25 | 30 | 16 | 15 | 11,14 | 13 | 10 | 10 | 23 | 11 | 13 | 14 | 11 | 20 |
| Ar-M-050 | 15 | 14 | 23 | 30 | 18.2 | 16 | 12,22 | 12 | 10 | 13 | 20 | 11 | 12 | 14 | 10 | 21 |
| Ar-M-051 | 15 | 14 | 24 | 31 | 17 | 16 | 11,14 | 13 | 10 | 10 | 24 | 11 | 11 | 14 | 11 | 20 |
| Ar-M-052 | 15 | 13 | 25 | 30 | 16 | 15 | 11,14 | 13 | 10 | 10 | 23 | 11 | 13 | 14 | 11 | 20 |
| Ar-M-053 | 14 | 13 | 23 | 30 | 18.2 | 14 | 13,18 | 12 | 11 | 11 | 22 | 11 | 11 | 14 | 10 | 20 |
| Ar-M-054 | 14 | 13 | 23 | 29 | 18.2 | 14 | 19,19 | 12 | 11 | 11 | 21 | 11 | 11 | 14 | 10 | 20 |
| Ar-M-055 | 15 | 14 | 23 | 30 | 20.2 | 14 | 13,20 | 12 | 10 | 11 | 23 | 11 | 11 | 14 | 10 | 21 |
| Ar-M-056 | 14 | 14 | 23 | 30 | 18.2 | 14 | 13,19 | 12 | 12 | 11 | 21 | 11 | 11 | 14 | 10 | 20 |
| Ar-M-057 | 16 | 13 | 24 | 31 | 16 | 16 | 11,14 | 13 | 11 | 10 | 23 | 11 | 12 | 14 | 11 | 20 |
| Ar-M-058 | 16 | 14 | 22 | 30 | 17 | 13 | 12,15 | 13 | 9 | 12 | 24 | 15 | 10 | 15 | 11 | 19 |
| Ar-M-059 | 16 | 13 | 24 | 28 | 18 | 14 | 12,14 | 12 | 12 | 12 | 23 | 14 | 13 | 15 | 13 | 19 |
| Ar-M-060 | 14 | 10,13 | 23 | 29 | 18.2 | 14 | 13,18 | 12 | 11 | 11 | 21 | 11 | 11 | 14 | 10 | 20 |
| Ar-M-061 | 15 | 14 | 23 | 31 | 20.2 | 14 | 13,20 | 12 | 10 | 11 | 23 | 11 | 11 | 14 | 10 | 21 |
| Ar-M-062 | 15 | 13 | 25 | 31 | 15 | 15 | 11,11 | 13 | 11 | 10 | 23 | 11 | 12 | 14 | 10 | 20 |
| Ar-M-063 | 16 | 14 | 23 | 31 | 17 | 13 | 15,16 | 13 | 10 | 12 | 21 | 13 | 11 | 14 | 9 | 19 |
| Ar-M-064 | 12 | 12 | 23 | 28 | 16 | 15 | 13,17 | 12 | 10 | 11 | 21 | 11 | 11 | 15 | 9 | 19 |
| Ar-M-065 | 15 | 12 | 24 | 29 | 18.2 | 14 | 11,16 | 12 | 10 | 11 | 20 | 11 | 11 | 14 | 10 | 20 |
| Ar-M-066 | 15 | 13 | 25 | 30 | 18 | 13 | 16,17 | 13 | 10 | 10 | 22 | 11 | 12 | 14 | 10 | 20 |
| Ar-M-067 | 16 | 13 | 23 | 29 | 15 | 14 | 12,20 | 12 | 10 | 11 | 21 | 11 | 13 | 15 | 9 | 19 |
| Ar-M-068 | 15 | 12 | 25 | 29 | 14 | 14 | 16,19 | 13 | 10 | 12 | 21 | 11 | 12 | 14 | 10 | 20 |
| Ar-M-069 | 15 | 13 | 24 | 29 | 15 | 16 | 13,17 | 13 | 11 | 11 | 21 | 13 | 11 | 14 | 9 | 20 |
| Ar-M-070 | 14 | 13 | 23 | 29 | 18.2 | 14 | 13,19 | 12 | 12 | 11 | 22 | 11 | 11 | 14 | 10 | 20 |
| Ar-M-071 | 14 | 13 | 23 | 29 | 18.2 | 14 | 13,19 | 12 | 11 | 12 | 22 | 11 | 11 | 14 | 10 | 20 |
| Ar-M-072 | 16 | 12 | 22 | 28 | 14 | 14 | 14,16 | 11 | 11 | 14 | 25 | 14 | 12 | 15 | 10 | 19 |
| Ar-M-073 | 14 | 14 | 23 | 30 | 18.2 | 14 | 13,19 | 12 | 12 | 11 | 21 | 11 | 11 | 14 | 10 | 20 |
| Ar-M-074 | 15 | 13 | 22 | 30 | 16 | 14 | 12,15 | 12 | 10 | 11 | 22 | 11 | 10 | 14 | 9 | 21 |
| Ar-M-075 | 15 | 13 | 23 | 30 | 18.2 | 14 | 13,19 | 12 | 11 | 11 | 21 | 11 | 11 | 14 | 10 | 20 |
| Ar-M-076 | 14 | 13 | 23 | 30 | 19.2 | 14 | 13,18 | 12 | 11 | 11 | 21 | 11 | 11 | 14 | 10 | 20 |
| Ar-M-077 | 16 | 13 | 26 | 30 | 16 | 16 | 11,14 | 13 | 11 | 10 | 23 | 11 | 13 | 15 | 11 | 20 |
| Ar-M-078 | 15 | 12 | 23 | 29 | 16 | 15 | 18,19 | 13 | 10 | 12 | 21 | 11 | 12 | 14 | 10 | 20 |
| Ar-M-079 | 16 | 13 | 24 | 30 | 17 | 14 | 12,15 | 12 | 10 | 11 | 21 | 11 | 11 | 14 | 9 | 20 |
| Ar-M-080 | 14 | 14 | 23 | 30 | 18.2 | 14 | 14,19 | 12 | 9 | 11 | 21 | 11 | 11 | 14 | 10 | 21 |
| Ar-M-082 | 13 | 13 | 22 | 29 | 21.2 | 15 | 13,18 | 12 | 11 | 13 | 22 | 11 | 11 | 14 | 10 | 20 |
| Ar-M-083 | 16 | 13 | 23 | 29 | 15 | 15 | 11,12 | 12 | 11 | 13 | 24 | 13 | 12 | 15 | 12 | 19 |
| Ar-M-084 | 15 | 13 | 25 | 30 | 15 | 15 | 11,14 | 13 | 10 | 10 | 23 | 11 | 13 | 14 | 11 | 20 |
| Ar-M-085 | 15 | 12 | 22 | 29 | 16 | 15 | 14,16 | 14 | 10 | 11 | 22 | 10 | 12 | 16 | 10 | 20 |
| Ar-M-086 | 15 | 14 | 24 | 31 | 18 | 13 | 16,17 | 13 | 10 | 13 | 22 | 11 | 10 | 14 | 10 | 20 |
| Ar-M-087 | 13 | 13 | 23 | 29 | 18.2 | 14 | 13,18 | 12 | 11 | 11 | 21 | 11 | 11 | 14 | 10 | 20 |
| Ar-M-088 | 14 | 13 | 23 | 29 | 19.2 | 14 | 13,18 | 12 | 11 | 11 | 21 | 11 | 11 | 14 | 10 | 20 |
| Ar-M-089 | 16 | 14 | 23 | 31 | 18 | 14 | 15,16 | 13 | 11 | 12 | 21 | 13 | 12 | 14 | 10 | 19 |
| Ar-M-090 | 15 | 13 | 25 | 30 | 16 | 15 | 11,14 | 13 | 10 | 11 | 23 | 11 | 13 | 14 | 11 | 20 |
| Ar-M-091 | 15 | 14 | 23 | 30 | 18.2 | 16 | 12,22 | 12 | 10 | 13 | 20 | 11 | 12 | 14 | 10 | 21 |
| Ar-M-092 | 16 | 11 | 22 | 26 | 15 | 14 | 12,17 | 11 | 10 | 12 | 22 | 14 | 13 | 15 | 10 | 19 |
| Ar-M-093 | 14 | 14 | 23 | 30 | 18.2 | 14 | 13,19 | 12 | 11 | 11 | 21 | 11 | 11 | 14 | 10 | 20 |
| Ar-M-095 | 15 | 12 | 22 | 30 | 17 | 13 | 16,17 | 13 | 10 | 12 | 21 | 11 | 9 | 14 | 10 | 20 |
| Ar-M-096 | 14 | 13 | 23 | 30 | 19.2 | 14 | 13,19 | 12 | 11 | 11 | 21 | 11 | 11 | 14 | 10 | 20 |
| Ar-M-097 | 19 | 13 | 24 | 30 | 15 | 13 | 18,18 | 13 | 11 | 11 | 22 | 11 | 12 | 14 | 10 | 20 |
| Ar-M-098 | 15 | 13 | 23 | 30 | 18.2 | 14 | 13,19 | 12 | 11 | 11 | 21 | 11 | 11 | 14 | 10 | 20 |
| Ar-M-099 | 14 | 13 | 23 | 29 | 18.2 | 14 | 13,18 | 12 | 11 | 11 | 21 | 11 | 11 | 14 | 10 | 20 |
| Ar-M-101 | 15 | 12 | 24 | 28 | 16 | 15 | 13,16 | 15 | 10 | 11 | 22 | 14 | 11 | 14 | 11 | 19 |
| Ar-M-102 | 15 | 13 | 24 | 29 | 17 | 14 | 11,14 | 13 | 10 | 12 | 24 | 13 | 12 | 15 | 13 | 19 |
| Ar-M-103 | 16 | 13 | 23 | 30 | 15 | 14 | 15,19 | 12 | 10 | 11 | 22 | 11 | 12 | 15 | 9 | 21 |
| Ar-M-104 | 15 | 13 | 25 | 30 | 16 | 15 | 11,14 | 13 | 10 | 10 | 23 | 11 | 13 | 14 | 11 | 20 |
| Ar-M-105 | 15 | 13 | 22 | 29 | 17 | 13 | 14,16 | 13 | 11 | 12 | 22 | 15 | 10 | 14 | 11 | 21 |
| Ar-M-106 | 15 | 13 | 23 | 27 | 15 | 14 | 13,15 | 12 | 10 | 10 | 24 | 11 | 10 | 15 | 9 | 20 |
| Ar-M-107 | 14 | 13 | 23 | 29 | 19.2 | 14 | 13,19 | 12 | 11 | 11 | 22 | 11 | 11 | 14 | 10 | 20 |
| Ar-M-108 | 13 | 13 | 22 | 29 | 19.2 | 14 | 12,17 | 12 | 11 | 12 | 21 | 11 | 11 | 14 | 10 | 20 |
| Kr-M-001 | 17 | 13 | 23 | 29 | 16 | 15 | 12,16 | 12 | 9 | 12 | 22 | 11 | 12 | 14 | 9 | 22 |
| Kr-M-003 | 16 | 13 | 23 | 29 | 16 | 16 | 13,16 | 12 | 9 | 12 | 21 | 11 | 11 | 14 | 9 | 21 |
| Kr-M-004 | 16 | 14 | 23 | 29 | 16 | 14 | 13,20 | 14 | 10 | 11 | 24 | 10 | 12 | 15 | 11 | 20 |
| Kr-M-005 | 15 | 14 | 22 | 30 | 16 | 15 | 13,19 | 12 | 9 | 10 | 23 | 11 | 11 | 16 | 9 | 20 |
| Kr-M-006 | 16 | 13 | 23 | 29 | 15 | 15 | 13,16 | 12 | 10 | 11 | 23 | 11 | 11 | 15 | 9 | 20 |
| Kr-M-007 | 15 | 13 | 23 | 30 | 17 | 15 | 19,19 | 14 | 10 | 12 | 25 | 11 | 13 | 14 | 10 | 20 |
| Kr-M-008 | 17 | 13 | 24 | 26 | 18 | 16 | 14,21 | 14 | 11 | 11 | 24 | 10 | 12 | 14 | 11 | 19 |
| Kr-M-009 | 14 | 13 | 22 | 29 | 19 | 13 | 15.2,17 | 13 | 10 | 12 | 22 | 15 | 10 | 14 | 11 | 19 |
| Kr-M-010 | 15 | 14 | 24 | 32 | 16 | 15 | 10,14 | 12 | 11 | 10 | 23 | 11 | 12 | 14 | 11 | 20 |
| Kr-M-011 | 15 | 13 | 24 | 31 | 21 | 14 | 16,17 | 13 | 10 | 11 | 21 | 11 | 11 | 14 | 10 | 20 |
| Kr-M-012 | 15 | 13 | 22 | 29 | 17 | 15 | 9,17 | 12 | 10 | 11 | 22 | 15 | 12 | 15 | 10 | 20 |
| Kr-M-013 | 15 | 14 | 22 | 29 | 17 | 14 | 13,22 | 14 | 10 | 12 | 26 | 10 | 11 | 14 | 11 | 19 |
| Kr-M-014 | 14 | 13 | 23 | 30 | 19.2 | 14 | 13,19 | 12 | 11 | 12 | 21 | 11 | 11 | 14 | 10 | 20 |
| Kr-M-015 | 15 | 13 | 23 | 32 | 16 | 15 | 11,16 | 13 | 10 | 10 | 23 | 11 | 13 | 14 | 11 | 20 |
| Kr-M-016 | 16 | 12 | 24 | 27 | 18 | 14 | 11,14 | 12 | 11 | 12 | 23 | 14 | 13 | 15 | 12 | 19 |
| Kr-M-018 | 16 | 13 | 24 | 28 | 18 | 14 | 11,14 | 12 | 10 | 12 | 23 | 14 | 13 | 15 | 12 | 19 |
| Kr-M-019 | 15 | 14 | 24 | 31 | 17 | 15 | 12,13 | 13 | 10 | 11 | 22 | 11 | 11 | 15 | 10 | 20 |
| Kr-M-020 | 16 | 14 | 24 | 31 | 17 | 15 | 11,15 | 13 | 11 | 10 | 23 | 11 | 12 | 14 | 11 | 20 |
| Kr-M-021 | 14 | 13 | 22 | 29 | 19 | 13 | 15.2,17 | 13 | 10 | 12 | 22 | 15 | 10 | 14 | 11 | 19 |
| Kr-M-022 | 15 | 13 | 24 | 30 | 17 | 14 | 14,18 | 12 | 10 | 11 | 21 | 11 | 12 | 14 | 9 | 20 |
| Kr-M-023 | 15 | 12 | 24 | 30 | 19.2 | 14 | 13,19 | 12 | 10 | 12 | 21 | 11 | 11 | 14 | 10 | 20 |
| Kr-M-024 | 16 | 12 | 23 | 30 | 20 | 15 | 13,14 | 13 | 10 | 13 | 21 | 12 | 11 | 16 | 10 | 20 |
| Kr-M-025 | 16 | 13 | 23 | 29 | 15 | 14 | 13,17 | 12 | 10 | 10 | 21 | 11 | 11 | 14 | 9 | 19 |
| Kr-M-026 | 15 | 13 | 22 | 29 | 16 | 14 | 13,16 | 12 | 11 | 12 | 23 | 11 | 12 | 15 | 9 | 20 |
| Kr-M-027 | 15 | 12 | 24 | 30 | 19.2 | 14 | 13,19 | 12 | 10 | 11 | 21 | 11 | 11 | 14 | 10 | 20 |
| Kr-M-028 | 15 | 12 | 25 | 29 | 18.2 | 13 | 11,19 | 12 | 10 | 12 | 21 | 11 | 10 | 14 | 10 | 20 |
| Kr-M-029 | 14 | 12 | 22 | 29 | 17 | 17 | 14,16 | 14 | 11 | 11 | 20 | 10 | 13 | 16 | 10 | 20 |
| Kr-M-030 | 16 | 12 | 23 | 30 | 20 | 15 | 13,15 | 13 | 10 | 13 | 22 | 12 | 11 | 16 | 10 | 20 |
| Kr-M-031 | 16 | 13 | 24 | 31 | 16 | 13 | 16,17 | 13 | 10 | 12 | 21 | 11 | 11 | 14 | 10 | 20 |
| Kr-M-032 | 15 | 12 | 24 | 29 | 15 | 17 | 11,14 | 13 | 10 | 10 | 23 | 11 | 12 | 14 | 11 | 19 |
| Kr-M-033 | 15 | 12 | 25 | 29 | 18.2 | 13 | 11,19 | 12 | 10 | 12 | 21 | 11 | 10 | 14 | 10 | 20 |
| Kr-M-034 | 15 | 12 | 24 | 30 | 19.2 | 14 | 13,19 | 12 | 10 | 12 | 21 | 11 | 11 | 14 | 10 | 20 |
| Kr-M-035 | 15 | 13 | 23 | 30 | 18.2 | 14 | 14,18 | 12 | 10 | 11 | 21 | 12 | 8 | 14 | 10 | 21 |
| Kr-M-036 | 15 | 12 | 22 | 27 | 16 | 16 | 12,12 | 15 | 10 | 11 | 21 | 11 | 11 | 16 | 10 | 20 |
| Kr-M-038 | 15 | 14 | 22 | 30 | 19 | 15 | 15,16 | 12 | 10 | 11 | 20 | 11 | 12 | 14 | 9 | 19 |
| Kr-M-039 | 15 | 12 | 25 | 29 | 18.2 | 13 | 11,19 | 12 | 10 | 12 | 21 | 11 | 10 | 14 | 10 | 20 |
| Kr-M-040 | 17 | 12 | 22 | 28 | 14 | 14 | 15,17 | 11 | 10 | 13 | 24 | 14 | 12 | 15 | 10 | 16.4 |
| Kr-M-041 | 15 | 13 | 22 | 29 | 17 | 15 | 12,12 | 14 | 10 | 14 | 21 | 11 | 11 | 16 | 11 | 20 |
| Kr-M-042 | 15 | 13 | 25 | 30 | 16 | 15 | 11,14 | 13 | 11 | 10 | 23 | 11 | 13 | 14 | 11 | 20 |
| Kr-M-043 | 15 | 12 | 23 | 28 | 16 | 14 | 11,14 | 12 | 11 | 13 | 25 | 13 | 12 | 14 | 12 | 19 |
| Kr-M-044 | 15 | 13 | 23 | 30 | 16 | 15 | 19,19 | 14 | 10 | 11 | 24 | 11 | 14 | 14 | 10 | 20 |
| Kr-M-045 | 15 | 13 | 22 | 28 | 15 | 15 | 11,18 | 12 | 10 | 11 | 24 | 11 | 11 | 15 | 9 | 20 |
| Kr-M-046 | 15 | 13 | 23 | 29 | 19.2 | 14 | 13,19 | 12 | 10 | 14 | 20 | 11 | 12 | 14 | 10 | 20 |
| Kr-M-047 | 16 | 12 | 24 | 31 | 15 | 13 | 15,18 | 13 | 10 | 12 | 22 | 11 | 11 | 14 | 10 | 20 |
| Kr-M-048 | 12 | 12 | 24 | 30 | 17 | 13 | 16,17 | 12 | 11 | 14 | 22 | 11 | 11 | 14 | 10 | 20 |
| Kr-M-049 | 14 | 13 | 23 | 31 | 17 | 13 | 18,19 | 13 | 10 | 13 | 23 | 11 | 11 | 14 | 10 | 19 |
| Kr-M-050 | 16 | 12 | 22 | 29 | 17 | 15 | 14,19 | 12 | 10 | 12 | 21 | 11 | 11 | 15 | 9 | 21 |
| Kr-M-051 | 14 | 14 | 23 | 30 | 16 | 14 | 13,16 | 12 | 10 | 11 | 24 | 11 | 12 | 15 | 9 | 21 |
| Kr-M-053 | 16 | 13 | 25 | 29 | 15 | 17 | 11,14 | 13 | 11 | 10 | 23 | 11 | 12 | 14 | 11 | 20 |
| Kr-M-054 | 14 | 13 | 25 | 29 | 15 | 15 | 14,18 | 12 | 10 | 13 | 21 | 11 | 11 | 15 | 9 | 18 |
| Kr-M-055 | 17 | 13 | 23 | 29 | 16 | 15 | 13,15 | 12 | 10 | 10 | 23 | 11 | 11 | 15 | 9 | 20 |
| Kr-M-056 | 15 | 14 | 24 | 31 | 15 | 14 | 16,17 | 13 | 9 | 12 | 20 | 11 | 12 | 14 | 10 | 20 |
| Kr-M-057 | 15 | 12 | 25 | 29 | 18.2 | 13 | 12,18 | 12 | 11 | 12 | 21 | 11 | 10 | 14 | 10 | 20 |
| Kr-M-058 | 15 | 12 | 24 | 29 | 15 | 17 | 11,14 | 13 | 10 | 10 | 23 | 11 | 12 | 14 | 11 | 19 |
| Kr-M-059 | 15 | 12 | 22 | 28 | 16 | 16 | 12,14 | 15 | 10 | 11 | 21 | 11 | 11 | 16 | 10 | 20 |
| Kr-M-061 | 15 | 13 | 23 | 30 | 18.2 | 15 | 13,18 | 12 | 10 | 10 | 20 | 11 | 11 | 14 | 10 | 23 |
| Kr-M-062 | 15 | 13 | 23 | 32 | 15 | 15 | 13,16 | 12 | 10 | 11 | 23 | 11 | 11 | 14 | 9 | 20 |
| Kr-M-063 | 17 | 13 | 23 | 29 | 16 | 15 | 13,15 | 12 | 10 | 10 | 23 | 11 | 11 | 15 | 9 | 20 |
| Kr-M-064 | 16 | 13 | 22 | 29 | 15 | 14 | 14,15 | 12 | 10 | 12 | 21 | 11 | 11 | 14 | 9 | 20 |
| Kr-M-065 | 15 | 13 | 24 | 29 | 16 | 14 | 14,16 | 12 | 10 | 11 | 21 | 11 | 11 | 15 | 9 | 20 |
| Kr-M-066 | 15 | 12 | 25 | 30 | 18.2 | 13 | 12,19 | 12 | 10 | 12 | 21 | 11 | 10 | 14 | 10 | 20 |
| Kr-M-067 | 15 | 14 | 23 | 32 | 15 | 15 | 13,16 | 12 | 10 | 11 | 23 | 11 | 11 | 14 | 9 | 20 |
| Kr-M-068 | 15 | 14 | 22 | 30 | 16 | 14 | 13,18 | 12 | 9 | 10 | 22 | 11 | 11 | 15 | 9 | 21 |
| Kr-M-069 | 15 | 12 | 23 | 30 | 17 | 13 | 16,17 | 14 | 9 | 12 | 20 | 11 | 12 | 14 | 10 | 20 |
| Kr-M-070 | 15 | 12 | 25 | 30 | 18.2 | 13 | 12,19 | 12 | 10 | 12 | 21 | 11 | 10 | 14 | 10 | 20 |
| Kr-M-071 | 15 | 13 | 24 | 31 | 14 | 13 | 15,15 | 14 | 11 | 11 | 21 | 11 | 12 | 14 | 10 | 20 |
| Kr-M-072 | 15 | 13 | 24 | 30 | 16 | 15 | 11,14 | 13 | 11 | 10 | 23 | 11 | 12 | 14 | 11 | 20 |
| Kr-M-073 | 15 | 14 | 22 | 30 | 16 | 15 | 13,17 | 12 | 9 | 10 | 23 | 11 | 11 | 15 | 9 | 22 |
| Kr-M-075 | 15 | 13 | 25 | 30 | 16 | 15 | 11,14 | 13 | 11 | 10 | 23 | 11 | 12 | 14 | 11 | 20 |
| Kr-M-076 | 15 | 14 | 24 | 33 | 16 | 13 | 16,16 | 13 | 10 | 13 | 22 | 11 | 11 | 14 | 10 | 20 |
| Kr-M-077 | 15 | 13 | 24 | 29 | 16 | 14 | 12,18 | 12 | 10 | 11 | 23 | 11 | 11 | 15 | 9 | 20 |
| Kr-M-078 | 16 | 12 | 22 | 28 | 16 | 14 | 13,16 | 11 | 10 | 12 | 24 | 14 | 12 | 15 | 10 | 20 |
| Kr-M-079 | 16 | 13 | 25 | 30 | 16 | 16 | 11,15 | 13 | 11 | 10 | 23 | 11 | 13 | 14 | 11 | 20 |
| Kr-M-080 | 16 | 14 | 23 | 30 | 17 | 14 | 13,20 | 14 | 10 | 11 | 24 | 10 | 12 | 15 | 11 | 20 |
| Kr-M-081 | 16 | 14 | 25 | 32 | 17 | 15 | 11,14 | 13 | 11 | 10 | 23 | 11 | 12 | 14 | 11 | 20 |
| Kr-M-082 | 15 | 13 | 24 | 30 | 18 | 15 | 11,15 | 13 | 10 | 11 | 25 | 13 | 12 | 15 | 12 | 19 |
| Kr-M-083 | 15 | 13 | 25 | 30 | 15 | 16 | 11,15 | 13 | 11 | 11 | 23 | 11 | 13 | 14 | 11 | 20 |
| Kr-M-084 | 14 | 13 | 23 | 31 | 16 | 13 | 18,20 | 13 | 10 | 13 | 22 | 11 | 11 | 14 | 10 | 19 |
| Kr-M-085 | 16 | 13 | 25 | 29 | 15 | 15 | 11,14 | 13 | 11 | 10 | 23 | 11 | 12 | 14 | 11 | 20 |
| Kr-M-086 | 15 | 12 | 24 | 29 | 15 | 17 | 11,15 | 13 | 10 | 10 | 23 | 11 | 12 | 14 | 11 | 19 |
| Kr-M-087 | 16 | 13 | 24 | 30 | 15 | 16 | 16,19 | 13 | 10 | 11 | 22 | 11 | 12 | 15 | 10 | 20 |
| Kr-M-088 | 15 | 13 | 23 | 30 | 16 | 15 | 18,20 | 14 | 10 | 12 | 25 | 11 | 13 | 14 | 10 | 20 |
| Kr-M-089 | 15 | 14 | 23 | 31 | 16 | 15 | 12,14 | 13 | 11 | 11 | 20 | 11 | 11 | 15 | 10 | 17 |
| Kr-M-090 | 15 | 14 | 23 | 31 | 16 | 15 | 12,14 | 13 | 11 | 11 | 20 | 11 | 11 | 15 | 10 | 17 |
| Kr-M-091 | 15 | 12 | 23 | 31 | 17 | 14 | 13,16 | 12 | 11 | 13 | 22 | 11 | 12 | 15 | 9 | 19 |
| Kr-M-092 | 15 | 12 | 25 | 29 | 18.2 | 13 | 11,19 | 12 | 10 | 12 | 21 | 11 | 10 | 14 | 10 | 20 |
| Kr-M-093 | 15 | 12 | 23 | 29 | 17 | 13 | 16,17 | 14 | 9 | 12 | 20 | 11 | 12 | 14 | 10 | 20 |
| Kr-M-094 | 15 | 13 | 25 | 30 | 19 | 15 | 13,16 | 12 | 10 | 13 | 21 | 11 | 11 | 15 | 9 | 18 |
| Kr-M-095 | 15 | 12 | 24 | 30 | 20.2 | 14 | 13,19 | 12 | 10 | 12 | 21 | 11 | 11 | 14 | 10 | 21 |
| Kr-M-096 | 15 | 13 | 23 | 30 | 16 | 15 | 19,19 | 14 | 9 | 13 | 25 | 11 | 14 | 14 | 10 | 20 |
| Kr-M-097 | 15 | 13 | 23 | 31 | 15 | 13 | 16,18 | 12 | 10 | 11 | 20 | 13 | 12 | 14 | 9 | 19 |
| Kr-M-098 | 15 | 13 | 23 | 29 | 17 | 14 | 14,16 | 12 | 10 | 11 | 23 | 11 | 11 | 15 | 9 | 20 |
| Kr-M-099 | 15 | 13 | 25 | 30 | 19 | 15 | 13,16 | 12 | 10 | 13 | 21 | 11 | 11 | 15 | 9 | 18 |
| Kr-M-100 | 17 | 14 | 24 | 31 | 16 | 17 | 9,11 | 13 | 10 | 10 | 23 | 11 | 12 | 14 | 11 | 20 |
| Kr-M-101 | 15 | 13 | 23 | 30 | 17.2 | 14 | 16,18 | 12 | 10 | 11 | 21 | 11 | 11 | 14 | 10 | 21 |
| Kr-M-102 | 17 | 13 | 24 | 30 | 15 | 16 | 16,20 | 13 | 10 | 11 | 22 | 11 | 12 | 15 | 10 | 20 |
| Kr-M-103 | 15 | 13 | 23 | 30 | 17.2 | 14 | 13,18 | 12 | 10 | 13 | 21 | 12 | 10 | 14 | 10 | 20 |
| Kr-M-104 | 15 | 12 | 23 | 28 | 18 | 15 | 14,14 | 13 | 10 | 11 | 21 | 11 | 11 | 15 | 10 | 20 |
| Kr-M-105 | 15 | 12 | 26 | 28 | 16 | 15 | 11,15 | 14 | 10 | 10 | 23 | 11 | 12 | 14 | 11 | 20 |
| Kr-M-106 | 15 | 13 | 23 | 29 | 15 | 15 | 13,17 | 12 | 10 | 11 | 21 | 11 | 11 | 15 | 9 | 20 |
| Kr-M-107 | 15 | 12 | 21 | 29 | 18 | 15 | 13,15 | 14 | 10 | 12 | 20 | 11 | 11 | 16 | 10 | 22 |
| Kr-M-108 | 15 | 12 | 23 | 30 | 17 | 14 | 14,16 | 14 | 10 | 11 | 22 | 11 | 12 | 15 | 10 | 21 |
| Kr-M-109 | 15 | 13 | 23 | 29 | 17 | 14 | 14,16 | 12 | 10 | 11 | 23 | 11 | 11 | 15 | 9 | 20 |
| Kr-M-110 | 15 | 14 | 24 | 30 | 16 | 15 | 11,14 | 13 | 11 | 10 | 23 | 11 | 12 | 14 | 11 | 20 |
| Sy-M-001 | 15 | 13 | 23 | 29 | 17 | 14 | 11,15 | 12 | 11 | 12 | 24 | 13 | 12 | 15 | 12 | 19 |
| Sy-M-002 | 15 | 15 | 23 | 31 | 16 | 14 | 14,16 | 13 | 10 | 11 | 21 | 13 | 11 | 14 | 9 | 19 |
| Sy-M-003 | 15 | 12 | 23 | 28 | 19.2 | 14 | 12,18 | 12 | 10 | 12 | 22 | 11 | 11 | 14 | 10 | 22 |
| Sy-M-004 | 16 | 12 | 22 | 28 | 15 | 17 | 14,15 | 13 | 11 | 11 | 21 | 11 | 11 | 16 | 10 | 20 |
| Sy-M-005 | 15 | 13 | 23 | 30 | 17 | 14 | 11,14 | 12 | 11 | 14 | 24 | 13 | 12 | 15 | 12 | 19 |
| Sy-M-006 | 16 | 13 | 24 | 29 | 15 | 14 | 11,15 | 12 | 11 | 12 | 23 | 13 | 13 | 15 | 12 | 19 |
| Sy-M-007 | 15 | 13 | 24 | 28 | 17 | 14 | 11,13 | 12 | 10 | 12 | 23 | 14 | 12 | 15 | 12 | 19 |
| Sy-M-008 | 15 | 13 | 24 | 28 | 17 | 14 | 11,13 | 12 | 10 | 12 | 23 | 14 | 12 | 15 | 12 | 19 |
| Sy-M-009 | 15 | 12 | 23 | 28 | 19.2 | 14 | 12,18 | 12 | 10 | 12 | 22 | 11 | 11 | 14 | 10 | 22 |
| Sy-M-010 | 15 | 14 | 23 | 30 | 17 | 14 | 11,15 | 12 | 11 | 13 | 24 | 13 | 12 | 15 | 12 | 19 |
| Sy-M-011 | 15 | 14 | 23 | 31 | 19 | 13 | 13,17 | 12 | 10 | 11 | 23 | 11 | 11 | 15 | 9 | 19 |
| Sy-M-012 | 13 | 13 | 24 | 30 | 12 | 16 | 14,18 | 12 | 10 | 13 | 22 | 11 | 12 | 15 | 9 | 19 |
| Sy-M-013 | 16 | 13 | 25 | 29 | 15 | 16 | 11,13 | 13 | 10 | 8,11 | 23 |  | 12 | 14 | 11 | 19 |
| Sy-M-014 | 15 | 12 | 23 | 28 | 17 | 14 | 12,18 | 12 | 11 | 11 | 23 | 11 | 11 | 14 | 9 | 20 |
| Sy-M-015 | 17 | 13 | 24 | 30 | 15 | 15 | 11,14 | 13 | 11 | 11 | 23 | 11 | 12 | 14 | 11 | 19 |
| Sy-M-016 | 15 | 13 | 23 | 30 | 17.2 | 14 | 13,19 | 12 | 10 | 11 | 20 | 11 | 10 | 14 | 10 | 19 |
| Sy-M-017 | 16 | 13 | 24 | 29 | 15 | 14 | 11,15 | 12 | 11 | 12 | 23 | 13 | 13 | 15 | 12 | 19 |
| Sy-M-018 | 15 | 13 | 23 | 30 | 18.2 | 14 | 12,17 | 12 | 10 | 11 | 20 | 11 | 11 | 14 | 10 | 20 |
| Sy-M-019 | 16 | 14 | 23 | 30 | 17 | 14 | 15,16 | 12 | 10 | 11 | 21 | 13 | 11 | 14 | 9 | 19 |
| Sy-M-020 | 15 | 14 | 23 | 31 | 19 | 13 | 13,17 | 12 | 10 | 11 | 23 | 11 | 11 | 15 | 9 | 19 |
| Sy-M-021 | 16 | 13 | 22 | 30 | 17 | 14 | 12,15 | 12 | 10 | 11 | 22 | 11 | 11 | 14 | 9 | 22 |
| Sy-M-023 | 15 | 14 | 23 | 30 | 17 | 14 | 11,15 | 12 | 11 | 13 | 24 | 13 | 11 | 15 | 12 | 19 |
| Sy-M-024 | 16 | 13 | 24 | 29 | 15 | 16 | 11,13 | 13 | 10 | 11 | 23 | 12 | 12 | 14 | 11 | 19 |
| Sy-M-025 | 16 | 13 | 22 | 29 | 15 | 14 | 13,16 | 12 | 10 | 12 | 21 | 11 | 11 | 15 | 9 | 21 |
| Sy-M-026 | 15 | 14 | 23 | 31 | 19 | 14 | 13,17 | 12 | 10 | 11 | 23 | 11 | 11 | 15 | 9 | 20 |
| Sy-M-028 | 16 | 14 | 23 | 30 | 17 | 14 | 14,16 | 12 | 10 | 12 | 21 | 13 | 11 | 14 | 9 | 19 |
| Sy-M-029 | 16 | 14 | 23 | 30 | 17 | 14 | 14,16 | 12 | 10 | 11 | 21 | 13 | 11 | 14 | 9 | 19 |
| Sy-M-030 | 15 | 13 | 24 | 28 | 17 | 14 | 11,13 | 12 | 10 | 12 | 23 | 14 | 12 | 15 | 12 | 19 |
| Sy-M-031 | 16 | 13 | 22 | 29 | 15 | 14 | 13,16 | 12 | 10 | 12 | 21 | 11 | 11 | 15 | 9 | 21 |
| Sy-M-032 | 16 | 13 | 22 | 29 | 15 | 14 | 13,16 | 12 | 10 | 12 | 21 | 11 | 11 | 15 | 9 | 21 |
| Sy-M-033 | 17 | 14 | 22 | 32 | 15 | 14 | 14,19 | 12 | 10 | 12 | 22 | 11 | 11 | 14 | 9 | 21 |
| Sy-M-034 | 16 | 13 | 22 | 29 | 15 | 14 | 13,16 | 12 | 10 | 12 | 21 | 11 | 11 | 15 | 9 | 21 |
| Sy-M-035 | 12 | 13 | 24 | 30 | 16 | 14 | 11,14 | 12 | 10 | 12 | 23 | 13 | 13 | 15 | 12 | 19 |
| Sy-M-037 | 15 | 13 | 23 | 30 | 18.2 | 14 | 12,18 | 12 | 10 | 11 | 20 | 11 | 11 | 14 | 10 | 20 |
| Sy-M-038 | 15 | 13 | 24 | 28 | 17 | 14 | 11,13 | 12 | 10 | 12 | 23 | 14 | 12 | 15 | 12 | 19 |
| Sy-M-039 | 16 | 13 | 22 | 29 | 15 | 14 | 13,16 | 12 | 10 | 12 | 21 | 11 | 11 | 15 | 9 | 21 |
| Sy-M-040 | 15 | 11,13 | 23 | 25,29 | 17.2 | 14 | 13,18 | 12 | 10 | 11 | 20 | 11 | 11 | 14 | 10 |  |
| Sy-M-041 | 16 | 13 | 24 | 29 | 15 | 16 | 11,13 | 13 | 10 | 11 | 23 | 12 | 12 | 14 | 11 | 19 |
| Sy-M-042 | 16 | 13 | 25 | 29 | 15 | 16 | 11,13 | 13 | 10 | 11 | 23 | 12 | 12 | 14 | 11 | 19 |
| Sy-M-043 | 15 | 14 | 23 | 31 | 19 | 14 | 13,17 | 12 | 10 | 11 | 23 | 11 | 11 | 15 | 9 | 20 |
| Sy-M-045 | 15 | 13 | 23 | 29 | 17 | 14 | 11,15 | 12 | 10 | 12 | 24 | 13 | 12 | 15 | 12 | 19 |
| Sy-M-046 | 15 | 14 | 23 | 31 | 19 | 13 | 13,17 | 12 | 10 | 11 | 22 | 11 | 11 | 15 | 9 | 20 |
| Sy-M-047 | 16 | 14 | 23 | 30 | 17 | 14 | 14,16 | 12 | 10 | 11 | 21 | 13 | 11 | 14 | 9 | 19 |
| Sy-M-048 | 16 | 13 | 24 | 29 | 15 | 14 | 11,15 | 12 | 11 | 12 | 23 | 13 | 13 | 15 | 12 | 19 |
| Sy-M-049 | 15 | 14 | 23 | 30 | 12 | 14 | 15,17 | 12 | 10 | 12 | 21 | 11 | 11 | 15 | 9 | 20 |
| Sy-M-050 | 15 | 13 | 23 | 29 | 17.2 | 14 | 13,18 | 12 | 10 | 12 | 21 | 11 | 11 | 14 | 10 | 20 |
| Sy-M-051 | 15 | 14 | 24 | 30 | 16 | 14 | 11,14 | 13 | 10 | 12 | 23 | 13 | 13 | 15 | 12 | 19 |
| Sy-M-052 | 16 | 14 | 23 | 30 | 17 | 14 | 14,16 | 12 | 10 | 12 | 21 | 13 | 11 | 14 | 9 | 19 |
| Sy-M-053 | 15 | 13 | 24 | 28 | 17 | 14 | 11,13 | 12 | 10 | 12 | 23 | 14 | 12 | 15 | 12 | 19 |
| Sy-M-054 | 14 | 13 | 24 | 29 | 17 | 15 | 14,17 | 13 | 10 | 12 | 21 | 13 | 10 | 14 | 9 | 19 |
| Sy-M-055 | 16 | 13 | 22 | 29 | 15 | 14 | 13,16 | 12 | 10 | 12 | 21 | 11 | 11 | 15 | 9 | 21 |
| Sy-M-056 | 16 | 12 | 21 | 28 | 17 | 14 | 12,12 | 11 | 10 | 11 | 22 | 14 | 11 | 15 | 10 | 19 |
| Sy-M-057 | 17 | 14 | 22 | 32 | 15 | 14 | 14,19 | 12 | 10 | 12 | 22 | 11 | 11 | 14 | 9 | 21 |
| Sy-M-058 | 16 | 13 | 22 | 29 | 15 | 14 | 13,16 | 12 | 10 | 12 | 21 | 11 | 11 | 15 | 9 | 21 |
| Sy-M-059 | 16 | 14 | 24 | 30 | 18 | 14 | 15,17 | 12 | 10 | 12 | 21 | 13 | 11 | 14 | 9 | 19 |
| Sy-M-060 | 15 | 13 | 25 | 29 | 15 | 15 | 11,13 | 13 | 10 | 11 | 23 | 12 | 12 | 14 | 11 | 19 |
| Sy-M-061 | 15 | 12 | 23 | 28 | 19.2 | 14 | 12,18 | 12 | 10 | 12 | 22 | 11 | 11 | 14 | 10 | 22 |
| Sy-M-062 | 17 | 14 | 22 | 32 | 15 | 14 | 14,19 | 12 | 10 | 12 | 22 | 11 | 11 | 14 | 9 | 21 |
| Sy-M-063 | 15 | 12 | 24 | 30 | 17 | 13 | 15,17 | 12 | 10 | 12 | 22 | 11 | 11 | 14 | 10 | 20 |
| Sy-M-064 | 15 | 13 | 23 | 29 | 17 | 14 | 11,15 | 12 | 11 | 12 | 24 | 13 | 12 | 15 | 12 | 19 |
| Sy-M-065 | 16 | 13 | 23 | 29 | 17 | 14 | 11,15 | 12 | 11 | 12 | 24 | 13 | 12 | 15 | 12 | 19 |
| Sy-M-066 | 15 | 13 | 24 | 28 | 17 | 14 | 11,13 | 12 | 10 | 12 | 23 | 14 | 12 | 15 | 12 | 19 |
| Sy-M-067 | 15 | 13 | 23 | 30 | 18.2 | 14 | 12,18 | 12 | 10 | 11 | 20 | 11 | 11 | 14 | 10 | 20 |
| Sy-M-068 | 15 | 13 | 24 | 28 | 17 | 14 | 11,13 | 12 | 10 | 12 | 23 | 14 | 12 | 15 | 12 | 19 |
| Sy-M-069 | 15 | 13 | 25 | 29 | 15 | 14 | 11,13 | 12 | 10 | 12 | 24 | 13 | 13 | 15 | 12 | 19 |
| Sy-M-070 | 15 | 14 | 23 | 30 | 16 | 14 | 14,16 | 13 | 10 | 11 | 21 | 13 | 11 | 14 | 9 | 19 |
| Sy-M-071 | 16 | 13 | 25 | 29 | 15 | 15 | 11,13 | 13 | 10 | 11 | 23 | 12 | 12 | 14 | 11 | 19 |
| Sy-M-072 | 15 | 13 | 24 | 28 | 17 | 14 | 11,13 | 12 | 10 | 12 | 23 | 14 | 12 | 15 | 12 | 19 |
| Sy-M-073 | 16 | 13 | 24 | 30 | 15 | 13 | 16,17 | 13 | 11 | 12 | 21 | 11 | 11 | 14 | 10 | 20 |
| Sy-M-074 | 14 | 13 | 24 | 29 | 17 | 15 | 14,17 | 13 | 10 | 12 | 21 | 13 | 10 | 14 | 9 | 19 |
| Sy-M-075 | 15 | 13 | 23 | 29 | 17 | 14 | 11,15 | 12 | 11 | 12 | 24 | 13 | 12 | 15 | 12 | 19 |
| Sy-M-076 | 15 | 13 | 25 | 29 | 15 | 15 | 11,13 | 13 | 10 | 11 | 23 | 12 | 12 | 14 | 11 | 19 |
| Sy-M-077 | 15 | 13 | 23 | 29 | 17 | 14 | 11,15 | 12 | 11 | 12 | 24 | 13 | 12 | 15 | 12 | 19 |
| Sy-M-078 | 15 | 15 | 23 | 31 | 16 | 14 | 14,16 | 13 | 10 | 11 | 21 | 13 | 11 | 14 | 9 | 19 |
| Sy-M-079 | 15 | 14 | 23 | 30 | 16 | 14 | 14,16 | 13 | 10 | 11 | 21 | 13 | 11 | 14 | 9 | 19 |
| Sy-M-080 | 16 | 12 | 22 | 28 | 15 | 17 | 14,15 | 13 | 11 | 11 | 21 | 11 | 11 | 16 | 10 | 20 |
| Sy-M-081 | 15 | 14 | 23 | 30 | 16 | 14 | 14,16 | 13 | 10 | 11 | 21 | 13 | 11 | 14 | 9 | 19 |
| Sy-M-082 | 15 | 13 | 23 | 29 | 17 | 14 | 11,14 | 12 | 11 | 12 | 24 | 13 | 12 | 15 | 12 | 19 |
| Sy-M-084 | 15 | 14 | 23 | 30 | 18 | 14 | 13,17 | 12 | 10 | 11 | 23 | 11 | 11 | 15 | 9 | 20 |
| Sy-M-085 | 15 | 12 | 23 | 28 | 19.2 | 14 | 12,18 | 12 | 10 | 12 | 22 | 11 | 11 | 14 | 10 | 22 |
| Sy-M-086 | 15 | 14 | 23 | 30 | 16 | 14 | 14,16 | 13 | 10 | 11 | 21 | 13 | 11 | 14 | 9 | 19 |
| Sy-M-087 | 15 | 13 | 22 | 31 | 15 | 14 | 13,16 | 12 | 10 | 11 | 21 | 11 | 11 | 15 | 9 | 21 |
| Sy-M-088 | 15 | 13 | 24 | 28 | 17 | 14 | 11,13 | 12 | 10 | 12 | 23 | 14 | 12 | 15 | 12 | 19 |
| Sy-M-089 | 16 | 13 | 25 | 29 | 15 | 16 | 11,13 | 13 | 10 | 11 | 23 | 12 | 12 | 14 | 11 | 18 |
| Sy-M-091 | 16 | 14 | 23 | 30 | 17 | 14 | 14,16 | 12 | 10 | 12 | 21 | 13 | 11 | 14 | 9 | 19 |
| Sy-M-092 | 15 | 13 | 23 | 30 | 17 | 14 | 11,15 | 12 | 11 | 13 | 24 | 13 | 12 | 15 | 12 | 19 |
| Tm-M-001 | 15 | 12 | 22 | 29 | 17 | 15 | 13,14 | 13 | 10 | 11 | 21 | 11 | 13 | 16 | 10 | 21 |
| Tm-M-002 | 16 | 14 | 23 | 30 | 15 | 13 | 12,16 | 12 | 10 | 12 | 21 | 11 | 13 | 15 | 9 | 21 |
| Tm-M-003 | 15 | 12 | 23 | 30 | 17 | 14 | 14,15 | 14 | 10 | 11 | 22 | 11 | 12 | 15 | 10 | 21 |
| Tm-M-004 | 16 | 13 | 25 | 30 | 16 | 15 | 11,14 | 12 | 13 | 10 | 23 | 11 | 12 | 14 | 11 | 20 |
| Tm-M-005 | 15 | 13 | 25 | 30 | 16 | 16 | 11,13 | 13 | 11 | 10 | 23 | 11 | 13 | 14 | 11 | 20 |
| Tm-M-006 | 17 | 13 | 24 | 29 | 15 | 15 | 11,14 | 12 | 11 | 12 | 23 | 13 | 13 | 15 | 12 | 19 |
| Tm-M-007 | 15 | 13 | 25 | 31 | 16 | 15 | 11,16 | 13 | 10 | 10 | 23 | 11 | 12 | 14 | 11 | 19 |
| Tm-M-009 | 16 | 13 | 23 | 30 | 16 | 13 | 16,18 | 13 | 10 | 12 | 21 | 11 | 11 | 14 | 10 | 20 |
| Tm-M-010 | 17 | 13 | 24 | 30 | 16 | 13 | 16,19 | 13 | 10 | 11 | 22 | 11 | 12 | 14 | 10 | 20 |
| Tm-M-011 | 14 | 13 | 23 | 30 | 19.2 | 14 | 13,19 | 12 | 10 | 11 | 21 | 11 | 11 | 14 | 10 | 20 |
| Tm-M-012 | 15 | 13 | 24 | 30 | 16 | 16 | 11,14 | 13 | 11 | 10 | 23 | 11 | 12 | 14 | 11 | 20 |
| Tm-M-013 | 14 | 13 | 24 | 29 | 16 | 14 | 14,18 | 12 | 10 | 12 | 21 | 11 | 11 | 15 | 9 | 18 |
| Tm-M-014 | 15 | 14 | 25 | 32 | 17 | 13 | 16,18 | 13 | 10 | 12 | 22 | 11 | 10 | 14 | 10 | 19 |
| Tm-M-015 | 15 | 13 | 23 | 30 | 16 | 15 | 14,18 | 12 | 10 | 11 | 22 | 11 | 11 | 14 | 9 | 19 |
| Tm-M-017 | 15 | 14 | 23 | 30 | 15 | 15 | 13,15 | 12 | 11 | 12 | 25 | 11 | 12 | 16 | 10 | 21 |
| Tm-M-018 | 14 | 13 | 24 | 28 | 16 | 15 | 12,12 | 13 | 10 | 12 | 21 | 11 | 12 | 14 | 10 | 21 |
| Tm-M-019 | 16 | 12 | 22 | 28 | 18 | 14 | 13,18 | 12 | 10 | 11 | 23 | 11 | 11 | 14 | 9 | 21 |
| Tm-M-020 | 15 | 12 | 25 | 29 | 18.2 | 14 | 12,18 | 12 | 11 | 11 | 21 | 11 | 10 | 14 | 10 | 20 |
| Tm-M-021 | 15 | 13 | 23 | 29 | 16 | 14 | 13,17 | 12 | 10 | 12 | 20 | 11 | 12 | 14 | 9 | 21 |
| Tm-M-023 | 15 | 12 | 25 | 29 | 18.2 | 13 | 12,18 | 12 | 12 | 12 | 21 | 11 | 10 | 14 | 10 | 20 |
| Tm-M-024 | 16 | 14 | 23 | 30 | 17 | 14 | 13,17 | 12 | 10 | 11 | 22 | 11 | 13 | 15 | 9 | 20 |
| Tm-M-025 | 16 | 14 | 24 | 32 | 17 | 14 | 11,14 | 12 | 11 | 12 | 23 | 13 | 13 | 15 | 12 | 19 |
| Tm-M-026 | 17 | 12 | 22 | 28 | 14 | 14 | 15,17 | 11 | 10 | 13 | 24 | 14 | 12 | 15 | 10 | 16.4 |
| Tm-M-027 | 16 | 14 | 24 | 31 | 18 | 14 | 11,14 | 12 | 11 | 12 | 23 | 13 | 13 | 15 | 12 | 19 |
| Tm-M-028 | 15 | 14 | 25 | 32 | 17 | 13 | 16,17 | 13 | 10 | 12 | 22 | 11 | 10 | 14 | 10 | 19 |
| Tm-M-029 | 15 | 13 | 22 | 32 | 20.2 | 14 | 12,20 | 12 | 10 | 12 | 20 | 11 | 12 | 14 | 10 | 20 |
| Tm-M-030 | 15 | 13 | 23 | 30 | 17.2 | 14 | 12,15 | 12 | 10 | 12 | 21 | 11 | 11 | 14 | 10 | 21 |
| Tm-M-031 | 15 | 13 | 25 | 30 | 20.2 | 14 | 13,13 | 12 | 9 | 11 | 22 | 11 | 10 | 14 | 10 | 20 |
| Tm-M-033 | 16 | 14 | 24 | 32 | 17 | 14 | 11,14 | 12 | 11 | 12 | 23 | 13 | 13 | 15 | 12 | 19 |
| Tm-M-034 | 15 | 12 | 23 | 29 | 14 | 13 | 17,18 | 13 | 10 | 12 | 21 | 11 | 12 | 14 | 10 | 20 |
| Tm-M-035 | 15 | 13 | 24 | 31 | 21 | 14 | 16,17 | 13 | 10 | 12 | 21 | 11 | 11 | 14 | 10 | 20 |
| Tm-M-036 | 15 | 14 | 23 | 32 | 15 | 15 | 13,16 | 12 | 10 | 11 | 23 | 11 | 11 | 14 | 9 | 20 |
| Tm-M-037 | 15 | 13 | 23 | 30 | 16 | 15 | 19,19 | 14 | 10 | 12 | 25 | 11 | 14 | 14 | 10 | 20 |
| Tm-M-038 | 15 | 14 | 25 | 32 | 17 | 13 | 16,17 | 13 | 10 | 12 | 21 | 11 | 10 | 14 | 10 | 19 |
| Tm-M-039 | 17 | 12 | 21 | 29 | 16 | 15 | 14,15 | 14 | 10 | 12 | 23 | 11 | 11 | 15 | 11 | 24 |
| Tm-M-040 | 14 | 14 | 23 | 30 | 17 | 14 | 13,15 | 12 | 10 | 11 | 20 | 11 | 11 | 15 | 9 | 21 |
| Tm-M-041 | 15 | 14 | 23 | 32 | 15 | 15 | 13,16 | 12 | 10 | 11 | 23 | 11 | 11 | 14 | 9 | 20 |
| Tm-M-042 | 16 | 14 | 23 | 32 | 16 | 14 | 14,17 | 13 | 10 | 11 | 21 | 13 | 11 | 14 | 9 | 19 |
| Tm-M-043 | 16 | 14 | 23 | 31 | 16 | 15 | 11,14 | 13 | 11 | 10 | 24 | 11 | 12 | 14 | 11 | 20 |
| Tm-M-044 | 16 | 13 | 23 | 30 | 16 | 15 | 12,13 | 14 | 11 | 12 | 23 | 13 | 12 | 15 | 11 | 19 |
| Tm-M-045 | 15 | 12 | 25 | 29 | 18.2 | 13 | 12,18 | 12 | 11 | 12 | 21 | 11 | 10 | 14 | 10 | 20 |
| Tm-M-046 | 15 | 14 | 25 | 32 | 17 | 13 | 16,17 | 13 | 10 | 12 | 21 | 11 | 10 | 14 | 10 | 19 |
| Tm-M-047 | 16 | 13 | 25 | 31 | 16 | 15 | 11,15 | 14 | 10 | 10 | 23 | 11 | 13 | 14 | 11 | 20 |
| Tm-M-048 |  | 12 | 23 | 28 | 17 | 14 | 14,18 | 13 | 9 | 13 | 23 | 13 | 11 | 14 | 9 | 17 |
| Tm-M-049 | 16 | 12 | 24 | 31 | 15 | 13 | 17,17 | 13 | 10 | 13 | 23 | 11 | 11 | 14 | 10 | 20 |
| Tm-M-050 | 15 | 12 | 22 | 28 | 16 | 16 | 12,14 | 15 | 10 | 11 | 21 | 11 | 11 | 16 | 10 | 20 |
| Tm-M-051 | 15 | 13 | 23 | 29 | 16 | 15 | 16,16 | 12 | 10 | 12 | 21 | 11 | 12 | 16 | 9 | 21 |
| Tm-M-052 | 16 | 12 | 22 | 28 | 18 | 14 | 13,18 | 12 | 10 | 11 | 23 | 11 | 11 | 14 | 9 | 21 |
| Tm-M-053 | 15 | 12 | 23 | 28 | 17 | 14 | 12,18 | 13 | 10 | 10 | 25 | 10 | 12 | 16 | 11 | 19 |
| Tm-M-054 | 15 | 13 | 23 | 32 | 18.2 | 14 | 13,18 | 12 | 10 | 11 | 21 | 11 | 11 | 14 | 10 | 23 |
| Tm-M-055 | 16 | 13 | 25 | 31 | 15 | 16 | 12,15 | 13 | 10 | 10 | 23 | 11 | 13 | 15 | 11 | 21 |
| Tm-M-056 | 16 | 13 | 23 | 29 | 15 | 15 | 13,16 | 12 | 9 | 12 | 22 | 11 | 12 | 14 | 9 | 21 |
| Tm-M-057 | 15 | 13 | 23 | 31 | 15 | 13 | 16,17 | 12 | 10 | 11 | 20 | 13 | 11 | 14 | 9 | 19 |
| Tm-M-058 | 16 | 12 | 21 | 30 | 16 | 15 | 14,15 | 13 | 10 | 11 | 21 | 11 | 11 | 16 | 11 | 24 |
| Tm-M-059 | 16 | 12 | 21 | 30 | 16 | 15 | 14,15 | 13 | 10 | 11 | 21 | 11 | 11 | 16 | 11 | 24 |
| Tm-M-060 | 15 | 13 | 24 | 30 | 17 | 14 | 11,14 | 12 | 11 | 11 | 23 | 13 | 12 | 14 | 12 | 20 |
| Tm-M-061 | 15 | 12 | 23 | 28 | 17 | 14 | 12,18 | 13 | 10 | 10 | 25 | 10 | 12 | 16 | 11 | 19 |
| Tm-M-062 | 16 | 14 | 22 | 29 | 17 | 13 | 14,16 | 13 | 10 | 12 | 22 | 15 | 10 | 14 | 12 | 20 |
| Tm-M-063 | 15 | 14 | 24 | 32 | 14 | 14 | 16,16 | 14 | 9 | 11 | 22 | 11 | 12 | 14 | 10 | 20 |
| Tm-M-064 | 16 | 14 | 24 | 32 | 17 | 14 | 11,14 | 12 | 11 | 12 | 23 | 13 | 13 | 15 | 12 | 19 |
| Tm-M-065 | 15 | 13 | 23 | 31 | 15 | 15 | 13,16 | 12 | 10 | 11 | 23 | 11 | 11 | 14 | 9 | 20 |
| Tm-M-066 | 16 | 13 | 22 | 29 | 17 | 13 | 14,16 | 13 | 9 | 11 | 22 | 15 | 10 | 14 | 11 | 19 |
| Tm-M-067 | 15 | 12 | 25 | 29 | 18.2 | 13 | 12,19 | 12 | 11 | 13 | 22 | 11 | 10 | 14 | 10 | 20 |
| Tm-M-068 | 16 | 12 | 23 | 28 | 16 | 15 | 16,16 | 13 | 9 | 12 | 22 | 10 | 10 | 16 | 10 | 20 |
| Tm-M-069 | 17 | 13 | 23 | 30 | 16 | 13 | 16,19 | 13 | 10 | 12 | 22 | 11 | 12 | 14 | 10 | 20 |
| Tm-M-070 | 15 | 14 | 24 | 32 | 14 | 14 | 16,17 | 14 | 9 | 11 | 22 | 11 | 12 | 14 | 10 | 20 |
| Tm-M-071 | 17 | 14 | 23 | 31 | 17 | 14 | 14,16 | 13 | 10 | 11 | 21 | 13 | 11 | 14 | 9 | 19 |
| Tm-M-072 | 16 | 13 | 24 | 30 | 16 | 17 | 9,11 | 13 | 11 | 10 | 23 | 11 | 12 | 14 | 11 | 20 |
| Tm-M-073 | 16 | 14 | 23 | 32 | 17 | 15 | 15,16 | 13 | 10 | 11 | 21 | 14 | 11 | 14 | 9 | 18 |
| Tm-M-074 | 15 | 13 | 23 | 29 | 16 | 15 | 16,16 | 12 | 10 | 12 | 21 | 11 | 12 | 16 | 9 | 21 |
| Tm-M-075 | 16 | 12 | 22 | 29 | 16 | 15 | 14,14 | 13 | 11 | 11 | 21 | 11 | 11 | 16 | 10 | 21 |
| Tm-M-076 | 14 | 13 | 23 | 30 | 19.2 | 14 | 13,19 | 12 | 11 | 12 | 21 | 11 | 11 | 14 | 10 | 20 |
| Tm-M-077 | 15 | 14 | 23 | 32 | 15 | 15 | 13,16 | 12 | 10 | 11 | 23 | 11 | 11 | 14 | 9 | 20 |
| Tm-M-078 | 15 | 14 | 25 | 32 | 17 | 13 | 17,17 | 13 | 10 | 12 | 22 | 11 | 10 | 14 | 10 | 19 |
| Tm-M-079 | 16 | 14 | 26 | 30 | 16 | 16 | 11,14 | 13 | 11 | 10 | 23 | 11 | 12 | 14 | 11 | 20 |
| Tm-M-080 | 15 | 12 | 25 | 29 | 18.2 | 13 | 12,18 | 12 | 10 | 12 | 21 | 11 | 10 | 14 | 10 | 20 |
| Tm-M-081 | 15 | 13 | 25 | 30 | 16 | 14 | 11,14 | 12 | 11 | 11 | 23 | 13 | 12 | 15 | 12 | 19 |
| Tm-M-082 | 15 | 14 | 23 | 30 | 15 | 14 | 14,18 | 12 | 10 | 12 | 23 | 11 | 11 | 14 | 9 | 20 |
| Tm-M-084 | 15 | 13 | 23 | 30 | 17 | 15 | 14,15 | 13 | 11 | 12 | 23 | 11 | 11 | 15 | 10 | 20 |
| Tm-M-085 | 14 | 13 | 23 | 30 | 20.2 | 14 | 14,19 | 13 | 11 | 12 | 22 | 11 | 11 | 14 | 10 | 20 |
| Tm-M-086 | 15 | 14 | 22 | 29 | 15 | 14 | 11,12 | 13 | 10 | 12 | 20 | 12 | 12 | 15 | 11 | 19 |
| Tm-M-087 | 15 | 13 | 21 | 31 | 17 | 15 | 16,17 | 13 | 11 | 11 | 21 | 11 | 11 | 14 | 11 | 21 |
| Tm-M-088 | 15 | 12 | 24 | 30 | 17 | 13 | 15,15 | 12 | 10 | 12 | 22 | 11 | 11 | 14 | 10 | 21 |
| Tm-M-089 | 17 | 13 | 23 | 29 | 16 | 14 | 12,16 | 12 | 9 | 12 | 22 | 11 | 12 | 14 | 9 | 21 |
| Tm-M-090 | 15 | 13 | 23 | 29 | 17 | 14 | 13,15 | 12 | 10 | 11 | 21 | 11 | 10 | 15 | 9 | 20 |
| Tm-M-091 | 15 | 14 | 23 | 30 | 15 | 15 | 13,17 | 11 | 10 | 12 | 22 | 13 | 10 | 16 | 10 | 19 |
| Tm-M-093 | 14 | 12 | 23 | 28 | 15 | 14 | 13,15 | 12 | 10 | 12 | 20 | 11 | 12 | 14 | 9 | 21 |
| Tm-M-095 | 15 | 14 | 23 | 30 | 16 | 14 | 13,17 | 12 | 10 | 12 | 21 | 11 | 12 | 14 | 9 | 21 |
| Tm-M-096 | 16 | 12 | 21 | 30 | 16 | 15 | 14,15 | 13 | 10 | 11 | 21 | 11 | 11 | 16 | 11 | 24 |
| Tm-M-097 | 15 | 14 | 22 | 29 | 15 | 14 | 11,12 | 13 | 10 | 12 | 20 | 12 | 12 | 15 | 11 | 19 |
| Tm-M-098 | 16 | 12 | 24 | 31 | 15 | 12 | 17,17 | 13 | 10 | 13 | 22 | 11 | 11 | 14 | 10 | 20 |
| Tm-M-099 | 15 | 14 | 23 | 30 | 15 | 15 | 13,17 | 11 | 10 | 12 | 22 | 13 | 10 | 16 | 10 | 19 |
| Tm-M-100 | 15 | 12 | 23 | 30 | 17 | 14 | 14,15 | 14 | 10 | 11 | 22 | 11 | 12 | 15 | 10 | 21 |
| Tm-M-101 | 16 | 13 | 24 | 30 | 16 | 16 | 11,14 | 13 | 11 | 10 | 23 | 11 | 13 | 14 | 12 | 20 |
| TM-M-103 | 15 | 13 | 23 | 31 | 15 | 13 | 16,17 | 12 | 10 | 11 | 20 | 13 | 11 | 14 | 9 | 19 |
| Tm-M-104 | 15 | 13 | 22 | 32 | 20.2 | 14 | 12,20 | 12 | 10 | 12 | 20 | 11 | 12 | 14 | 10 | 20 |
| Tm-M-105 | 16 | 13 | 24 | 30 | 16 | 17 | 9,11 | 13 | 11 | 10 | 24 | 11 | 12 | 14 | 11 | 20 |
| Tm-M-106 | 16 | 12 | 24 | 29 | 15 | 13 | 15,17 | 13 | 10 | 12 | 21 | 11 | 12 | 14 | 10 | 20 |
| Tm-M-107 | 15 | 13 | 25 | 29 | 17 | 17 | 9,11 | 13 | 10 | 10 | 23 | 11 | 12 | 14 | 11 | 20 |
| Tm-M-108 | 15 | 12 | 22 | 28 | 14 | 14 | 14,15 | 13 | 11 | 11 | 22 | 12 | 11 | 15 | 10 | 21 |
| Tm-M-109 | 16 | 14 | 22 | 30 | 18 | 15 | 14,17 | 12 | 11 | 13 | 21 | 11 | 12 | 14 | 9 | 19 |
| Tm-M-110 | 15 | 14 | 23 | 32 | 15 | 15 | 13,16 | 12 | 10 | 11 | 23 | 11 | 11 | 14 | 9 | 20 |
| Yz-M-001 | 15 | 14 | 23 | 30 | 18.2 | 14 | 12,18 | 12 | 10 | 11 | 22 | 11 | 11 | 14 | 10 | 21,22 |
| Yz-M-002 | 15 | 13 | 23 | 30 | 15 | 14 | 14,14 | 14 | 10 | 12 | 20 | 11 | 12 | 14 | 9 | 20 |
| Yz-M-003 | 16 | 13 | 24 | 29 | 18 | 14 | 11,14 | 12 | 12 | 12 | 23 | 14 | 12 | 15 | 12 | 19 |
| Yz-M-004 | 15 | 13 | 25 | 31 | 18 | 14 | 15,15 | 13 | 10 | 14 | 21 | 11 | 10 | 14 | 10 | 20 |
| Yz-M-006 | 15 | 13 | 25 | 31 | 18 | 14 | 15,15 | 13 | 10 | 14 | 21 | 11 | 10 | 14 | 10 | 20 |
| Yz-M-007 | 15 | 13 | 23 | 30 | 15 | 14 | 14,20 | 14 | 10 | 12 | 20 | 11 | 12 | 14 | 9 | 20 |
| Yz-M-008 | 15 | 13 | 23 | 30 | 15 | 14 | 14,20 | 14 | 10 | 12 | 20 | 11 | 12 | 14 | 9 | 20 |
| Yz-M-009 | 14 | 14 | 23 | 30 | 17 | 14 | 17,17 | 12 | 10 | 11 | 24 | 14 | 12 | 16 | 10 | 19 |
| Yz-M-010 | 14 | 14 | 23 | 30 | 17 | 14 | 17,17 | 12 | 10 | 11 | 24 | 14 | 12 | 16 | 10 | 19 |
| Yz-M-011 | 15 | 13 | 23 | 30 | 15 | 15 | 12,12 | 13 | 11 | 12 | 23 | 13 | 12 | 15 | 11 | 19 |
| Yz-M-012 | 15 | 13 | 23 | 30 | 15 | 15 | 12,12 | 13 | 11 | 12 | 23 | 13 | 12 | 15 | 11 | 19 |
| Yz-M-013 | 15 | 13 | 23 | 30 | 15 | 15 | 12,12 | 13 | 11 | 12 | 23 | 13 | 12 | 15 | 11 | 19 |
| Yz-M-014 | 14 | 14 | 23 | 30 | 18 | 14 | 17,17 | 12 | 10 | 11 | 24 | 14 | 12 | 16 | 10 | 19 |
| Yz-M-015 | 14 | 14 | 23 | 30 | 18 | 14 | 17,17 | 12 | 10 | 11 | 24 | 14 | 12 | 16 | 10 | 19 |
| Yz-M-016 | 14 | 14 | 23 | 30 | 17 | 14 | 17,17 | 12 | 10 | 11 | 24 | 14 | 12 | 16 | 10 | 19 |
| Yz-M-017 | 15 | 13 | 24 | 28 | 18 | 14 | 11,14 | 12 | 12 | 14 | 23 | 14 | 12 | 15 | 12 | 19 |
| Yz-M-018 | 16 | 13 | 24 | 28 | 18 | 14 | 11,14 | 12 | 11 | 12 | 23 | 14 | 12 | 15 | 12 | 19 |
| Yz-M-019 | 16 | 13 | 24 | 28 | 18 | 14 | 11,14 | 12 | 11 | 12 | 23 | 14 | 12 | 15 | 12 | 19 |
| Yz-M-020 | 16 | 13 | 24 | 28 | 18 | 14 | 11,14 | 12 | 11 | 12 | 23 | 14 | 12 | 15 | 12 | 19 |
| Yz-M-023 | 18 | 12 | 22 | 29 | 17 | 14,16 | 13,16 | 14 | 10 | 11 | 20 | 11 | 11 | 17 | 10 | 21 |
| Yz-M-024 | 14 | 14 | 23 | 30 | 18 | 14 | 17,18 | 12 | 10 | 11 | 24 | 14 | 12 | 16 | 10 | 19 |
| Yz-M-025 | 16 | 13 | 24 | 30 | 15 | 13 | 16,18 | 13 | 10 | 11 | 20 | 11 | 12 | 14 | 10 | 20 |
| Yz-M-026 | 16 | 13 | 24 | 28 | 18 | 14 | 11,14 | 12 | 11 | 12 | 23 | 14 | 12 | 15 | 12 | 19 |
| Yz-M-027 | 15 | 13 | 23 | 29 | 17 | 14 | 12,16 | 12 | 10 | 11 | 23 | 11 | 10 | 15 | 9 | 20 |
| Yz-M-028 | 15 | 13 | 24 | 31 | 18 | 14 | 15,15 | 13 | 10 | 14 | 21 | 11 | 10 | 14 | 10 | 20 |
| Yz-M-029 | 16 | 13 | 24 | 29 | 18 | 14 | 11,14 | 12 | 12 | 12 | 23 | 14 | 12 | 15 | 12 | 19 |
| Yz-M-030 | 15 | 13 | 23 | 29 | 17 | 14 | 12,16 | 12 | 10 | 11 | 23 | 11 | 10 | 15 | 9 | 20 |
| Yz-M-031 | 16 | 13 | 25 | 29 | 17 | 14 | 15,17 | 13 | 10 | 11 | 21 | 13 | 11 | 14 | 10 | 19 |
| Yz-M-032 | 16 | 13 | 24 | 30 | 15 | 13 | 16,18 | 13 | 10 | 11 | 20 | 11 | 12 | 14 | 10 | 20 |
| Yz-M-033 | 16 | 13 | 24 | 28 | 18 | 14 | 11,13 | 12 | 11 | 14 | 23 | 14 | 12 | 15 | 12 | 19 |
| Yz-M-034 | 16 | 13 | 25 | 29 | 17 | 14 | 15,17 | 13 | 10 | 11 | 21 | 13 | 11 | 14 | 10 | 19 |
| Yz-M-035 | 14 | 14 | 22 | 30 | 18 | 14 | 17,17 | 12 | 10 | 11 | 24 | 14 | 12 | 16 | 10 | 19 |
| Yz-M-036 | 15 | 13 | 23 | 29 | 16 | 14 | 14,16 | 12 | 10 | 11 | 21 | 11 | 11 | 14 | 9 | 20 |
| Yz-M-037 | 15 | 12 | 22 | 29 | 15 | 14 | 15,16 | 13 | 10 | 12 | 21 | 11 | 11 | 17 | 10 | 19,20 |
| Yz-M-038 | 15 | 13 | 23 | 30 | 17 | 15 | 12,12 | 13 | 11 | 12 | 23 | 13 | 12 | 15 | 11 | 19 |
| Yz-M-039 | 16 | 13 | 25 | 28 | 18 | 14 | 11,11 | 12 | 11 | 12 | 23 | 14 | 12 | 15 | 12 | 19 |
| Yz-M-040 | 15 | 12 | 22 | 30 | 15 | 14 | 15,16 | 13 | 10 | 12 | 21 | 11 | 11 | 17 | 10 | 19,20 |
| Yz-M-041 | 14 | 14 | 22 | 30 | 18 | 14 | 17,17 | 12 | 10 | 11 | 24 | 14 | 12 | 16 | 10 | 19 |
| Yz-M-042 | 16 | 13 | 25 | 29 | 17 | 14 | 15,17 | 13 | 10 | 11 | 21 | 13 | 11 | 14 | 10 | 19 |
| Yz-M-043 | 16 | 13 | 24 | 30 | 15 | 13 | 16,18 | 13 | 10 | 11 | 20 | 11 | 12 | 14 | 10 | 20 |
| Yz-M-044 | 14 | 12 | 23 | 29 | 18.2 | 14 | 12,20 | 12 | 10 | 12 | 22 | 11 | 11 | 14 | 10 | 20 |
| Yz-M-045 | 15 | 13 | 23 | 28 | 18 | 14 | 11,14 | 12 | 12 | 12 | 23 | 14 | 12 | 15 | 12 | 19 |
| Yz-M-046 | 18 | 13 | 21 | 30.3 | 16 | 16 | 17,18 | 14 | 10 | 13 | 21 | 11 | 11 | 14 | 11 | 21 |
| Yz-M-047 | 16 | 13 | 24 | 30 | 15 | 13 | 16,18 | 13 | 10 | 11 | 20 | 11 | 12 | 14 | 10 | 20 |
| Yz-M-048 | 18 | 13 | 21 | 30.3 | 16 | 16 | 17,18 | 14 | 10 | 13 | 21 | 11 | 11 | 14 | 11 | 21 |
| Yz-M-049 | 15 | 14 | 23 | 30 | 18.2 | 14 | 12,18 | 12 | 10 | 11 | 22 | 11 | 11 | 14 | 10 | 21,22 |
| Yz-M-050 | 18 | 13 | 21 | 30.3 | 16 | 16 | 17,18 | 14 | 10 | 13 | 21 | 11 | 11 | 14 | 11 | 21 |
| Yz-M-051 | 15 | 13 | 23 | 30 | 17 | 15 | 12,12 | 13 | 11 | 12 | 23 | 13 | 12 | 15 | 11 | 19 |
| Yz-M-052 | 15 | 13 | 23 | 30 | 15 | 14 | 14,20 | 14 | 10 | 12 | 20 | 11 | 12 | 14 | 9 | 20 |
| Yz-M-053 | 15 | 13 | 23 | 30 | 15 | 14 | 14,20 | 14 | 10 | 12 | 20 | 11 | 12 | 14 | 9 | 20 |
| Yz-M-054 | 16 | 14 | 25 | 30 | 17 | 14 | 15,17 | 13 | 10 | 11 | 21 | 13 | 11 | 14 | 10 | 19 |
| Yz-M-055 | 16 | 14 | 25 | 30 | 17 | 14 | 15,17 | 13 | 10 | 11 | 21 | 13 | 11 | 14 | 10 | 19 |
| Yz-M-056 | 15 | 12 | 22 | 29 | 15 | 15 | 15,16 | 13 | 10 | 12 | 21 | 11 | 11 | 17 | 10 | 19,20 |
| Yz-M-057 | 15 | 12 | 22 | 29 | 15 | 15 | 15,16 | 13 | 10 | 12 | 21 | 11 | 11 | 17 | 10 | 19,20 |
| Yz-M-058 | 15 | 12 | 22 | 29 | 15 | 15 | 15,16 | 13 | 10 | 11 | 21 | 11 | 11 | 17 | 10 | 19,20 |
| Yz-M-059 | 15 | 13 | 23 | 29 | 17 | 14 | 12,16 | 12 | 10 | 11 | 23 | 11 | 10 | 15 | 9 | 20 |
| Yz-M-060 | 15 | 13 | 23 | 30 | 15 | 14 | 14,20 | 14 | 10 | 12 | 20 | 11 | 12 | 14 | 9 | 20 |
| Yz-M-061 | 14 | 14 | 23 | 30 | 18 | 15 | 17,17 | 12 | 10 | 11 | 24 | 14 | 12 | 16 | 10 | 19 |
| Yz-M-062 | 16 | 13 | 24 | 28 | 18 | 14 | 11,14 | 12 | 11 | 12 | 23 | 14 | 12 | 15 | 12 | 19 |
| Yz-M-063 | 16 | 13 | 22 | 29 | 14 | 14 | 13,16 | 12 | 10 | 11 | 21 | 11 | 11 | 15 | 9 | 21 |
| Yz-M-064 | 14 | 14 | 23 | 31 | 18.2 | 14 | 13,19 | 12 | 11 | 11 | 21 | 11 | 12 | 14 | 10 | 20 |
| Yz-M-065 | 16 | 13 | 22 | 29 | 14 | 14 | 13,16 | 12 | 10 | 11 | 21 | 11 | 11 | 15 | 9 | 21 |
| Yz-M-066 | 15 | 13 | 24 | 30 | 15 | 15 | 11,15 | 13 | 11 | 10 | 24 | 11 | 12 | 14 | 11 | 20 |
| Yz-M-067 | 16 | 13 | 25 | 29 | 17 | 14 | 15,17 | 13 | 10 | 11 | 21 | 13 | 11 | 14 | 10 | 19 |
| Yz-M-068 | 16 | 13 | 24 | 30 | 15 | 13 | 16,18 | 13 | 10 | 11 | 20 | 11 | 12 | 14 | 10 | 20 |
| Yz-M-069 | 15 | 13 | 23 | 29 | 15 | 14 | 12,20 | 12 | 10 | 11 | 21 | 11 | 13 | 15 | 9 | 19 |
| Yz-M-070 | 15 | 13 | 24 | 30 | 15 | 15 | 11,15 | 13 | 11 | 10 | 24 | 11 | 12 | 14 | 11 | 20 |
| Yz-M-071 | 15 | 13 | 24 | 30 | 15 | 15 | 11,15 | 13 | 11 | 10 | 24 | 11 | 12 | 14 | 11 | 20 |
| Yz-M-072 | 16 | 13 | 22 | 29 | 14 | 14 | 13,16 | 12 | 10 | 11 | 21 | 11 | 11 | 15 | 9 | 21 |
| Yz-M-073 | 14 | 12 | 22 | 29 | 17 | 16 | 15,16 | 14 | 10 | 12 | 20 | 10 | 12 | 16 | 10 | 20 |
| Yz-M-074 | 14 | 12 | 22 | 29 | 17 | 16 | 15,16 | 14 | 10 | 12 | 20 | 10 | 12 | 16 | 10 | 20 |
| Yz-M-075 | 14 | 12 | 22 | 29 | 17 | 16 | 15,16 | 14 | 10 | 12 | 20 | 10 | 12 | 16 | 10 | 20 |
| Yz-M-076 | 16 | 13 | 25 | 30 | 16 | 16 | 11,14 | 13 | 11 | 10 | 23 | 11 | 13 | 14 | 11 | 20 |
| Yz-M-077 | 15 | 13 | 24 | 29 | 18.2 | 14 | 18,19 | 12 | 10 | 12 | 21 | 11 | 11 | 14 | 10 | 19 |
| Yz-M-078 | 15 | 13 | 24 | 29 | 18.2 | 14 | 18,19 | 12 | 10 | 12 | 21 | 11 | 11 | 14 | 10 | 19 |
| Yz-M-079 | 16 | 13 | 25 | 30 | 16 | 16 | 11,14 | 13 | 11 | 10 | 23 | 11 | 13 | 14 | 11 | 20 |
| Yz-M-080 | 16 | 13 | 24 | 30 | 15 | 13 | 16,18 | 13 | 10 | 11 | 20 | 11 | 12 | 14 | 10 | 20 |
| Yz-M-081 | 16 | 13 | 24 | 28 | 17 | 14 | 11,14 | 12 | 12 | 12 | 23 | 14 | 12 | 15 | 12 | 19 |
| Yz-M-082 | 16 | 13 | 25 | 30 | 16 | 16 | 11,14 | 13 | 11 | 10 | 23 | 11 | 13 | 14 | 11 | 20 |
| Yz-M-083 | 15 | 13 | 23 | 29 | 16 | 14 | 14,16 | 12 | 10 | 11 | 21 | 11 | 11 | 14 | 9 | 20 |
| Yz-M-084 | 15 | 13 | 23 | 29 | 16 | 14 | 14,16 | 12 | 10 | 11 | 21 | 11 | 11 | 14 | 9 | 20 |
| Yz-M-085 | 16 | 13 | 24 | 28 | 17 | 14 | 11,14 | 12 | 12 | 12 | 23 | 14 | 12 | 15 | 12 | 19 |
| Yz-M-086 | 16 | 13 | 25 | 30 | 16 | 16 | 11,14 | 13 | 11 | 10 | 23 | 11 | 13 | 14 | 11 | 20 |
| Yz-M-087 | 16 | 13 | 25 | 30 | 16 | 16 | 11,14 | 13 | 11 | 10 | 23 | 11 | 13 | 14 | 11 | 20 |
| Yz-M-088 | 16 | 13 | 24 | 28 | 17 | 14 | 11,14 | 12 | 12 | 12 | 23 | 14 | 12 | 15 | 12 | 19 |
| Yz-M-089 | 15 | 13 | 23 | 29 | 16 | 14 | 14,16 | 12 | 10 | 11 | 21 | 11 | 11 | 14 | 9 | 20 |
| Yz-M-090 | 16 | 13 | 24 | 30 | 15 | 13 | 16,18 | 13 | 10 | 11 | 20 | 11 | 12 | 14 | 10 | 20 |
| Yz-M-091 | 16 | 13 | 24 | 28 | 17 | 14 | 11,14 | 12 | 12 | 12 | 23 |  | 12 | 15 | 12 | 19 |
| Yz-M-092 | 15 | 14 | 24 | 32 | 17 | 14 | 14,15 | 12 | 10 | 13 | 24 | 11 | 12 | 15 | 9 | 20 |
| Yz-M-093 | 16 | 13 | 24 | 30 | 15 | 13 | 16,18 | 13 | 10 | 11 | 20 | 11 | 12 | 14 | 10 | 20 |
| Yz-M-094 | 15 | 13 | 24 | 30 | 16 | 15 | 11,15 | 13 | 11 | 10 | 24 | 11 | 12 | 15 | 8 |  |
| Yz-M-095 | 14 | 14 | 23 | 30 | 18 | 14 | 17,17 | 12 | 10 | 11 | 24 | 14 | 12 | 15 | 10 | 19 |
| Yz-M-096 | 13 | 13 | 24 | 29 | 14 | 15 | 12,14 | 13 | 10 | 12 | 24 | 13 | 12 | 14 | 12 | 18 |
| Yz-M-097 | 16 | 13 | 23 | 29 | 16 | 16 | 13,16 | 12 | 10 | 10 | 22 | 11 | 11 | 15 | 9 | 20 |
| Yz-M-098 | 15 | 12 | 23 | 29 | 19 | 14 | 13,17 | 12 | 10 | 12 | 21 | 11 | 11 | 15 | 9 | 19 |
| Yz-M-099 | 15 | 14 | 23 | 32 | 16 | 14 | 14,20 | 10 | 10 | 11 | 21 | 11 | 13 | 15 | 9 | 19 |
| Yz-M-100 | 15 | 12 | 21 | 28 | 17 | 14 | 12,14 | 14 | 10 | 11 | 21 | 11 | 12 | 16 | 10 | 21 |
| Yz-M-101 | 15 | 13 | 23 | 29 | 15 | 14 | 12,20 | 12 | 10 | 11 | 21 | 11 | 13 | 15 | 9 | 19 |
| Yz-M-102 | 15 | 12 | 21 | 28 | 17 | 14 | 12,15 | 14 | 10 | 11 | 21 | 11 | 12 | 16 | 10 | 21 |
| Yz-M-103 | 13 | 13 | 24 | 29 | 14 | 15 | 12,14 | 13 | 10 | 12 | 24 | 13 | 12 | 14 | 12 | 18 |
| Yz-M-104 | 16 | 13 | 25 | 29 | 17 | 14 | 15,17 | 13 | 10 | 11 | 21 | 13 | 11 | 14 | 10 | 19 |
| Yz-M-105 | 16 | 13 | 24 | 28 | 18 | 14 | 11,13 | 12 | 11 | 13 | 23 | 14 | 12 | 15 | 12 | 19 |
| Yz-M-106 | 16 | 13 | 24 | 29 | 15 | 15 | 12,17 | 12 | 10 | 12 | 22 | 14 | 11 | 16 | 10 | 21 |
| Yz-M-107 | 15 | 14 | 23 | 30 | 18.2 | 14 | 12,18 | 12 | 10 | 11 | 22 | 11 | 11 | 14 | 10 | 21,22 |
| Yz-M-108 | 15 | 14 | 24 | 32 | 17 | 14 | 14,15 | 12 | 10 | 13 | 24 | 11 | 12 | 15 | 9 | 20 |
| Yz-M-109 | 14 | 14 | 22 | 30 | 18 | 14 | 17,17 | 12 | 10 | 11 | 24 | 14 | 12 | 16 | 10 | 19 |

Abbreviations: M, Male; Ar, Arab; Kr, Kurdish; Sy, Syriac; Tm, Turkmen; Yz, Yazidi

**Overall dataset statistics**

| Number of different haplotypes: 360 | Number of haplotypes shared by 2 individuals: 50 |
| --- | --- |
| Number of unique haplotypes: 280 | Number of haplotypes shared by 3 individuals: 17 |
| Unique Haplotypes (UH) for the entire dataset: 56.0 % | Number of haplotypes shared by 4 individuals: 5 |
| Discrimination Capacity (DC) for the entire dataset: 72.0 % | Number of haplotypes shared by 5 individuals: 5 |
| Haplotype Diversity (HD) for the entire dataset 0.9979 | Number of haplotypes shared by 7 individuals: 1 |
|  | Number of haplotypes shared by 8 individuals: 1 |
|  | Number of haplotypes shared by 9 individuals: 1 |
