# Supplementary material for: A glimpse at the intricate mosaic of ethnicities from Mesopotamia: Paternal lineages of the Northern Iraqi Arabs, Kurds, Syriacs, Turkmens and Yazidis
Source: PLoS One. 2017 Nov 3;12(11):e0187408. doi: 10.1371/journal.pone.0187408 (PMC5669434; doi:10.1371/journal.pone.0187408)
Supplement: S3 Table — (DOC) [file pone.0187408.s003.doc]

| **Ar-M-001** | E1b1b | 78 | 100.0 |  | E1b1b | 47 | 100.0 | Yes |
| --- | --- | --- | --- | --- | --- | --- | --- | --- |
| **Ar-M-002** | J2a1b | 66 | 99.9 |  | J2a1 Z6065 | 53 | 96.4 | Yes |
| **Ar-M-003** | J1 | 68 | 100.0 |  | J1a3 Z1828 | 51 | 86.5 | Yes |
| **Ar-M-004** | G2a | 47 | 100.0 |  | G2a2b1 M406 | 34 | 54.7 | Yes |
| **Ar-M-005** | J1 | 42 | 95.5 |  | J1a2a1a2 P58 | 45 | 98.5 | Yes |
| **Ar-M-006** | J1 | 83 | 99.9 |  | J1a2a1a2 P58 | 68 | 99.6 | Yes |
| **Ar-M-007** | T | 67 | 100.0 |  | T>PF5633 | 45 | 99.0 | Yes |
| **Ar-M-008** | R1a | 51 | 100.0 |  | R1a | 36 | 100.0 | Yes |
| **Ar-M-009** | Q | 36 | 98.2 |  | Q L275>> L245 | 25 | 95.7 | Yes |
| **Ar-M-010** | H | 40 | 78.3 |  | J2b1-M205 | 59 | 100.0 | No |
| **Ar-M-011** | J1 | 66 | 99.8 |  | J1a2a1a2 P58 | 44 | 84.9 | Yes |
| **Ar-M-012** | H | 27 | 97.8 |  | E1b1b | 17 | 14.5 | No |
| **Ar-M-013** | **J2a1b** | 25 | 41.7 |  | J2a2 PF5008 | 28 | 30.2 | No, but only different sub-clade |
| **Ar-M-014** | I2b1 | 47 | 100.0 |  | I2a2a | 34 | 99.9 | No, but only different sub-clade |
| **Ar-M-015** | T | 63 | 100.0 |  | T>PF5633 | 43 | 97.2 | Yes |
| **Ar-M-016** | J1 | 53 | 99.9 |  | J1a2a1a2 P58 | 59 | 100.0 | Yes |
| **Ar-M-017** | J1 | 46 | 99.8 |  | J1a2a1a2 P58 | 44 | 99.6 | Yes |
| **Ar-M-018** | I2b1 | 47 | 100.0 |  | I2a2a | 34 | 99.9 | No, but only different sub-clade |
| **Ar-M-019** | J2b | 28 | 100.0 |  | J2b2 M241 | 20 | 30.6 | Yes |
| **Ar-M-020** | Q | 36 | 98.2 |  | Q L275>> L245 | 25 | 95.7 | Yes |
| **Ar-M-021** | J1 | 66 | 99.8 |  | J1a2a1a2 P58 | 44 | 84.9 | Yes |
| **Ar-M-022** | T | 62 | 100.0 |  | T>PF5633 | 40 | 99.6 | Yes |
| **Ar-M-023** | J1 | 63 | 100.0 |  | J1a2a1a2 P58 | 70 | 100.0 | Yes |
| **Ar-M-024** | G2a | 74 | 100.0 |  | G2a2b2a1a U1 | 44 | 47.6 | Yes |
| **Ar-M-025** | L | 41 | 100.0 |  | L1a | 26 | 100.0 | Yes |
| **Ar-M-026** | T | 92 | 100.0 |  | T>PF5633 | 67 | 100.0 | Yes |
| **Ar-M-028** | J2a1 x J2a1b/h | 76 | 84.5 |  | J2a1 Z7671 | 35 | 56.2 | Yes |
| **Ar-M-029** | R1a | 64 | 100.0 |  | R1a | 53 | 100.0 | Yes |
| **Ar-M-030** | R1a | 67 | 100.0 |  | R1a | 54 | 100.0 | Yes |
| **Ar-M-031** | J1 | 52 | 99.8 |  | J1a2a1a2 P58 | 54 | 99.0 | Yes |
| **Ar-M-032** | J2a1b | 69 | 88.5 |  | J2a1 M319 | 40 | 38.4 | Yes |
| **Ar-M-033** | J1 | 62 | 100.0 |  | J1a2a1a2 P58 | 55 | 99.8 | Yes |
| **Ar-M-034** | E1b1b | 49 | 99.9 |  | E1b1b | 35 | 99.4 | Yes |
| **Ar-M-035** | J1 | 49 | 100.0 |  | J1a2a1a2 P58 | 54 | 99.9 | Yes |
| **Ar-M-036** | J2a1 x J2a1b/h | 37 | 82.4 |  | J2b2 M241 | 23 | 20.3 | No, but only different sub-clade |
| **Ar-M-037** | T | 87 | 100.0 |  | T>PF5633 | 63 | 100.0 | Yes |
| **Ar-M-038** | R1a | 42 | 100.0 |  | R1a | 34 | 100.0 | Yes |
| **Ar-M-039** | J2a1h | 81 | 97.9 |  | J2a1 Z7700 | 42 | 61.9 | Yes |
| **Ar-M-040** | L | 80 | 100.0 |  | L1b | 64 | 100.0 | Yes |
| **Ar-M-041** | J1 | 65 | 100.0 |  | J1a2a1a2 P58 | 76 | 100.0 | Yes |
| **Ar-M-043** | J1 | 54 | 100.0 |  | J1a2a1a2 P58 | 55 | 100.0 | Yes |
| **Ar-M-044** | J1 | 57 | 99.9 |  | J1a2a1a2 P58 | 62 | 99.9 | Yes |
| **Ar-M-045** | T | 87 | 100.0 |  | T>PF5633 | 56 | 99.4 | Yes |
| **Ar-M-046** | J2a1h | 80 | 100.0 |  | J2a1 Z387 | 61 | 99.9 | Yes |
| **Ar-M-047** | J1 | 64 | 99.9 |  | J1a2a1a2 P58 | 68 | 100.0 | Yes |
| **Ar-M-048** | R1a | 81 | 100.0 |  | R1a | 61 | 100.0 | Yes |
| **Ar-M-050** | J1 | 27 | 99.6 |  | J1a3 Z1828 | 25 | 28.7 | Yes |
| **Ar-M-051** | R1a | 67 | 100.0 |  | R1a | 38 | 100.0 | Yes |
| **Ar-M-052** | R1a | 81 | 100.0 |  | R1a | 61 | 100.0 | Yes |
| **Ar-M-053** | J1 | 61 | 99.6 |  | J1a2a1a2 P58 | 65 | 100.0 | Yes |
| **Ar-M-054** | J1 | 29 | 54.6 |  | J1a2a1a2 P58 | 50 | 99.9 | Yes |
| **Ar-M-055** | J1 | 45 | 81.8 |  | J1a3 Z1828 | 52 | 94.7 | Yes |
| **Ar-M-056** | J1 | 50 | 100.0 |  | J1a2a1a2 P58 | 52 | 99.9 | Yes |
| **Ar-M-057** | R1a | 80 | 100.0 |  | R1a | 55 | 100.0 | Yes |
| **Ar-M-058** | Q | 36 | 98.2 |  | Q L275>> L245 | 25 | 95.7 | Yes |
| **Ar-M-059** | R1b | 29 | 100.0 |  | R1b | 22 | 98.0 | Yes |
| **Ar-M-060** | J1 | 63 | 99.9 |  | J1a2a1a2 P58 | 37 | 98.5 | Yes |
| **Ar-M-061** | J1 | 46 | 91.6 |  | J1a3 Z1828 | 48 | 93.9 | Yes |
| **Ar-M-062** | R1a | 56 | 100.0 |  | R1a | 38 | 100.0 | Yes |
| **Ar-M-063** | T | 77 | 100.0 |  | T>PF5633 | 40 | 81.6 | Yes |
| **Ar-M-064** | J2b | 59 | 100.0 |  | J2b2-Z2456 | 64 | 99.6 | Yes |
| **Ar-M-065** | J1 | 46 | 99.6 |  | J1a2a1a2 P58 | 30 | 88.2 | Yes |
| **Ar-M-066** | E1b1b | 65 | 100.0 |  | E1b1b | 38 | 100.0 | Yes |
| **Ar-M-067** | J2a1 x J2a1b/h | 49 | 100.0 |  | J2a1 PF5191 | 30 | 47.5 | Yes |
| **Ar-M-068** | E1b1b | 50 | 100.0 |  | E1b1b | 37 | 100.0 | Yes |
| **Ar-M-069** | T | 56 | 100.0 |  | T>PF5633 | 30 | 96.0 | Yes |
| **Ar-M-070** | J1 | 50 | 100.0 |  | J1a2a1a2 P58 | 52 | 99.9 | Yes |
| **Ar-M-071** | J1 | 56 | 99.8 |  | J1a2a1a2 P58 | 57 | 99.6 | Yes |
| **Ar-M-072** | L | 45 | 100.0 |  | L1a | 31 | 100.0 | Yes |
| **Ar-M-073** | J1 | 50 | 100.0 |  | J1a2a1a2 P58 | 52 | 99.9 | Yes |
| **Ar-M-074** | J2a1b | 70 | 50.8 |  | J2a1 L26>Z500 | 49 | 100.0 | Yes |
| **Ar-M-075** | J1 | 70 | 99.9 |  | J1a2a1a2 P58 | 65 | 100.0 | Yes |
| **Ar-M-076** | J1 | 67 | 99.9 |  | J1a2a1a2 P58 | 74 | 100.0 | Yes |
| **Ar-M-077** | R1a | 55 | 100.0 |  | R1a | 44 | 100.0 | Yes |
| **Ar-M-078** | E1b1b | 45 | 87.6 |  | E1b1b | 34 | 99.2 | Yes |
| **Ar-M-079** | J2a1b | 63 | 72.8 |  | J2a1 L26>Z500 | 36 | 56.0 | Yes |
| **Ar-M-080** | J1 | 43 | 99.2 |  | J1a3 Z1828 | 39 | 60.2 | Yes |
| **Ar-M-082** | J1 | 35 | 97.7 |  | J1a2a1a2 P58 | 29 | 91.6 | Yes |
| **Ar-M-083** | R1b | 36 | 100.0 |  | R1b | 27 | 99.8 | Yes |
| **Ar-M-084** | R1a | 84 | 100.0 |  | R1a | 64 | 100.0 | Yes |
| **Ar-M-085** | G2a | 60 | 100.0 |  | G2a1-L293 | 42 | 88.3 | Yes |
| **Ar-M-086** | E1b1b | 71 | 100.0 |  | E1b1b | 35 | 99.4 | Yes |
| **Ar-M-087** | J1 | 56 | 100.0 |  | J1a2a1a2 P58 | 58 | 100.0 | Yes |
| **Ar-M-088** | J1 | 64 | 99.9 |  | J1a2a1a2 P58 | 68 | 100.0 | Yes |
| **Ar-M-089** | T | 46 | 99.7 |  | T>PF5633 | 32 | 98.4 | Yes |
| **Ar-M-090** | R1a | 76 | 100.0 |  | R1a | 58 | 100.0 | Yes |
| **Ar-M-091** | J1 | 27 | 99.6 |  | J1a3 Z1828 | 25 | 28.7 | Yes |
| **Ar-M-092** | L | 41 | 100.0 |  | L1a | 26 | 100.0 | Yes |
| **Ar-M-093** | J1 | 57 | 99.9 |  | J1a2a1a2 P58 | 61 | 99.8 | Yes |
| **Ar-M-095** | E1b1b | 49 | 97.4 |  | E1b1b | 32 | 98.4 | Yes |
| **Ar-M-096** | J1 | 65 | 100.0 |  | J1a2a1a2 P58 | 76 | 100.0 | Yes |
| **Ar-M-097** | E1b1b | 51 | 100.0 |  | E1b1b | 34 | 99.3 | Yes |
| **Ar-M-098** | J1 | 70 | 99.9 |  | J1a2a1a2 P58 | 65 | 100.0 | Yes |
| **Ar-M-099** | J1 | 64 | 99.9 |  | J1a2a1a2 P58 | 68 | 100.0 | Yes |
| **Ar-M-101** | Q | 48 | 98.5 |  | Q M346>> Z780 | 28 | 57.9 | Yes |
| **Ar-M-102** | R1b | 62 | 100.0 |  | R1b | 53 | 100.0 | Yes |
| **Ar-M-103** | J2a1 x J2a1b/h | 49 | 94.8 |  | J2a1 Z7700 | 32 | 40.2 | Yes |
| **Ar-M-104** | R1a | 81 | 100.0 |  | R1a | 61 | 100.0 | Yes |
| **Ar-M-105** | Q | 67 | 100.0 |  | Q L275>> L245 | 47 | 100.0 | Yes |
| **Ar-M-106** | J2a1b | 62 | 98.5 |  | J2a1 PF5191 | 28 | 19.3 | Yes |
| **Ar-M-107** | J1 | 57 | 99.9 |  | J1a2a1a2 P58 | 62 | 99.9 | Yes |
| **Ar-M-108** | J1 | 41 | 99.6 |  | J1a >> PF7257 | 38 | 52.6 | Yes |
| **Kr-M-001** | J2a1h | 52 | 100.0 |  | J2a1 Z387 | 39 | 84.1 | Yes |
| **Kr-M-003** | J2a1h | 75 | 100.0 |  | J2a1 Z387 | 43 | 92.1 | Yes |
| **Kr-M-004** | **L** | 24 | 76.1 |  | R2 | 43 | 100.0 | No |
| **Kr-M-005** | J2a1b | 35 | 77.7 |  | J2a1 PF5191 | 22 | 12.4 | Yes |
| **Kr-M-006** | J2a1b | 77 | 70.5 |  | J2a1 Z7700 | 47 | 32.5 | Yes |
| **Kr-M-007** | E1b1a | 25 | 83.1 |  | E1b1b | 22 | 45.6 | Yes |
| **Kr-M-008** | **Q** | 9 | 73.8 |  | R2 | 13 | 0.2 | No |
| **Kr-M-009** | Q | 63 | 100.0 |  | Q L275>> L245 | 60 | 100.0 | Yes |
| **Kr-M-010** | R1a | 52 | 100.0 |  | R1a | 34 | 100.0 | Yes |
| **Kr-M-011** | E1b1b | 51 | 83.2 |  | E1b1b | 34 | 99.2 | Yes |
| **Kr-M-012** | L | 54 | 100.0 |  | L1c | 23 | 93.3 | Yes |
| **Kr-M-013** | **Q** | 24 | 100.0 |  | R2 | 32 | 100.0 | No |
| **Kr-M-014** | J1 | 63 | 100.0 |  | J1a2a1a2 P58 | 70 | 100.0 | Yes |
| **Kr-M-015** | R1a | 45 | 100.0 |  | R1a | 32 | 99.9 | Yes |
| **Kr-M-016** | R1b | 41 | 100.0 |  | R1b | 31 | 100.0 | Yes |
| **Kr-M-018** | R1b | 45 | 100.0 |  | R1b | 35 | 100.0 | Yes |
| **Kr-M-019** | I2a1 | 56 | 82.5 |  | I2c2 Y16419 | 42 | 60.6 | No, but only different sub-clade |
| **Kr-M-020** | R1a | 67 | 100.0 |  | R1a | 45 | 100.0 | Yes |
| **Kr-M-021** | Q | 63 | 100.0 |  | Q L275>> L245 | 60 | 100.0 | Yes |
| **Kr-M-022** | J2a1 x J2a1b/h | 54 | 54.3 |  | J2a1 PF5191 | 39 | 47.5 | Yes |
| **Kr-M-023** | J1 | 57 | 97.4 |  | J1a2a1a2 P58 | 41 | 76.9 | Yes |
| **Kr-M-024** | G2a | 38 | 100.0 |  | G1 M342 | 29 | 46.5 | No, but only different sub-clade |
| **Kr-M-025** | J2a1b | 63 | 81.1 |  | J2a1 M67>> S25258 | 54 | 93.7 | Yes |
| **Kr-M-026** | J2a1b | 55 | 62.6 |  | J2a1 PF5191 | 43 | 73.7 | Yes |
| **Kr-M-027** | J1 | 59 | 99.3 |  | J1a2a1a2 P58 | 43 | 92.8 | Yes |
| **Kr-M-028** | J1 | 44 | 97.5 |  | J1a2a2 PF7264> PF7263 | 52 | 100.0 | Yes |
| **Kr-M-029** | G2a | 32 | 100.0 |  | G2a2b2a1b - L497 | 23 | 11.8 | Yes |
| **Kr-M-030** | G2a | 35 | 99.4 |  | G1 M342 | 23 | 11.0 | No |
| **Kr-M-031** | E1b1b | 84 | 100.0 |  | E1b1b | 53 | 100.0 | Yes |
| **Kr-M-032** | R1a | 64 | 100.0 |  | R1a | 44 | 100.0 | Yes |
| **Kr-M-033** | J1 | 44 | 97.5 |  | J1a2a2 PF7264> PF7263 | 52 | 100.0 | Yes |
| **Kr-M-034** | J1 | 57 | 97.4 |  | J1a2a1a2 P58 | 41 | 76.9 | Yes |
| **Kr-M-035** | J1 | 42 | 85.5 |  | J1a2a1a2 P58 | 26 | 82.9 | Yes |
| **Kr-M-036** | G2a | 38 | 59.3 |  | G2a2b2a1a U1 | 26 | 15.6 | Yes |
| **Kr-M-038** | H | 58 | 100.0 |  | H1a M82 | 62 | 100.0 | Yes |
| **Kr-M-039** | J1 | 44 | 97.5 |  | J1a2a2 PF7264> PF7263 | 52 | 100.0 | Yes |
| **Kr-M-040** | L | 53 | 100.0 |  | L1a | 39 | 100.0 | Yes |
| **Kr-M-041** | I2a1 | 30 | 80.5 |  | G2a2b2a1a U1 | 25 | 14.5 | No |
| **Kr-M-042** | R1a | 80 | 100.0 |  | R1a | 63 | 100.0 | Yes |
| **Kr-M-043** | R1b | 39 | 100.0 |  | R1b | 33 | 99.9 | Yes |
| **Kr-M-044** | **E1b1a** | 24 | 79.2 |  | E1b1b | 21 | 43.0 | No, but only different sub-clade |
| **Kr-M-045** | J2a1b | 43 | 82.5 |  | J2a1 Z7700 | 29 | 40.9 | Yes |
| **Kr-M-046** | J1 | 59 | 100.0 |  | J1a3 Z1828 | 40 | 72.6 | Yes |
| **Kr-M-047** | E1b1b | 69 | 100.0 |  | E1b1b | 39 | 99.9 | Yes |
| **Kr-M-048** | E1b1b | 29 | 98.5 |  | E1b1b | 21 | 43.0 | Yes |
| **Kr-M-049** | E1b1b | 45 | 100.0 |  | E1b1b | 28 | 93.9 | Yes |
| **Kr-M-050** | J2a1 x J2a1b/h | 53 | 95.5 |  | J2a1 PF7431 | 30 | 50.1 | Yes |
| **Kr-M-051** | J2a1b | 44 | 82.4 |  | J2a1 Z6065 | 42 | 48.3 | Yes |
| **Kr-M-053** | R1a | 84 | 100.0 |  | R1a | 56 | 100.0 | Yes |
| **Kr-M-054** | J2a1 x J2a1b/h | 39 | 61.3 |  | J2b2 M241 | 27 | 32.3 | No, but only different sub-clade |
| **Kr-M-055** | J2a1b | 63 | 80.9 |  | J2a1 Z387 | 33 | 24.6 | Yes |
| **Kr-M-056** | E1b1b | 56 | 100.0 |  | E1b1b | 36 | 99.8 | Yes |
| **Kr-M-057** | J1 | 45 | 94.3 |  | J1a2a2 PF7264> PF7263 | 68 | 100.0 | Yes |
| **Kr-M-058** | R1a | 64 | 100.0 |  | R1a | 44 | 100.0 | Yes |
| **Kr-M-059** | G2a | 65 | 99.8 |  | G2a2b2a1a U1 | 38 | 41.7 | Yes |
| **Kr-M-061** | J1 | 48 | 99.9 |  | J1a2a1a2 P58 | 27 | 82.3 | Yes |
| **Kr-M-062** | J2a1b | 65 | 96.4 |  | J2a1 Z7671 | 34 | 39.6 | Yes |
| **Kr-M-063** | J2a1b | 63 | 80.9 |  | J2a1 Z387 | 33 | 24.6 | Yes |
| **Kr-M-064** | J2a1b | 69 | 60.2 |  | J2a1 L26>Z500 | 51 | 99.9 | Yes |
| **Kr-M-065** | J2a1b | 73 | 76.6 |  | J2a1 Z6065 | 51 | 60.4 | Yes |
| **Kr-M-066** | J1 | 49 | 98.1 |  | J1a2a2 PF7264> PF7263 | 55 | 100.0 | Yes |
| **Kr-M-067** | J2a1b | 67 | 91.1 |  | J2a1 Z7671 | 38 | 44.4 | Yes |
| **Kr-M-068** | J2a1b | 50 | 86.6 |  | J2a1 PF5191 | 44 | 96.4 | Yes |
| **Kr-M-069** | E1b1b | 44 | 100.0 |  | E1b1b | 27 | 87.2 | Yes |
| **Kr-M-070** | J1 | 49 | 98.1 |  | J1a2a2 PF7264> PF7263 | 31 | 18.3 | Yes |
| **Kr-M-071** | E1b1b | 40 | 97.1 |  | E1b1b | 30 | 97.4 | Yes |
| **Kr-M-072** | R1a | 85 | 100.0 |  | R1a | 57 | 100.0 | Yes |
| **Kr-M-073** | J2a1b | 45 | 51.0 |  | J2a1 Z7700 | 27 | 17.8 | Yes |
| **Kr-M-075** | R1a | 92 | 100.0 |  | R1a | 62 | 100.0 | Yes |
| **Kr-M-076** | E1b1b | 63 | 100.0 |  | E1b1b | 36 | 99.8 | Yes |
| **Kr-M-077** | J2a1 x J2a1b/h | 69 | 77.7 |  | J2a1 M67>> S25258 | 47 | 81.4 | Yes |
| **Kr-M-078** | L | 67 | 100.0 |  | L1a | 43 | 100.0 | Yes |
| **Kr-M-079** | R1a | 71 | 100.0 |  | R1a | 57 | 100.0 | Yes |
| **Kr-M-080** | L | 26 | 52.2 |  | R2 | 51 | 100.0 | No |
| **Kr-M-081** | R1a | 77 | 100.0 |  | R1a | 53 | 100.0 | Yes |
| **Kr-M-082** | R1b | 45 | 100.0 |  | R1b | 39 | 100.0 | Yes |
| **Kr-M-083** | R1a | 68 | 100.0 |  | R1a | 55 | 100.0 | Yes |
| **Kr-M-084** | E1b1b | 44 | 100.0 |  | E1b1b | 26 | 81.3 | Yes |
| **Kr-M-085** | R1a | 92 | 100.0 |  | R1a | 61 | 100.0 | Yes |
| **Kr-M-086** | R1a | 57 | 100.0 |  | R1a | 40 | 100.0 | Yes |
| **Kr-M-087** | E1b1b | 47 | 100.0 |  | E1b1b | 30 | 95.2 | Yes |
| **Kr-M-088** | E1b1a | 31 | 98.8 |  | E1b1b | 23 | 61.8 | No, but only different sub-clade |
| **Kr-M-089** | I2a xI2a1 | 34 | 67.3 |  | I2a1 Isles | 17 | 0.2 | No, but only different sub-clade |
| **Kr-M-090** | I2a xI2a1 | 34 | 67.3 |  | I2a1 Isles | 17 | 0.2 | No, but only different sub-clade |
| **Kr-M-091** | J2a1b | 33 | 59.6 |  | J2a1 Z7671 | 24 | 2.4 | Yes |
| **Kr-M-092** | J1 | 44 | 97.5 |  | J1a2a2 PF7264> PF7263 | 52 | 100.0 | Yes |
| **Kr-M-093** | E1b1b | 46 | 100.0 |  | E1b1b | 30 | 97.3 | Yes |
| **Kr-M-094** | J2a1b | 40 | 89.6 |  | J2a2 PF5008 | 29 | 42.6 | No, but only different sub-clade |
| **Kr-M-095** | J1 | 55 | 99.5 |  | J1a2a1a2 P58 | 40 | 74.6 | Yes |
| **Kr-M-096** | **E1b1b** | 17 | 55.6 |  | E1b1b | 17 | 17.3 | Yes |
| **Kr-M-097** | T | 33 | 96.0 |  | T-Y11151 | 45 | 98.9 | Yes |
| **Kr-M-098** | J2a1b | 81 | 81.5 |  | J2a1 Z467> L210 | 52 | 32.0 | Yes |
| **Kr-M-099** | J2a1b | 40 | 89.6 |  | J2a2 PF5008 | 29 | 42.6 | No, but only different sub-clade |
| **Kr-M-100** | R1a | 37 | 100.0 |  | R1a | 28 | 99.9 | Yes |
| **Kr-M-101** | J1 | 69 | 99.7 |  | J1a2a1a2 P58 | 35 | 32.4 | Yes |
| **Kr-M-102** | E1b1b | 42 | 100.0 |  | E1b1b | 27 | 86.8 | Yes |
| **Kr-M-103** | J1 | 68 | 99.8 |  | J1a2a1a2 P58 | 36 | 88.7 | Yes |
| **Kr-M-104** | G2a | 59 | 93.8 |  | G2a >> PF3359 | 48 | 97.0 | Yes |
| **Kr-M-105** | R1a | 48 | 100.0 |  | R1a | 34 | 100.0 | Yes |
| **Kr-M-106** | J2a1b | 82 | 60.2 |  | J2a1 Z7700 | 56 | 67.0 | Yes |
| **Kr-M-107** | G2a | 67 | 100.0 |  | G2a2b1 M406 | 56 | 99.4 | Yes |
| **Kr-M-108** | G2a | 44 | 99.9 |  | G2a2a PF3147 | 30 | 31.2 | Yes |
| **Kr-M-109** | J2a1b | 81 | 81.5 |  | J2a1 Z467> L210 | 52 | 32.0 | Yes |
| **Kr-M-110** | R1a | 76 | 100.0 |  | R1a | 50 | 100.0 | Yes |
| **Sy-M-001** | R1b | 60 | 100.0 |  | R1b | 46 | 100.0 | Yes |
| **Sy-M-002** | T | 77 | 100.0 |  | T>PF5633 | 51 | 99.3 | Yes |
| **Sy-M-003** | J1 | 43 | 87.5 |  | J1a3 Z1828 | 49 | 99.1 | Yes |
| **Sy-M-004** | I1 | 45 | 68.2 |  | I1 | 32 | 43.1 | Yes |
| **Sy-M-005** | R1b | 49 | 100.0 |  | R1b | 38 | 100.0 | Yes |
| **Sy-M-006** | R1b | 51 | 100.0 |  | R1b | 43 | 100.0 | Yes |
| **Sy-M-007** | R1b | 51 | 100.0 |  | R1b | 38 | 100.0 | Yes |
| **Sy-M-008** | R1b | 51 | 100.0 |  | R1b | 38 | 100.0 | Yes |
| **Sy-M-009** | J1 | 43 | 87.5 |  | J1a3 Z1828 | 49 | 99.1 | Yes |
| **Sy-M-010** | R1b | 49 | 100.0 |  | R1b | 40 | 100.0 | Yes |
| **Sy-M-011** | J2a1 x J2a1b/h | 54 | 59.5 |  | J2a1 M319 | 34 | 29.3 | Yes |
| **Sy-M-012** | J2b | 26 | 100.0 |  | J2b2 M241 | 19 | 9.7 | Yes |
| **Sy-M-013** | R1a | 68 | 100.0 |  | R1a | 44 | 100.0 | Yes |
| **Sy-M-014** | J2a1 x J2a1b/h | 56 | 85.8 |  | J2a1 Z6065 | 37 | 50.4 | Yes |
| **Sy-M-015** | R1a | 73 | 100.0 |  | R1a | 49 | 100.0 | Yes |
| **Sy-M-016** | J1 | 83 | 100.0 |  | J1a2a1a2 P58 | 41 | 90.9 | Yes |
| **Sy-M-017** | R1b | 51 | 100.0 |  | R1b | 43 | 100.0 | Yes |
| **Sy-M-018** | J1 | 77 | 99.9 |  | J1a2a1a2 P58 | 55 | 97.2 | Yes |
| **Sy-M-019** | T | 67 | 100.0 |  | T>PF5633 | 44 | 99.8 | Yes |
| **Sy-M-020** | J2a1 x J2a1b/h | 54 | 59.5 |  | J2a1 M319 | 34 | 29.3 | Yes |
| **Sy-M-021** | J2a1b | 55 | 68.3 |  | J2a1 L26>Z500 | 48 | 100.0 | Yes |
| **Sy-M-023** | R1b | 56 | 100.0 |  | R1b | 36 | 100.0 | Yes |
| **Sy-M-024** | R1a | 49 | 100.0 |  | R1a | 34 | 100.0 | Yes |
| **Sy-M-025** | J2a1b | 74 | 90.7 |  | J2a1 L26>Z500 | 47 | 58.3 | Yes |
| **Sy-M-026** | J2a1b | 79 | 85.3 |  | J2a1 Z7671 | 45 | 54.5 | Yes |
| **Sy-M-028** | T | 68 | 99.9 |  | T>PF5633 | 47 | 99.4 | Yes |
| **Sy-M-029** | T | 72 | 99.9 |  | T>PF5633 | 51 | 99.5 | Yes |
| **Sy-M-030** | R1b | 51 | 100.0 |  | R1b | 38 | 100.0 | Yes |
| **Sy-M-031** | J2a1b | 74 | 90.7 |  | J2a1 L26>Z500 | 47 | 58.3 | Yes |
| **Sy-M-032** | J2a1b | 74 | 90.7 |  | J2a1 L26>Z500 | 47 | 58.3 | Yes |
| **Sy-M-033** | J2a1 x J2a1b/h | 49 | 94.9 |  | J2a1 L26>Z500 | 25 | 28.2 | Yes |
| **Sy-M-034** | J2a1b | 74 | 90.7 |  | J2a1 L26>Z500 | 47 | 58.3 | Yes |
| **Sy-M-035** | R1b | 36 | 100.0 |  | R1b | 33 | 99.9 | Yes |
| **Sy-M-037** | J1 | 77 | 100.0 |  | J1a2a1a2 P58 | 56 | 84.8 | Yes |
| **Sy-M-038** | R1b | 51 | 100.0 |  | R1b | 38 | 100.0 | Yes |
| **Sy-M-039** | J2a1b | 74 | 90.7 |  | J2a1 L26>Z500 | 47 | 58.3 | Yes |
| **Sy-M-040** | J1 | 83 | 99.9 |  | J1a2a1a2 P58 | 65 | 83.6 | Yes |
| **Sy-M-041** | R1a | 49 | 100.0 |  | R1a | 34 | 100.0 | Yes |
| **Sy-M-042** | R1a | 54 | 100.0 |  | R1a | 37 | 100.0 | Yes |
| **Sy-M-043** | J2a1b | 79 | 85.3 |  | J2a1 Z7671 | 45 | 54.5 | Yes |
| **Sy-M-045** | R1b | 57 | 100.0 |  | R1b | 47 | 100.0 | Yes |
| **Sy-M-046** | J2a1 x J2a1b/h | 58 | 51.0 |  | J2a1 Z7671 | 36 | 40.7 | Yes |
| **Sy-M-047** | T | 72 | 99.9 |  | T>PF5633 | 51 | 99.5 | Yes |
| **Sy-M-048** | R1b | 51 | 100.0 |  | R1b | 43 | 100.0 | Yes |
| **Sy-M-049** | J2a1b | 55 | 100.0 |  | J2a1 Z6063 | 28 | 2.9 | Yes |
| **Sy-M-050** | J1 | 80 | 99.5 |  | J1a2a1a2 P58 | 63 | 94.2 | Yes |
| **Sy-M-051** | R1b | 59 | 100.0 |  | R1b | 51 | 100.0 | Yes |
| **Sy-M-052** | T | 68 | 99.9 |  | T>PF5633 | 47 | 99.4 | Yes |
| **Sy-M-053** | R1b | 51 | 100.0 |  | R1b | 38 | 100.0 | Yes |
| **Sy-M-054** | T | 78 | 100.0 |  | T>PF5633 | 34 | 99.0 | Yes |
| **Sy-M-055** | J2a1b | 74 | 90.7 |  | J2a1 L26>Z500 | 47 | 58.3 | Yes |
| **Sy-M-056** | L | 30 | 99.3 |  | L1a | 18 | 22.2 | Yes |
| **Sy-M-057** | J2a1 x J2a1b/h | 49 | 94.9 |  | J2a1 L26>Z500 | 25 | 28.2 | Yes |
| **Sy-M-058** | J2a1b | 74 | 90.7 |  | J2a1 L26>Z500 | 47 | 58.3 | Yes |
| **Sy-M-059** | T | 56 | 99.8 |  | T>PF5633 | 36 | 99.7 | Yes |
| **Sy-M-060** | R1a | 54 | 100.0 |  | R1a | 38 | 100.0 | Yes |
| **Sy-M-061** | J1 | 43 | 87.5 |  | J1a3 Z1828 | 49 | 99.1 | Yes |
| **Sy-M-062** | J2a1 x J2a1b/h | 49 | 94.9 |  | J2a1 L26>Z500 | 25 | 28.2 | Yes |
| **Sy-M-063** | E1b1b | 57 | 99.8 |  | E1b1b | 35 | 85.4 | Yes |
| **Sy-M-064** | R1b | 60 | 100.0 |  | R1b | 46 | 100.0 | Yes |
| **Sy-M-065** | R1b | 60 | 100.0 |  | R1b | 45 | 100.0 | Yes |
| **Sy-M-066** | R1b | 51 | 100.0 |  | R1b | 38 | 100.0 | Yes |
| **Sy-M-067** | J1 | 77 | 100.0 |  | J1a2a1a2 P58 | 56 | 84.8 | Yes |
| **Sy-M-068** | R1b | 51 | 100.0 |  | R1b | 38 | 100.0 | Yes |
| **Sy-M-069** | R1b | 40 | 100.0 |  | R1b | 36 | 100.0 | Yes |
| **Sy-M-070** | T | 91 | 100.0 |  | T>PF5633 | 62 | 99.9 | Yes |
| **Sy-M-071** | R1a | 54 | 100.0 |  | R1a | 39 | 100.0 | Yes |
| **Sy-M-072** | R1b | 51 | 100.0 |  | R1b | 38 | 100.0 | Yes |
| **Sy-M-073** | E1b1b | 79 | 100.0 |  | E1b1b | 49 | 100.0 | Yes |
| **Sy-M-074** | T | 78 | 100.0 |  | T>PF5633 | 34 | 99.0 | Yes |
| **Sy-M-075** | R1b | 60 | 100.0 |  | R1b | 46 | 100.0 | Yes |
| **Sy-M-076** | R1a | 54 | 100.0 |  | R1a | 38 | 100.0 | Yes |
| **Sy-M-077** | R1b | 60 | 100.0 |  | R1b | 46 | 100.0 | Yes |
| **Sy-M-078** | T | 77 | 100.0 |  | T>PF5633 | 51 | 99.3 | Yes |
| **Sy-M-079** | T | 91 | 100.0 |  | T>PF5633 | 62 | 99.9 | Yes |
| **Sy-M-080** | I1 | 45 | 68.2 |  | I1 | 32 | 43.1 | Yes |
| **Sy-M-081** | T | 91 | 100.0 |  | T>PF5633 | 62 | 99.9 | Yes |
| **Sy-M-082** | R1b | 65 | 100.0 |  | R1b | 50 | 100.0 | Yes |
| **Sy-M-084** | J2a1b | 83 | 86.7 |  | J2a1 Z467> L210 | 49 | 38.4 | Yes |
| **Sy-M-085** | J1 | 43 | 87.5 |  | J1a3 Z1828 | 49 | 99.1 | Yes |
| **Sy-M-086** | T | 91 | 100.0 |  | T>PF5633 | 62 | 99.9 | Yes |
| **Sy-M-087** | J2a1b | 76 | 97.5 |  | J2a1 L26>Z500 | 46 | 81.8 | Yes |
| **Sy-M-088** | R1b | 51 | 100.0 |  | R1b | 38 | 100.0 | Yes |
| **Sy-M-089** | R1a | 43 | 100.0 |  | R1a | 32 | 99.9 | Yes |
| **Sy-M-091** | T | 68 | 99.9 |  | T>PF5633 | 47 | 99.4 | Yes |
| **Sy-M-092** | R1b | 50 | 100.0 |  | R1b | 40 | 100.0 | Yes |
| **Tm-M-001** | G2a | 65 | 100.0 |  | G2a2b2a1a U1 | 48 | 49.5 | Yes |
| **Tm-M-002** | J2a1 x J2a1b/h | 46 | 91.4 |  | J2a1 Z7700 | 35 | 78.0 | Yes |
| **Tm-M-003** | G2a | 47 | 99.9 |  | G2a2b2a1b - L497 | 31 | 24.1 | Yes |
| **Tm-M-004** | R1a | 52 | 100.0 |  | R1a | 36 | 100.0 | Yes |
| **Tm-M-005** | R1a | 67 | 100.0 |  | R1a | 54 | 100.0 | Yes |
| **Tm-M-006** | R1b | 47 | 100.0 |  | R1b | 38 | 100.0 | Yes |
| **Tm-M-007** | R1a | 64 | 100.0 |  | R1a | 44 | 100.0 | Yes |
| **Tm-M-009** | E1b1b | 86 | 100.0 |  | E1b1b | 53 | 100.0 | Yes |
| **Tm-M-010** | E1b1b | 77 | 100.0 |  | E1b1b | 48 | 100.0 | Yes |
| **Tm-M-011** | J1 | 77 | 100.0 |  | J1a2a1a2 P58 | 71 | 100.0 | Yes |
| **Tm-M-012** | R1a | 84 | 100.0 |  | R1a | 58 | 100.0 | Yes |
| **Tm-M-013** | **J2b** | 45 | 43.6 |  | J2b2 M241 | 30 | 44.8 | Yes |
| **Tm-M-014** | E1b1b | 64 | 100.0 |  | E1b1b | 31 | 97.4 | Yes |
| **Tm-M-015** | J2a1b | 58 | 57.9 |  | J2b2-Z2456 | 45 | 91.9 | No, but only different sub-clade |
| **Tm-M-017** | J2a1b | 31 | 81.8 |  | G2a2a PF3147 | 21 | 6.3 | No |
| **Tm-M-018** | I2a1 | 70 | 100.0 |  | I2a1a Sardinian M26 | 50 | 100.0 | Yes |
| **Tm-M-019** | J2a1b | 55 | 82.2 |  | J2a1 Z6065 | 42 | 80.8 | Yes |
| **Tm-M-020** | J1 | 57 | 99.9 |  | J1a2a2 PF7264> PF7263 | 48 | 99.9 | Yes |
| **Tm-M-021** | **J1** | 50 | 39.0 |  | J2a1 Z7700 | 42 | 59.6 | No |
| **Tm-M-023** | J1 | 40 | 99.5 |  | J1a2a2 PF7264> PF7263 | 58 | 100.0 | Yes |
| **Tm-M-024** | J2a1b | 61 | 55.6 |  | J2a1 PF5191 | 35 | 26.0 | Yes |
| **Tm-M-025** | R1b | 47 | 100.0 |  | R1b | 40 | 100.0 | Yes |
| **Tm-M-026** | L | 53 | 100.0 |  | L1a | 39 | 100.0 | Yes |
| **Tm-M-027** | R1b | 51 | 100.0 |  | R1b | 45 | 100.0 | Yes |
| **Tm-M-028** | E1b1b | 60 | 99.9 |  | E1b1b | 31 | 97.4 | Yes |
| **Tm-M-029** | J1 | 39 | 100.0 |  | J1a3 Z1828 | 32 | 41.6 | Yes |
| **Tm-M-030** | J1 | 70 | 98.6 |  | J1a2a1a2 P58 | 63 | 98.1 | Yes |
| **Tm-M-031** | J1 | 48 | 98.5 |  | J2a1 L26>Z500 | 22 | 8.0 | No |
| **Tm-M-033** | R1b | 47 | 100.0 |  | R1b | 40 | 100.0 | Yes |
| **Tm-M-034** | E1b1b | 59 | 100.0 |  | E1b1b | 43 | 100.0 | Yes |
| **Tm-M-035** | E1b1b | 54 | 95.5 |  | E1b1b | 37 | 100.0 | Yes |
| **Tm-M-036** | J2a1b | 67 | 91.1 |  | J2a1 Z7671 | 38 | 44.4 | Yes |
| **Tm-M-037** | **E1b1a** | 23 | 91.0 |  | E1b1b | 21 | 43.1 | No, but only different sub-clade |
| **Tm-M-038** | E1b1b | 58 | 99.8 |  | E1b1b | 32 | 98.4 | Yes |
| **Tm-M-039** | G2a | 33 | 81.1 |  | G2a2b1 M406 | 26 | 22.8 | Yes |
| **Tm-M-040** | J2a1b | 56 | 79.5 |  | J2a1 Z6065 | 41 | 58.8 | Yes |
| **Tm-M-041** | J2a1b | 67 | 91.1 |  | J2a1 Z7671 | 38 | 44.4 | Yes |
| **Tm-M-042** | T | 78 | 100.0 |  | T>PF5633 | 51 | 99.5 | Yes |
| **Tm-M-043** | R1a | 64 | 100.0 |  | R1a | 44 | 100.0 | Yes |
| **Tm-M-044** | R1b | 33 | 99.9 |  | R1b | 26 | 31.9 | Yes |
| **Tm-M-045** | J1 | 45 | 94.3 |  | J1a2a2 PF7264> PF7263 | 68 | 100.0 | Yes |
| **Tm-M-046** | E1b1b | 58 | 99.8 |  | E1b1b | 32 | 98.4 | Yes |
| **Tm-M-047** | R1a | 57 | 100.0 |  | R1a | 44 | 100.0 | Yes |
| **Tm-M-048** | T | 30 | 98.9 |  | T>PF5633 | 16 | 5.7 | Yes |
| **Tm-M-049** | E1b1b | 60 | 100.0 |  | E1b1b | 36 | 99.8 | Yes |
| **Tm-M-050** | G2a | 65 | 99.8 |  | G2a2b2a1a U1 | 38 | 41.7 | Yes |
| **Tm-M-051** | J2a1h | 33 | 87.5 |  | J2a1 PF7431 | 47 | 89.0 | Yes |
| **Tm-M-052** | J2a1b | 55 | 82.2 |  | J2a1 Z6065 | 42 | 80.8 | Yes |
| **Tm-M-053** | L | 34 | 100.0 |  | R2 | 41 | 100.0 | No |
| **Tm-M-054** | J1 | 52 | 99.9 |  | J1a2a1a2 P58 | 34 | 87.8 | Yes |
| **Tm-M-055** | R1a | 38 | 100.0 |  | R1a | 32 | 100.0 | Yes |
| **Tm-M-056** | J2a1h | 80 | 100.0 |  | J2a1 Z387 | 61 | 99.9 | Yes |
| **Tm-M-057** | T | 45 | 99.9 |  | T-Y11151 | 49 | 77.5 | Yes |
| **Tm-M-058** | G2a | 41 | 99.6 |  | G2a2b1 M406 | 32 | 38.2 | Yes |
| **Tm-M-059** | G2a | 41 | 99.6 |  | G2a2b1 M406 | 32 | 38.2 | Yes |
| **Tm-M-060** | R1b | 49 | 100.0 |  | R1b | 39 | 100.0 | Yes |
| **Tm-M-061** | L | 34 | 100.0 |  | R2 | 41 | 100.0 | No |
| **Tm-M-062** | Q | 58 | 98.4 |  | Q L275>> L245 | 27 | 98.3 | Yes |
| **Tm-M-063** | E1b1b | 42 | 100.0 |  | E1b1b | 30 | 97.4 | Yes |
| **Tm-M-064** | R1b | 47 | 100.0 |  | R1b | 40 | 100.0 | Yes |
| **Tm-M-065** | J2a1b | 71 | 85.2 |  | J2a1 Z7700 | 37 | 16.7 | Yes |
| **Tm-M-066** | Q | 64 | 99.9 |  | Q L275>> L245 | 52 | 100.0 | Yes |
| **Tm-M-067** | J1 | 39 | 93.5 |  | J1a2a2 PF7264> PF7263 | 54 | 100.0 | Yes |
| **Tm-M-068** | G2a | 36 | 61.2 |  | G2a1-L293 | 16 | 2.7 | Yes |
| **Tm-M-069** | E1b1b | 74 | 100.0 |  | E1b1b | 47 | 100.0 | Yes |
| **Tm-M-070** | E1b1b | 44 | 100.0 |  | E1b1b | 30 | 97.4 | Yes |
| **Tm-M-071** | T | 81 | 100.0 |  | T>PF5633 | 57 | 99.9 | Yes |
| **Tm-M-072** | R1a | 42 | 100.0 |  | R1a | 32 | 100.0 | Yes |
| **Tm-M-073** | T | 63 | 99.8 |  | T>PF5633 | 35 | 99.8 | Yes |
| **Tm-M-074** | J2a1h | 33 | 87.5 |  | J2a1 PF7431 | 47 | 89.0 | Yes |
| **Tm-M-075** | G2a | 72 | 100.0 |  | G2a2b2a1b - L497 | 47 | 92.4 | Yes |
| **Tm-M-076** | J1 | 63 | 100.0 |  | J1a2a1a2 P58 | 70 | 100.0 | Yes |
| **Tm-M-077** | J2a1b | 67 | 91.1 |  | J2a1 Z7671 | 38 | 44.4 | Yes |
| **Tm-M-078** | E1b1b | 58 | 99.9 |  | E1b1b | 30 | 97.4 | Yes |
| **Tm-M-079** | R1a | 72 | 100.0 |  | R1a | 49 | 100.0 | Yes |
| **Tm-M-080** | J1 | 54 | 96.4 |  | J1a2a2 PF7264> PF7263 | 69 | 100.0 | Yes |
| **Tm-M-081** | R1b | 57 | 100.0 |  | R1b | 44 | 100.0 | Yes |
| **Tm-M-082** | J2a1 x J2a1b/h | 72 | 75.6 |  | J2a1 PF5191 | 41 | 35.5 | Yes |
| **Tm-M-084** | I2a xI2a1 | 75 | 99.9 |  | I2a1b3 Carpathian-Dinaric & Disles L621 | 55 | 100.0 | Yes |
| **Tm-M-085** | E1b1b | 48 | 71.5 |  | J1a2a1a2 P58 | 44 | 99.4 | No |
| **Tm-M-086** | **I2a1** | 22 | 98.7 |  | I2a1a Sardinian M26 | 17 | 11.1 | Yes |
| **Tm-M-087** | E1b1a | 80 | 100.0 |  | E1b1a V38 | 51 | 100.0 | Yes |
| **Tm-M-088** | E1b1b | 44 | 93.0 |  | J1a2a1a2 P58 | 27 | 69.9 | No |
| **Tm-M-089** | J2a1h | 51 | 99.9 |  | J2a1 Z387 | 33 | 52.6 | Yes |
| **Tm-M-090** | J2a1b | 92 | 78.6 |  | J2a1 Z7671 | 57 | 88.9 | Yes |
| **Tm-M-091** | L | 84 | 100.0 |  | L1b | 53 | 100.0 | Yes |
| **Tm-M-093** | J2a1 x J2a1b/h | 43 | 84.7 |  | J2a1 Z6065 | 37 | 53.4 | Yes |
| **Tm-M-095** | J2a1 x J2a1b/h | 60 | 67.4 |  | J2a1 Z7700 | 52 | 91.8 | Yes |
| **Tm-M-096** | G2a | 41 | 99.6 |  | G2a2b1 M406 | 32 | 38.2 | Yes |
| **Tm-M-097** | **I2a1** | 22 | 98.7 |  | I2a1a Sardinian M26 | 17 | 11.1 | Yes |
| **Tm-M-098** | E1b1b | 47 | 100.0 |  | E1b1b | 29 | 96.1 | Yes |
| **Tm-M-099** | L | 84 | 100.0 |  | L1b | 53 | 100.0 | Yes |
| **Tm-M-100** | G2a | 47 | 99.9 |  | G2a2b2a1b - L497 | 31 | 24.1 | Yes |
| **Tm-M-101** | R1a | 62 | 100.0 |  | R1a | 48 | 100.0 | Yes |
| **Tm-M-103** | T | 45 | 99.9 |  | T-Y11151 | 49 | 77.5 | Yes |
| **Tm-M-104** | J1 | 39 | 100.0 |  | J1a3 Z1828 | 32 | 41.6 | Yes |
| **Tm-M-105** | R1a | 37 | 100.0 |  | R1a | 30 | 99.9 | Yes |
| **Tm-M-106** | E1b1b | 68 | 100.0 |  | E1b1b | 47 | 100.0 | Yes |
| **Tm-M-107** | R1a | 39 | 100.0 |  | R1a | 31 | 100.0 | Yes |
| **Tm-M-108** | I1 | 34 | 90.3 |  | I1 | 28 | 46.5 | Yes |
| **Tm-M-109** | H | 55 | 100.0 |  | H1a M82 | 41 | 93.0 | Yes |
| **Tm-M-110** | J2a1b | 67 | 91.1 |  | J2a1 Z7671 | 38 | 44.4 | Yes |
| **Yz-M-001** | J1 | 59 | 97.8 |  | J1a3 Z1828 | 66 | 99.8 | Yes |
| **Yz-M-002** | J2a1 x J2a1b/h | 40 | 57.4 |  | J2a1 L26>Z500 | 27 | 53.5 | Yes |
| **Yz-M-003** | R1b | 55 | 100.0 |  | R1b | 39 | 100.0 | Yes |
| **Yz-M-004** | J1 | 48 | 64.0 |  | E1b1b | 22 | 38.9 | No |
| **Yz-M-006** | J1 | 48 | 64.0 |  | E1b1b | 22 | 38.9 | No |
| **Yz-M-007** | J2a1 x J2a1b/h | 35 | 91.7 |  | E1b1b | 22 | 13.9 | No |
| **Yz-M-008** | J2a1 x J2a1b/h | 35 | 91.7 |  | E1b1b | 22 | 13.9 | No |
| **Yz-M-009** | L | 66 | 100.0 |  | L1b | 31 | 100.0 | Yes |
| **Yz-M-010** | L | 66 | 100.0 |  | L1b | 31 | 100.0 | Yes |
| **Yz-M-011** | R1b | 31 | 99.5 |  | R1a YP4141 | 46 | 100.0 | No |
| **Yz-M-012** | R1b | 31 | 99.5 |  | R1a YP4141 | 46 | 100.0 | No |
| **Yz-M-013** | R1b | 31 | 99.5 |  | R1a YP4141 | 24 | 5.0 | No |
| **Yz-M-014** | L | 66 | 100.0 |  | L1b | 28 | 100.0 | Yes |
| **Yz-M-015** | L | 66 | 100.0 |  | L1b | 28 | 100.0 | Yes |
| **Yz-M-016** | L | 66 | 100.0 |  | L1b | 31 | 100.0 | Yes |
| **Yz-M-017** | R1b | 39 | 100.0 |  | R1b | 27 | 99.8 | Yes |
| **Yz-M-018** | R1b | 56 | 100.0 |  | R1b | 40 | 100.0 | Yes |
| **Yz-M-019** | R1b | 56 | 100.0 |  | R1b | 40 | 100.0 | Yes |
| **Yz-M-020** | R1b | 56 | 100.0 |  | R1b | 40 | 100.0 | Yes |
| **Yz-M-023** | G2a | 49 | 100.0 |  | G2a2b2a1c - Z724 | 32 | 53.0 | Yes |
| **Yz-M-024** | L | 66 | 100.0 |  | L1b | 30 | 100.0 | Yes |
| **Yz-M-025** | E1b1b | 74 | 100.0 |  | E1b1b | 51 | 100.0 | Yes |
| **Yz-M-026** | R1b | 56 | 100.0 |  | R1b | 40 | 100.0 | Yes |
| **Yz-M-027** | J2a1b | 81 | 69.3 |  | J2a1 Z7671 | 47 | 65.5 | Yes |
| **Yz-M-028** | I2a xI2a1 | 56 | 77.8 |  | E1b1b | 25 | 51.8 | No |
| **Yz-M-029** | R1b | 55 | 100.0 |  | R1b | 39 | 100.0 | Yes |
| **Yz-M-030** | J2a1b | 81 | 69.3 |  | J2a1 Z7671 | 47 | 65.5 | Yes |
| **Yz-M-031** | T | 52 | 98.3 |  | T>PF5633 | 31 | 97.3 | Yes |
| **Yz-M-032** | E1b1b | 74 | 100.0 |  | E1b1b | 51 | 100.0 | Yes |
| **Yz-M-033** | R1b | 42 | 100.0 |  | R1b | 31 | 100.0 | Yes |
| **Yz-M-034** | T | 52 | 98.3 |  | T>PF5633 | 31 | 97.3 | Yes |
| **Yz-M-035** | L | 68 | 100.0 |  | L1b | 24 | 100.0 | Yes |
| **Yz-M-036** | J2a1b | 77 | 91.2 |  | J2a1 M67>> S25258 | 50 | 37.8 | Yes |
| **Yz-M-037** | G2a | 41 | 69.6 |  | E1a | 37 | 77.6 | No |
| **Yz-M-038** | R1b | 37 | 99.9 |  | R1a YP4141 | 30 | 53.2 | No, but only different sub-clade |
| **Yz-M-039** | R1b | 42 | 100.0 |  | R1b | 32 | 100.0 | Yes |
| **Yz-M-040** | G2a | 37 | 85.9 |  | E1a | 33 | 41.3 | No |
| **Yz-M-041** | L | 68 | 100.0 |  | L1b | 24 | 100.0 | Yes |
| **Yz-M-042** | T | 52 | 98.3 |  | T>PF5633 | 31 | 97.3 | Yes |
| **Yz-M-043** | E1b1b | 74 | 100.0 |  | E1b1b | 51 | 100.0 | Yes |
| **Yz-M-044** | J1 | 53 | 99.8 |  | J1a3 Z1828 | 41 | 46.6 | Yes |
| **Yz-M-045** | R1b | 44 | 100.0 |  | R1b | 33 | 99.9 | Yes |
| **Yz-M-046** | E1b1a | 67 | 100.0 |  | E1b1a V38 | 51 | 100.0 | Yes |
| **Yz-M-047** | E1b1b | 74 | 100.0 |  | E1b1b | 51 | 100.0 | Yes |
| **Yz-M-048** | E1b1a | 67 | 100.0 |  | E1b1a V38 | 51 | 100.0 | Yes |
| **Yz-M-049** | J1 | 59 | 97.8 |  | J1a3 Z1828 | 66 | 99.8 | Yes |
| **Yz-M-050** | E1b1a | 67 | 100.0 |  | E1b1a V38 | 51 | 100.0 | Yes |
| **Yz-M-051** | R1b | 37 | 99.9 |  | R1a YP4141 | 55 | 100.0 | No, but only different sub-clade |
| **Yz-M-052** | J2a1 x J2a1b/h | 35 | 91.7 |  | E1b1b | 22 | 13.9 | No |
| **Yz-M-053** | J2a1 x J2a1b/h | 35 | 91.7 |  | E1b1b | 22 | 13.9 | No |
| **Yz-M-054** | T | 53 | 99.6 |  | T>PF5633 | 28 | 94.0 | Yes |
| **Yz-M-055** | T | 53 | 99.6 |  | T>PF5633 | 28 | 94.0 | Yes |
| **Yz-M-056** | G2a | 48 | 99.1 |  | E1a | 41 | 95.4 | No |
| **Yz-M-057** | G2a | 48 | 99.1 |  | E1a | 41 | 95.4 | No |
| **Yz-M-058** | G2a | 51 | 98.9 |  | E1a | 39 | 83.1 | No |
| **Yz-M-059** | J2a1b | 81 | 69.3 |  | J2a1 Z7671 | 47 | 65.5 | Yes |
| **Yz-M-060** | J2a1 x J2a1b/h | 35 | 91.7 |  | E1b1b | 22 | 13.9 | No |
| **Yz-M-061** | L | 64 | 100.0 |  | L1b | 25 | 100.0 | Yes |
| **Yz-M-062** | R1b | 56 | 100.0 |  | R1b | 22 | 98.0 | Yes |
| **Yz-M-063** | **J2a1b** | 65 | 48.1 |  | J2a1 L26>Z500 | 25 | 5.6 | Yes |
| **Yz-M-064** | J1 | 50 | 100.0 |  | J1a2a1a2 P58 | 30 | 93.3 | Yes |
| **Yz-M-065** | **J2a1b** | 65 | 48.1 |  | J2a1 Z6063 | 46 | 43.8 | Yes |
| **Yz-M-066** | R1a | 70 | 100.0 |  | R1a | 51 | 100.0 | Yes |
| **Yz-M-067** | T | 52 | 98.3 |  | T>PF5633 | 31 | 97.3 | Yes |
| **Yz-M-068** | E1b1b | 74 | 100.0 |  | E1b1b | 51 | 100.0 | Yes |
| **Yz-M-069** | J2a1 x J2a1b/h | 53 | 100.0 |  | J2a1 PF5191 | 34 | 62.7 | Yes |
| **Yz-M-070** | R1a | 70 | 100.0 |  | R1a | 51 | 100.0 | Yes |
| **Yz-M-071** | R1a | 70 | 100.0 |  | R1a | 51 | 100.0 | Yes |
| **Yz-M-072** | **J2a1b** | 65 | 48.1 |  | J2a1 Z6063 | 46 | 43.8 | Yes |
| **Yz-M-073** | G2a | 40 | 100.0 |  | G2a1-L293 | 39 | 99.1 | Yes |
| **Yz-M-074** | G2a | 40 | 100.0 |  | G2a1-L293 | 39 | 99.1 | Yes |
| **Yz-M-075** | G2a | 40 | 100.0 |  | G2a1-L293 | 39 | 99.1 | Yes |
| **Yz-M-076** | R1a | 80 | 100.0 |  | R1a | 63 | 100.0 | Yes |
| **Yz-M-077** | J1 | 44 | 81.3 |  | J1a2a1a2 P58 | 32 | 40.4 | Yes |
| **Yz-M-078** | J1 | 44 | 81.3 |  | J1a2a1a2 P58 | 32 | 40.4 | Yes |
| **Yz-M-079** | R1a | 80 | 100.0 |  | R1a | 63 | 100.0 | Yes |
| **Yz-M-080** | E1b1b | 74 | 100.0 |  | E1b1b | 51 | 100.0 | Yes |
| **Yz-M-081** | R1b | 50 | 100.0 |  | R1b | 35 | 100.0 | Yes |
| **Yz-M-082** | R1a | 80 | 100.0 |  | R1a | 63 | 100.0 | Yes |
| **Yz-M-083** | J2a1b | 77 | 91.2 |  | J2a1 M67>> S25258 | 50 | 37.8 | Yes |
| **Yz-M-084** | J2a1b | 77 | 91.2 |  | J2a1 M67>> S25258 | 50 | 37.8 | Yes |
| **Yz-M-085** | R1b | 50 | 100.0 |  | R1b | 35 | 100.0 | Yes |
| **Yz-M-086** | R1a | 80 | 100.0 |  | R1a | 34 | 100.0 | Yes |
| **Yz-M-087** | R1a | 80 | 100.0 |  | R1a | 63 | 100.0 | Yes |
| **Yz-M-088** | R1b | 50 | 100.0 |  | R1b | 35 | 100.0 | Yes |
| **Yz-M-089** | J2a1b | 77 | 91.2 |  | J2a1 M67>> S25258 | 50 | 37.8 | Yes |
| **Yz-M-090** | E1b1b | 74 | 100.0 |  | E1b1b | 51 | 100.0 | Yes |
| **Yz-M-091** | R1b | 55 | 100.0 |  | R1b | 40 | 100.0 | Yes |
| **Yz-M-092** | J2a1 x J2a1b/h | 40 | 67.5 |  | J2a1 M319 | 44 | 86.0 | Yes |
| **Yz-M-093** | E1b1b | 74 | 100.0 |  | E1b1b | 51 | 100.0 | Yes |
| **Yz-M-094** | R1a | 34 | 99.5 |  | R1a | 14 | 0.8 | Yes |
| **Yz-M-095** | L | 63 | 100.0 |  | O2a2 F525 | 21 | 8.6 | No |
| **Yz-M-096** | **R1a** | 21 | 68.0 |  | R1b | 22 | 96.8 | No, but only different sub-clade |
| **Yz-M-097** | J2a1b | 59 | 94.4 |  | J2a1 Z387 | 37 | 28.8 | Yes |
| **Yz-M-098** | J2a1b | 63 | 74.3 |  | J2a1 Z6065 | 35 | 23.5 | Yes |
| **Yz-M-099** | J2a1 x J2a1b/h | 26 | 99.2 |  | J2a1 PF5191 | 20 | 7.5 | Yes |
| **Yz-M-100** | G2a | 56 | 100.0 |  | G2a2b1 M406 | 38 | 53.5 | Yes |
| **Yz-M-101** | J2a1 x J2a1b/h | 53 | 100.0 |  | J2a1 PF5191 | 34 | 62.7 | Yes |
| **Yz-M-102** | G2a | 54 | 100.0 |  | G2a2b1 M406 | 38 | 68.0 | Yes |
| **Yz-M-103** | **R1a** | 21 | 68.0 |  | R1b | 22 | 96.8 | No, but only different sub-clade |
| **Yz-M-104** | T | 52 | 98.3 |  | T>PF5633 | 31 | 97.3 | Yes |
| **Yz-M-105** | R1b | 47 | 100.0 |  | R1b | 35 | 100.0 | Yes |
| **Yz-M-106** | L | 44 | 99.8 |  | L1b | 18 | 23.9 | Yes |
| **Yz-M-107** | J1 | 59 | 97.8 |  | J1a3 Z1828 | 66 | 99.8 | Yes |
| **Yz-M-108** | J2a1 x J2a1b/h | 40 | 67.5 |  | J2a1 M319 | 44 | 86.0 | Yes |
| **Yz-M-109** | L | 68 | 100.0 |  | L1b | 24 | 100.0 | Yes |

Abbreviations: M, male; Ar, Arab; Kr, Kurdish; Sy, Syriac; Tm, Turkmen; Yz, Yazidi

Whit Athey Haplogroup assignments in bold denote those below the set algorithmic thresholds (*‘minimum score’/‘fitness score’ of 25 and ‘minimum Bayesian probability’ of 50%*).
