# Supplementary material for: A glimpse at the intricate mosaic of ethnicities from Mesopotamia: Paternal lineages of the Northern Iraqi Arabs, Kurds, Syriacs, Turkmens and Yazidis
Source: PLoS One. 2017 Nov 3;12(11):e0187408. doi: 10.1371/journal.pone.0187408 (PMC5669434; doi:10.1371/journal.pone.0187408)
Supplement: S1 File — Table A: Allele frequencies of the 17 Y-STR loci for the combined Northern Iraqi population (n = 500). Table B: Allele frequencies of the 17 Y-STR loci for the Northern Iraq Arab population (n = 102). Table C: Allele frequencies of the 17 Y-STR loci for the Northern Iraq Kurdish population (n = 104). Table D: Allele frequencies of the 17 Y-STR loci for the Northern Iraq Syriac population (n = 86). Table E Allele frequencies of the 17 Y-STR loci for the Northern Iraq Turkmen population (n = 102). Table F: Allele frequencies of the 17 Y-STR loci for the Northern Iraq Yazidi population (n = 106). (DOCX) [file pone.0187408.s004.docx]

**S1 File Table A.** Allele frequencies of the 17 Y-STR loci for the combined Northern Iraqi population (*n*=500).

| Allele | DYS19 | DYS389I | DYS389II | DYS390 | DYS391 | DYS392 | DYS393 | DYS437 | DYS438 | DYS439 | DYS448 | DYS456 | DYS458 | DYS635 | Y_GATA_H4 | Genotypes | DYS385a/b |
| --- | --- | --- | --- | --- | --- | --- | --- | --- | --- | --- | --- | --- | --- | --- | --- | --- | --- |
| 8 |  |  |  |  |  |  |  |  | 0.0020 |  |  |  |  |  | 0.0020 | 9,11 | 0.0100 |
| 8,11 |  |  |  |  |  |  |  |  |  | 0.0020 |  |  |  |  |  | 9,17 | 0.0020 |
| 9 |  |  |  |  | 0.0440 |  |  |  | 0.2760 |  |  |  |  |  | 0.0020 | 10,14 | 0.0020 |
| 10 |  |  |  |  | 0.6580 | 0.0240 | 0.0020 |  | 0.4260 | 0.1220 |  |  |  |  | 0.0920 | 11,11 | 0.0040 |
| 10,13 |  | 0.0020 |  |  |  |  |  |  |  |  |  |  |  |  |  | 11,12 | 0.0060 |
| 11 |  | 0.0040 |  |  | 0.2660 | 0.6720 | 0.0200 |  | 0.1760 | 0.4040 |  |  |  |  | 0.4360 | 11,13 | 0.0440 |
| 11,12 |  |  |  |  |  |  |  |  |  | 0.0020 |  |  |  |  |  | 11,14 | 0.1160 |
| 11,13 |  | 0.0020 |  |  |  |  |  |  |  |  |  |  |  |  |  | 11,15 | 0.0480 |
| 12 | 0.0020 | 0.1900 |  |  | 0.0300 | 0.0340 | 0.5380 |  | 0.1160 | 0.3760 |  | 0.0060 | 0.0060 |  | 0.3660 | 11,16 | 0.0060 |
| 13 | 0.1240 | 0.5600 |  |  | 0.0020 | 0.1520 | 0.3420 |  | 0.0040 | 0.0740 |  | 0.0220 |  |  | 0.0960 | 11,18 | 0.0020 |
| 14 | 0.5420 | 0.2320 |  |  |  | 0.0960 | 0.0900 | 0.5940 |  | 0.0200 |  | 0.1180 | 0.0300 |  | 0.0060 | 11,19 | 0.0080 |
| 14,16 | 0.0020 |  |  |  |  |  |  |  |  |  |  |  |  |  |  | 12,12 | 0.0180 |
| 15 | 0.2200 | 0.0100 |  |  |  | 0.0180 | 0.0080 | 0.3040 |  |  |  | 0.5180 | 0.2220 |  |  | 12,13 | 0.0040 |
| 16 | 0.0860 |  |  |  |  |  |  | 0.0900 |  |  |  | 0.2800 | 0.2180 |  |  | 12,14 | 0.0180 |
| 16.4 |  |  |  |  |  |  |  |  |  |  | 0.0040 |  |  |  |  | 12,15 | 0.0180 |
| 17 | 0.0240 |  |  |  |  |  |  | 0.0120 |  |  | 0.0060 | 0.0440 | 0.2360 |  |  | 12,16 | 0.0140 |
| 17.2 |  |  |  |  |  |  |  |  |  |  |  |  | 0.0200 |  |  | 12,17 | 0.0100 |
| 18 |  |  |  |  |  |  |  |  |  |  | 0.0160 | 0.0080 | 0.0860 |  |  | 12,18 | 0.0360 |
| 18.2 |  |  |  |  |  |  |  |  |  |  |  |  | 0.0980 |  |  | 12,19 | 0.0060 |
| 19 |  |  |  |  |  |  |  |  |  |  | 0.3400 | 0.0020 | 0.0220 |  |  | 12,20 | 0.0120 |
| 19.2 |  |  |  |  |  |  |  |  |  |  |  |  | 0.0380 |  |  | 12,22 | 0.0040 |
| 19,20 |  |  |  |  |  |  |  |  |  |  | 0.0100 |  |  |  |  | 13,13 | 0.0020 |
| 20 |  |  |  |  |  |  |  |  |  |  | 0.4560 |  | 0.0040 | 0.1060 |  | 13,14 | 0.0040 |
| 20.2 |  |  |  |  |  |  |  |  |  |  |  |  | 0.0140 |  |  | 13,15 | 0.0200 |
| 21 |  |  |  | 0.0260 |  |  |  |  |  |  | 0.1300 |  | 0.0040 | 0.3380 |  | 13,16 | 0.0640 |
| 21,22 |  |  |  |  |  |  |  |  |  |  | 0.0060 |  |  |  |  | 13,17 | 0.0380 |
| 21.2 |  |  |  |  |  |  |  |  |  |  |  |  | 0.0020 |  |  | 13,18 | 0.0380 |
| 22 |  |  |  | 0.1500 |  |  |  |  |  |  | 0.0160 |  |  | 0.1600 |  | 13,19 | 0.0600 |
| 23 |  |  |  | 0.4380 |  |  |  |  |  |  | 0.0040 |  |  | 0.2720 |  | 13,20 | 0.0080 |
| 24 |  |  |  | 0.2420 |  |  |  |  |  |  | 0.0080 |  |  | 0.1000 |  | 13,22 | 0.0020 |
| 25 |  |  |  | 0.1360 |  |  |  |  |  |  |  |  |  | 0.0220 |  | 14,14 | 0.0080 |
| 25,29 |  |  | 0.0020 |  |  |  |  |  |  |  |  |  |  |  |  | 14,15 | 0.0260 |
| 26 |  |  | 0.0060 | 0.0080 |  |  |  |  |  |  |  |  |  | 0.0020 |  | 14,16 | 0.0620 |
| 27 |  |  | 0.0080 |  |  |  |  |  |  |  |  |  |  |  |  | 14,17 | 0.0100 |
| 28 |  |  | 0.1140 |  |  |  |  |  |  |  |  |  |  |  |  | 14,18 | 0.0180 |
| 29 |  |  | 0.3180 |  |  |  |  |  |  |  |  |  |  |  |  | 14,19 | 0.0140 |
| 30 |  |  | 0.3780 |  |  |  |  |  |  |  |  |  |  |  |  | 14,20 | 0.0120 |
| 30.3 |  |  | 0.0060 |  |  |  |  |  |  |  |  |  |  |  |  | 14,21 | 0.0020 |
| 31 |  |  | 0.1020 |  |  |  |  |  |  |  |  |  |  |  |  | 15,15 | 0.0100 |
| 32 |  |  | 0.0640 |  |  |  |  |  |  |  |  |  |  |  |  | 15,16 | 0.0320 |
| 33 |  |  | 0.0020 |  |  |  |  |  |  |  |  |  |  |  |  | 15,17 | 0.0260 |
| Null |  |  |  |  |  | 0.0040 |  |  |  |  | 0.0040 | 0.0020 |  |  |  | 15,18 | 0.0020 |
|  |  |  |  |  |  |  |  |  |  |  |  |  |  |  |  | 15.2, 17 | 0.0040 |
|  |  |  |  |  |  |  |  |  |  |  |  |  |  |  |  | 15,19 | 0.0020 |
|  |  |  |  |  |  |  |  |  |  |  |  |  |  |  |  | 16,16 | 0.0100 |
|  |  |  |  |  |  |  |  |  |  |  |  |  |  |  |  | 16,17 | 0.0360 |
|  |  |  |  |  |  |  |  |  |  |  |  |  |  |  |  | 16,18 | 0.0260 |
|  |  |  |  |  |  |  |  |  |  |  |  |  |  |  |  | 16,19 | 0.0080 |
|  |  |  |  |  |  |  |  |  |  |  |  |  |  |  |  | 16,20 | 0.0040 |
|  |  |  |  |  |  |  |  |  |  |  |  |  |  |  |  | 17,17 | 0.0260 |
|  |  |  |  |  |  |  |  |  |  |  |  |  |  |  |  | 17,18 | 0.0120 |
|  |  |  |  |  |  |  |  |  |  |  |  |  |  |  |  | 18,18 | 0.0020 |
|  |  |  |  |  |  |  |  |  |  |  |  |  |  |  |  | 18,19 | 0.0080 |
|  |  |  |  |  |  |  |  |  |  |  |  |  |  |  |  | 18,20 | 0.0060 |
|  |  |  |  |  |  |  |  |  |  |  |  |  |  |  |  | 19,19 | 0.0100 |

**S1 File Table B.** Allele frequencies of the 17 Y-STR loci for the Northern Iraq Arab population (*n*=102).

| Allele | DYS19 | DYS389I | DYS389II | DYS390 | DYS391 | DYS392 | DYS393 | DYS437 | DYS438 | DYS439 | DYS448 | DYS456 | DYS458 | DYS635 | Y_GATA_H4 | Genotypes | DYS385a/b |
| --- | --- | --- | --- | --- | --- | --- | --- | --- | --- | --- | --- | --- | --- | --- | --- | --- | --- |
| 9 |  |  |  |  |  |  |  |  | 0.2255 |  |  |  |  |  | 0.0098 | 9,11 | 0.0098 |
| 10 |  |  |  |  |  | 0.0098 |  |  | 0.5784 | 0.1373 |  |  |  |  | 0.0980 | 11,11 | 0.0098 |
| 10,13 |  | 0.0098 |  |  | 0.0098 |  |  |  |  |  |  |  |  |  |  | 11,12 | 0.0098 |
| 11 |  | 0.0196 |  |  | 0.0196 | 0.7549 | 0.0392 |  | 0.1667 | 0.4902 |  |  |  |  | 0.5490 | 11,13 | 0.0098 |
| 11,12 |  |  |  |  |  |  |  |  |  | 0.0098 |  |  |  |  |  | 11,14 | 0.1078 |
| 12 |  | 0.1176 |  |  | 0.1176 | 0.0294 | 0.5588 |  | 0.0098 | 0.2745 |  | 0.0098 | 0.0098 |  | 0.2157 | 11,15 | 0.0098 |
| 13 | 0.0882 | 0.6176 |  |  | 0.6176 | 0.1176 | 0.3333 |  | 0.0196 | 0.0784 |  | 0.0784 |  |  | 0.1275 | 11,16 | 0.0098 |
| 14 | 0.6275 | 0.2059 |  |  | 0.2059 | 0.0490 | 0.0588 | 0.7647 |  | 0.0098 |  | 0.2549 | 0.0294 |  |  | 12,14 | 0.0196 |
| 15 | 0.1667 | 0.0294 |  |  | 0.0294 | 0.0392 | 0.0098 | 0.1863 |  |  |  | 0.4118 | 0.1569 |  |  | 12,15 | 0.0490 |
| 16 | 0.1078 |  |  |  |  |  |  | 0.0490 |  |  |  | 0.1961 | 0.2059 |  |  | 12,16 | 0.0098 |
| 17 | 0.0098 |  |  |  |  |  |  |  |  |  |  | 0.0392 | 0.1373 |  |  | 12,17 | 0.0294 |
| 17.2 |  |  |  |  |  |  |  |  |  |  |  |  | 0.0392 |  |  | 12,20 | 0.0098 |
| 18 |  |  |  |  |  |  |  |  |  |  |  |  | 0.0784 |  |  | 12,22 | 0.0196 |
| 18,2 |  |  |  |  |  |  |  |  |  |  |  |  | 0.2353 |  |  | 13,15 | 0.0196 |
| 19 |  |  |  |  |  |  |  |  |  |  | 0.2745 | 0.0098 |  |  |  | 13,16 | 0.0294 |
| 19.2 |  |  |  |  |  |  |  |  |  |  |  |  | 0.0784 |  |  | 13,17 | 0.0392 |
| 20 |  |  |  |  |  |  |  |  |  |  | 0.5882 |  |  | 0.0980 |  | 13,18 | 0.1078 |
| 20.2 |  |  |  |  |  |  |  |  |  |  |  |  | 0.0196 |  |  | 13,19 | 0.1863 |
| 21 |  |  |  | 0.0098 |  |  |  |  |  |  | 0.1373 |  |  | 0.4118 |  | 13,20 | 0.0196 |
| 21.2 |  |  |  |  |  |  |  |  |  |  |  |  | 0.0098 |  |  | 14,14 | 0.0098 |
| 22 |  |  |  | 0.1373 |  |  |  |  |  |  |  |  |  | 0.2255 |  | 14,16 | 0.0784 |
| 23 |  |  |  | 0.5784 |  |  |  |  |  |  |  |  |  | 0.1667 |  | 14,17 | 0.0098 |
| 24 |  |  |  | 0.1471 |  |  |  |  |  |  |  |  |  | 0.0784 |  | 14,18 | 0.0098 |
| 25 |  |  |  | 0.1078 |  |  |  |  |  |  |  |  |  | 0.0196 |  | 14,19 | 0.0196 |
| 26 |  |  | 0.0196 | 0.0196 |  |  |  |  |  |  |  |  |  |  |  | 15,16 | 0.0490 |
| 27 |  |  | 0.0196 |  |  |  |  |  |  |  |  |  |  |  |  | 15,19 | 0.0098 |
| 28 |  |  | 0.0490 |  |  |  |  |  |  |  |  |  |  |  |  | 16,17 | 0.0294 |
| 29 |  |  | 0.3627 |  |  |  |  |  |  |  |  |  |  |  |  | 16,18 | 0.0098 |
| 30 |  |  | 0.4118 |  |  |  |  |  |  |  |  |  |  |  |  | 16,19 | 0.0098 |
| 31 |  |  | 0.1176 |  |  |  |  |  |  |  |  |  |  |  |  | 16,20 | 0.0098 |
| 32 |  |  | 0.0196 |  |  |  |  |  |  |  |  |  |  |  |  | 17,18 | 0.0098 |
|  |  |  |  |  |  |  |  |  |  |  |  |  |  |  |  | 18,18 | 0.0098 |
|  |  |  |  |  |  |  |  |  |  |  |  |  |  |  |  | 18,19 | 0.0098 |
|  |  |  |  |  |  |  |  |  |  |  |  |  |  |  |  | 18,20 | 0.0098 |
|  |  |  |  |  |  |  |  |  |  |  |  |  |  |  |  | 19,19 | 0.0098 |
|  |  |  |  |  |  |  |  |  |  |  |  |  |  |  |  |  |  |

**S1 File Table C.** Allele frequencies of the 17 Y-STR loci for the Northern Iraq Kurdish population (*n*=104).

| Allele | DYS19 | DYS389I | DYS389II | DYS390 | DYS391 | DYS392 | DYS393 | DYS437 | DYS438 | DYS439 | DYS448 | DYS456 | DYS458 | DYS635 | Y_GATA_H4 | Genotypes | DYS385a/b |
| --- | --- | --- | --- | --- | --- | --- | --- | --- | --- | --- | --- | --- | --- | --- | --- | --- | --- |
| 8 |  |  |  |  |  |  |  |  |  |  |  |  |  |  | 0.0096 | 9,11 | 0.0096 |
| 9 |  |  |  |  | 0.0865 |  |  |  | 0.2692 |  |  |  |  |  |  | 9,17 | 0.0096 |
| 10 |  |  |  |  | 0.6923 | 0.0481 |  |  | 0.4615 | 0.2212 |  |  |  |  | 0.0962 | 10,14 | 0.0096 |
| 11 |  |  |  |  | 0.2212 | 0.8173 | 0.0192 |  | 0.2308 | 0.3365 |  |  |  |  | 0.4423 | 11,14 | 0.1154 |
| 12 |  | 0.3173 |  |  |  | 0.0385 | 0.4904 |  | 0.0385 | 0.2885 |  | 0.0096 |  |  | 0.3462 | 11,15 | 0.0577 |
| 13 | 0.1827 | 0.5000 |  |  |  | 0.0288 | 0.3173 |  |  | 0.1250 |  |  |  |  | 0.0865 | 11,16 | 0.0096 |
| 14 | 0.2981 | 0.1827 |  |  |  | 0.0385 | 0.1538 | 0.6058 |  | 0.0288 |  | 0.0769 | 0.0192 |  | 0.0192 | 11,18 | 0.0096 |
| 15 | 0.3846 |  |  |  |  | 0.0288 | 0.0192 | 0.3173 |  |  |  | 0.6538 | 0.1827 |  |  | 11,19 | 0.0385 |
| 16 | 0.0769 |  |  |  |  |  |  | 0.0769 |  |  |  | 0.1923 | 0.3173 |  |  | 12,12 | 0.0192 |
| 16.4 |  |  |  |  |  |  |  |  |  |  | 0.0096 |  |  |  |  | 12,13 | 0.0096 |
| 17 | 0.0577 |  |  |  |  |  |  |  |  |  | 0.0192 | 0.0673 | 0.1827 |  |  | 12,14 | 0.0288 |
| 17.2 |  |  |  |  |  |  |  |  |  |  |  |  | 0.0192 |  |  | 12,16 | 0.0096 |
| 18 |  |  |  |  |  |  |  |  |  |  | 0.0288 |  | 0.0577 |  |  | 12,18 | 0.0192 |
| 18.2 |  |  |  |  |  |  |  |  |  |  |  |  | 0.0865 |  |  | 12,19 | 0.0192 |
| 19 |  |  |  |  |  |  |  |  |  |  | 0.1635 |  | 0.0481 |  |  | 13,14 | 0.0096 |
| 19.2 |  |  |  |  |  |  |  |  |  |  |  |  | 0.0481 |  |  | 13,15 | 0.0385 |
| 20 |  |  |  |  |  |  |  |  |  |  | 0.6635 |  | 0.0192 | 0.1058 |  | 13,16 | 0.0962 |
| 20.2 |  |  |  |  |  |  |  |  |  |  |  |  | 0.0096 |  |  | 13,17 | 0.0288 |
| 21 |  |  |  | 0.0096 |  |  |  |  |  |  | 0.0769 |  | 0.0096 | 0.3173 |  | 13,18 | 0.0288 |
| 22 |  |  |  | 0.1731 |  |  |  |  |  |  | 0.0288 |  |  | 0.1442 |  | 13,19 | 0.0673 |
| 23 |  |  |  | 0.3654 |  |  |  |  |  |  | 0.0096 |  |  | 0.2981 |  | 13,20 | 0.0192 |
| 24 |  |  |  | 0.2788 |  |  |  |  |  |  |  |  |  | 0.0769 |  | 13,22 | 0.0096 |
| 25 |  |  |  | 0.1635 |  |  |  |  |  |  |  |  |  | 0.0481 |  | 14,14 | 0.0096 |
| 26 |  |  | 0.0096 | 0.0096 |  |  |  |  |  |  |  |  |  | 0.0096 |  | 14,15 | 0.0096 |
| 27 |  |  | 0.0192 |  |  |  |  |  |  |  |  |  |  |  |  | 14,16 | 0.0481 |
| 28 |  |  | 0.0769 |  |  |  |  |  |  |  |  |  |  |  |  | 14,18 | 0.0288 |
| 29 |  |  | 0.3365 |  |  |  |  |  |  |  |  |  |  |  |  | 14,19 | 0.0096 |
| 30 |  |  | 0.3654 |  |  |  |  |  |  |  |  |  |  |  |  | 14,21 | 0.0096 |
| 31 |  |  | 0.1346 |  |  |  |  |  |  |  |  |  |  |  |  | 15,15 | 0.0096 |
| 32 |  |  | 0.0481 |  |  |  |  |  |  |  |  |  |  |  |  | 15,16 | 0.0096 |
| 33 |  |  | 0.0096 |  |  |  |  |  |  |  |  |  |  |  |  | 15,17 | 0.0096 |
|  |  |  |  |  |  |  |  |  |  |  |  |  |  |  |  | 15,18 | 0.0096 |
|  |  |  |  |  |  |  |  |  |  |  |  |  |  |  |  | 15.2, 17 | 0.0192 |
|  |  |  |  |  |  |  |  |  |  |  |  |  |  |  |  | 16,16 | 0.0096 |
|  |  |  |  |  |  |  |  |  |  |  |  |  |  |  |  | 16,17 | 0.0577 |
|  |  |  |  |  |  |  |  |  |  |  |  |  |  |  |  | 16,18 | 0.0192 |
|  |  |  |  |  |  |  |  |  |  |  |  |  |  |  |  | 16,19 | 0.0096 |
|  |  |  |  |  |  |  |  |  |  |  |  |  |  |  |  | 16,20 | 0.0096 |
|  |  |  |  |  |  |  |  |  |  |  |  |  |  |  |  | 18,19 | 0.0096 |
|  |  |  |  |  |  |  |  |  |  |  |  |  |  |  |  | 18,20 | 0.0192 |
|  |  |  |  |  |  |  |  |  |  |  |  |  |  |  |  | 19,19 | 0.0288 |

**S1 File Table D.** Allele frequencies of the 17 Y-STR loci for the Northern Iraq Syriac population (*n*=86).

| Allele | DYS19 | DYS389I | DYS389II | DYS390 | DYS391 | DYS392 | DYS393 | DYS437 | DYS438 | DYS439 | DYS448 | DYS456 | DYS458 | DYS635 | Y_GATA_H4 | Genotype | DYS385a/b |
| --- | --- | --- | --- | --- | --- | --- | --- | --- | --- | --- | --- | --- | --- | --- | --- | --- | --- |
| 8,11 |  |  |  |  |  |  |  |  |  | 0.0116 |  |  |  |  |  | 11,13 | 0.2093 |
| 9 |  |  |  |  |  |  |  |  | 0.4186 |  |  |  |  |  |  | 11,14 | 0.0581 |
| 10 |  |  |  |  | 0.7907 |  |  |  | 0.1744 |  |  |  |  |  | 0.0349 | 11,15 | 0.1395 |
| 11 |  |  |  |  | 0.2093 | 0.4186 | 0.0116 |  | 0.1047 | 0.3953 |  |  |  |  | 0.5581 | 12,12 | 0.0116 |
| 11,13 |  | 0.0116 |  |  |  |  |  |  |  |  |  |  |  |  |  | 12,15 | 0.0116 |
| 12 |  | 0.1047 |  |  |  | 0.0814 | 0.7442 |  | 0.3023 | 0.5349 |  | 0.0116 | 0.0233 |  | 0.3372 | 12,17 | 0.0116 |
| 13 | 0.0581 | 0.5814 |  |  |  | 0.3721 | 0.2442 |  |  | 0.0465 |  | 0.0116 |  |  | 0.0698 | 12,18 | 0.0814 |
| 14 | 0.7791 | 0.2791 |  |  |  | 0.1163 |  | 0.4767 |  | 0.0116 |  | 0.0233 |  |  |  | 13,16 | 0.0930 |
| 15 | 0.0698 | 0.0233 |  |  |  |  |  | 0.5000 |  |  |  | 0.5698 | 0.3140 |  |  | 13,17 | 0.0698 |
| 16 | 0.0698 |  |  |  |  |  |  | 0.0233 |  |  |  | 0.3372 | 0.0930 |  |  | 13,18 | 0.0233 |
| 17 | 0.0233 |  |  |  |  |  |  |  |  |  |  | 0.0465 |  |  |  | 13,19 | 0.0116 |
| 17.2 |  |  |  |  |  |  |  |  |  |  |  |  | 0.3721 |  |  | 14,15 | 0.0233 |
| 18 |  |  |  |  |  |  |  |  |  |  | 0.0116 |  | 0.0349 |  |  | 14,16 | 0.1279 |
| 18.2 |  |  |  |  |  |  |  |  |  |  |  |  | 0.0233 |  |  | 14,17 | 0.0233 |
| 19 |  |  |  |  |  |  |  |  |  |  | 0.6279 |  | 0.0349 |  |  | 14,18 | 0.0116 |
| 19.2 |  |  |  |  |  |  |  |  |  |  |  |  | 0.0581 |  |  | 14,19 | 0.0349 |
| 19,20 |  |  |  |  |  |  |  |  |  |  |  |  | 0.0465 |  |  | 15,16 | 0.0116 |
| 20 |  |  |  |  |  |  |  |  |  |  | 0.1628 |  |  | 0.0581 |  | 15,17 | 0.0349 |
| 21 |  |  |  | 0.0116 |  |  |  |  |  |  | 0.1279 |  |  | 0.3256 |  | 16,17 | 0.0116 |
| 22 |  |  |  | 0.1628 |  |  |  |  |  |  | 0.0581 |  |  | 0.1395 |  |  |  |
| 23 |  |  |  | 0.4767 |  |  |  |  |  |  |  |  |  | 0.3372 |  |  |  |
| 24 |  |  |  | 0.2674 |  |  |  |  |  |  |  |  |  | 0.1395 |  |  |  |
| 25 |  |  |  | 0.0814 |  |  |  |  |  |  |  |  |  |  |  |  |  |
| 25,29 |  |  | 0.0116 |  |  |  |  |  |  |  |  |  |  |  |  |  |  |
| 28 |  |  | 0.1977 |  |  |  |  |  |  |  |  |  |  |  |  |  |  |
| 29 |  |  | 0.3372 |  |  |  |  |  |  |  |  |  |  |  |  |  |  |
| 30 |  |  | 0.3256 |  |  |  |  |  |  |  |  |  |  |  |  |  |  |
| 31 |  |  | 0.0930 |  |  |  |  |  |  |  |  |  |  |  |  |  |  |
| 32 |  |  | 0.0349 |  |  |  |  |  |  |  |  |  |  |  |  |  |  |
| Null |  |  |  |  |  | 0.0116 |  |  |  |  | 0.0116 |  |  |  |  |  |  |

**S1 File Table E.** Allele frequencies of the 17 Y-STR loci for the Northern Iraq Turkmen population (*n*=102).

| Allele | DYS19 | DYS389I | DYS389II | DYS390 | DYS391 | DYS392 | DYS393 | DYS437 | DYS438 | DYS439 | DYS448 | DYS456 | DYS458 | DYS635 | Y_GATA_H4 | Genotype | DYS385a/b |
| --- | --- | --- | --- | --- | --- | --- | --- | --- | --- | --- | --- | --- | --- | --- | --- | --- | --- |
| 9 |  |  |  |  | 0.0784 |  |  |  | 0.2745 |  |  |  |  |  |  | 9,11 | 0.0294 |
| 10 |  |  |  |  | 0.6471 | 0.0294 |  |  | 0.4216 | 0.1373 |  |  |  |  | 0.1667 | 11,12 | 0.0196 |
| 11 |  |  |  |  | 0.2549 | 0.7549 | 0.0294 |  | 0.2157 | 0.3529 |  |  |  |  | 0.3529 | 11,13 | 0.0098 |
| 12 | 0.0098 | 0.2745 |  |  | 0.0098 | 0.0294 | 0.4510 |  | 0.0882 | 0.4510 |  |  |  |  | 0.3529 | 11,14 | 0.1176 |
| 13 | 0.2059 | 0.4118 |  |  | 0.0098 | 0.1471 | 0.4314 |  |  | 0.0588 |  |  |  |  | 0.1176 | 11,15 | 0.0098 |
| 14 | 0.3922 | 0.3137 |  |  |  | 0.0196 | 0.0784 | 0.6569 |  |  |  | 0.0686 | 0.0490 |  | 0.0098 | 11,16 | 0.0098 |
| 15 | 0.3039 |  |  |  |  | 0.0196 | 0.0098 | 0.2059 |  |  |  | 0.5392 | 0.2059 |  |  | 12,12 | 0.0098 |
| 16 | 0.0588 |  |  |  |  |  |  | 0.1373 |  |  |  | 0.3137 | 0.3137 |  |  | 12,13 | 0.0098 |
| 16.4 |  |  |  |  |  |  |  |  |  |  | 0.0098 |  |  |  |  | 12,14 | 0.0098 |
| 17 | 0.0294 |  |  |  |  |  |  |  |  |  | 0.0098 | 0.0686 | 0.2549 |  |  | 12,15 | 0.0196 |
| 17.2 |  |  |  |  |  |  |  |  |  |  |  |  | 0.0098 |  |  | 12,16 | 0.0196 |
| 18 |  |  |  |  |  |  |  |  |  |  | 0.0196 |  | 0.0392 |  |  | 12,18 | 0.0588 |
| 18.2 |  |  |  |  |  |  |  |  |  |  |  |  | 0.0588 |  |  | 12,19 | 0.0098 |
| 19 |  |  |  |  |  |  |  |  |  |  | 0.2549 |  |  |  |  | 12,20 | 0.0196 |
| 19.2 |  |  |  |  |  |  |  |  |  |  |  |  | 0.0196 |  |  | 13,13 | 0.0098 |
| 20 |  |  |  |  |  |  |  |  |  |  | 0.4412 |  |  | 0.0882 |  | 13,14 | 0.0098 |
| 20.2 |  |  |  |  |  |  |  |  |  |  |  |  | 0.0392 |  |  | 13,15 | 0.0392 |
| 21 |  |  |  | 0.0490 |  |  |  |  |  |  | 0.2157 |  | 0.0098 | 0.3137 |  | 13,16 | 0.0588 |
| 22 |  |  |  | 0.1373 |  |  |  |  |  |  |  |  |  | 0.2353 |  | 13,17 | 0.0490 |
| 23 |  |  |  | 0.4314 |  |  |  |  |  |  | 0.0098 |  |  | 0.2941 |  | 13,18 | 0.0294 |
| 24 |  |  |  | 0.1961 |  |  |  |  |  |  | 0.0392 |  |  | 0.0294 |  | 13,19 | 0.0196 |
| 25 |  |  |  | 0.1765 |  |  |  |  |  |  |  |  |  | 0.0392 |  | 14,14 | 0.0098 |
| 26 |  |  |  | 0.0098 |  |  |  |  |  |  |  |  |  |  |  | 14,15 | 0.0784 |
| 28 |  |  | 0.1078 |  |  |  |  |  |  |  |  |  |  |  |  | 14,16 | 0.0294 |
| 29 |  |  | 0.2255 |  |  |  |  |  |  |  |  |  |  |  |  | 14,17 | 0.0196 |
| 30 |  |  | 0.3529 |  |  |  |  |  |  |  |  |  |  |  |  | 14,18 | 0.0392 |
| 30.3 |  |  |  |  |  |  |  |  |  |  |  |  |  |  |  | 14,19 | 0.0098 |
| 31 |  |  | 0.1275 |  |  |  |  |  |  |  |  |  |  |  |  | 15,15 | 0.0098 |
| 32 |  |  | 0.1863 |  |  |  |  |  |  |  |  |  |  |  |  | 15,16 | 0.0098 |
| Null |  |  |  |  |  |  |  |  |  |  |  | 0.0098 |  |  |  | 15,17 | 0.0196 |
|  |  |  |  |  |  |  |  |  |  |  |  |  |  |  |  | 16,16 | 0.0392 |
|  |  |  |  |  |  |  |  |  |  |  |  |  |  |  |  | 16,17 | 0.0784 |
|  |  |  |  |  |  |  |  |  |  |  |  |  |  |  |  | 16,18 | 0.0196 |
|  |  |  |  |  |  |  |  |  |  |  |  |  |  |  |  | 16,19 | 0.0196 |
|  |  |  |  |  |  |  |  |  |  |  |  |  |  |  |  | 17,17 | 0.0294 |
|  |  |  |  |  |  |  |  |  |  |  |  |  |  |  |  | 17,18 | 0.0098 |
|  |  |  |  |  |  |  |  |  |  |  |  |  |  |  |  | 19,19 | 0.0098 |

**S1 File Table F.** Allele frequencies of the 17 Y-STR loci for the Northern Iraq Yazidi population (*n*=106).

| Allele | DYS19 | DYS389I | DYS389II | DYS390 | DYS391 | DYS392 | DYS393 | DYS437 | DYS438 | DYS439 | DYS448 | DYS456 | DYS458 | DYS635 | Y_GATA_H4 | Genotype | DYS385a/b |
| --- | --- | --- | --- | --- | --- | --- | --- | --- | --- | --- | --- | --- | --- | --- | --- | --- | --- |
| 8 |  |  |  |  |  |  |  |  | 0.0094 |  |  |  |  |  |  | 11,11 | 0.0094 |
| 9 |  |  |  |  |  |  |  |  | 0.2170 |  |  |  |  |  |  | 11,13 | 0.0189 |
| 10 |  |  |  |  | 0.7075 | 0.0283 | 0.0094 |  | 0.4528 | 0.0943 |  |  |  |  | 0.0566 | 11,14 | 0.1698 |
| 11 |  |  |  |  | 0.2170 | 0.5755 |  |  | 0.1509 | 0.4434 |  |  |  |  | 0.3019 | 11,15 | 0.0377 |
| 12 |  | 0.1226 |  |  | 0.0755 |  | 0.4811 |  | 0.1698 | 0.3585 |  |  |  |  | 0.5660 | 12,12 | 0.0472 |
| 13 | 0.0755 | 0.6887 |  |  |  | 0.1321 | 0.3679 |  |  | 0.0566 |  | 0.0189 |  |  | 0.0755 | 12,14 | 0.0283 |
| 14 | 0.6509 | 0.1887 |  |  |  | 0.2547 | 0.1415 | 0.4528 |  | 0.0472 |  | 0.1509 | 0.0472 |  |  | 12,15 | 0.0094 |
| 14,16 | 0.0094 |  |  |  |  |  |  |  |  |  |  |  |  |  |  | 12,16 | 0.0283 |
| 15 | 0.1509 |  |  |  |  |  |  | 0.3396 |  |  |  | 0.4245 | 0.2642 |  |  | 12,17 | 0.0094 |
| 16 | 0.1132 |  |  |  |  |  |  | 0.1509 |  |  |  | 0.3679 | 0.1415 |  |  | 12,18 | 0.0283 |
| 17 |  |  |  |  |  |  |  | 0.0566 |  |  |  |  | 0.2547 |  |  | 12,20 | 0.0283 |
| 18 |  |  |  |  |  |  |  |  |  |  | 0.0189 | 0.0377 | 0.2170 |  |  | 13,16 | 0.0472 |
| 18.2 |  |  |  |  |  |  |  |  |  |  |  |  | 0.0660 |  |  | 13,17 | 0.0094 |
| 19 |  |  |  |  |  |  |  |  |  |  | 0.4245 |  | 0.0094 |  |  | 13,19 | 0.0094 |
| 19,20 |  |  |  |  |  |  |  |  |  |  | 0.0472 |  |  |  |  | 14,14 | 0.0094 |
| 20 |  |  |  |  |  |  |  |  |  |  | 0.3774 |  |  | 0.1698 |  | 14,15 | 0.0189 |
| 21 |  |  |  | 0.0472 |  |  |  |  |  |  | 0.0943 |  |  | 0.3208 |  | 14,16 | 0.0377 |
| 21,22 |  |  |  |  |  |  |  |  |  |  | 0.0283 |  |  |  |  | 14,20 | 0.0566 |
| 22 |  |  |  | 0.1415 |  |  |  |  |  |  |  |  |  | 0.0566 |  | 15,15 | 0.0283 |
| 23 |  |  |  | 0.3491 |  |  |  |  |  |  |  |  |  | 0.2736 |  | 15,16 | 0.0755 |
| 24 |  |  |  | 0.3208 |  |  |  |  |  |  |  |  |  | 0.1792 |  | 15,17 | 0.0660 |
| 25 |  |  |  | 0.1415 |  |  |  |  |  |  |  |  |  |  |  | 16,18 | 0.0755 |
| 25,29 |  |  |  |  |  |  |  |  |  |  |  |  |  |  |  | 17,17 | 0.0943 |
| 28 |  |  | 0.1509 |  |  |  |  |  |  |  |  |  |  |  |  | 17,18 | 0.0377 |
| 29 |  |  | 0.3302 |  |  |  |  |  |  |  |  |  |  |  |  | 18,19 | 0.0189 |
| 30 |  |  | 0.4245 |  |  |  |  |  |  |  |  |  |  |  |  |  |  |
| 30.3 |  |  | 0.0283 |  |  |  |  |  |  |  |  |  |  |  |  |  |  |
| 31 |  |  | 0.0377 |  |  |  |  |  |  |  |  |  |  |  |  |  |  |
| 32 |  |  | 0.0283 |  |  |  |  |  |  |  |  |  |  |  |  |  |  |
| Null |  |  |  |  |  | 0.0094 |  |  |  |  | 0.0094 |  |  |  |  |  |  |
